# Alterations in chromatin organization promote totipotent-like features in a DPPA2/DUX-dependent manner

**DOI:** 10.1038/s44318-026-00853-6

**Published:** 2026-07-09

**Authors:** Grace I Carey, Maria Vega-Sendino, Desiree Tillo, Sarah C Hoelscher, Amir Khan, Ian Taukulis, Teresa Olbrich, Bechara Saykali, Andy D Tran, Bhavya Soni, Anna Lee Fong, Melissa Mikolaj, Adam Harned, Kedar Narayan, Michael J Kruhlak, Michael Kelly, Ning Qing Liu, Elzo de Wit, Sergio Ruiz

**Affiliations:** 1 Laboratory of Genome Integrity, CCR, NCI, NIH, Bethesda, MD USA; 2 CCR Genomics Core, CCR, NCI, NIH, Bethesda, MD USA; 3 Single Cell Analysis Facility, NCI, NIH, Bethesda, MD USA; 4 Cancer Research Technology Program, Frederick National Laboratory for Cancer Research, Frederick, MD USA; 5 Laboratory of Cancer Biology and Genetics, CCR, NCI, NIH, Bethesda, MD USA; 6 CCR Volume Electron Microscopy (CVEM), CCR, NCI, NIH, Bethesda, MD USA; 7 Department of Hematology, Erasmus Medical Center Cancer Institute, Rotterdam, The Netherlands; 8 Oncode Institute and Division of Gene Regulation, Netherlands Cancer Institute, Amsterdam, The Netherlands

**Keywords:** Chromatin, Transcription & Genomics, Development, Stem Cells & Regenerative Medicine

## Abstract

2-cell like cells (2CLC) are a transiently cycling population of cells with totipotent-associated features. Although CTCF depletion induces 2CLC conversion in mouse ESC, whether this reprogramming is a consequence of disrupted higher-order chromatin organization or of CTCF-specific functions remained unclear. Here, we show that depletion of the cohesin release factor WAPL in ESC also promotes 2CLC reprogramming, which is increased by CTCF co-depletion. Single-cell RNA-seq/ATAC-seq analyses in CTCF/WAPL-depleted ESC revealed that chromatin accessibility precedes 2C-associated gene expression. Moreover, we identified ARID3A as a transcription factor that regulates the extent of 2CLC conversion following WAPL/CTCF depletion. Although WAPL or CTCF depletion induces distinct transcriptional changes in human ESC, these do not resemble transcriptional programs of early human embryogenesis, suggesting limited evolutionary conservation. Finally, we demonstrate that 2CLC conversion mediated by alterations in chromatin organization depends on the DPPA2/DUX axis and correlates with nucleolar integrity. Together, these findings establish a mechanistic link between higher-order chromatin organization and totipotency-like cell identity in mice.

## Synopsis

Higher-order chromatin organization has been implicated in the regulation of totipotency, but the mechanisms linking chromatin architecture to totipotent-like cell states remain poorly understood. This study shows that disruption of chromatin organization by degron-mediated CTCF and/or WAPL depletion promotes totipotent-like conversion through the DPPA2/DUX axis, accompanied by changes in chromatin accessibility and nucleolar integrity.

- WAPL depletion promotes 2CLC reprogramming in mouse ESC, and is enhanced by CTCF co-depletion.
- The transcription factor ARID3A limits the extent of 2CLC conversion following CTCF/WAPL-depletion.
- Increased chromatin accessibility precedes expression of 2C-associated transcripts following CTCF/WAPL depletion.
- 2CLC conversion induced by CTCF/WAPL depletion depends on the DPPA2/DUX axis and correlates with nucleolar integrity.
- WAPL or CTCF depletion in human ESC fails to activate transcriptional programs characteristic of early human embryogenesis, suggesting limited evolutionary conservation.

## Abstract (web summary)

Chromatin architectural defects trigger the DPPA2/DUX program and nucleolar remodeling to promote totipotent-like cell conversion in mouse embryonic stem cells.

## Graphical abstract

**Figure:**
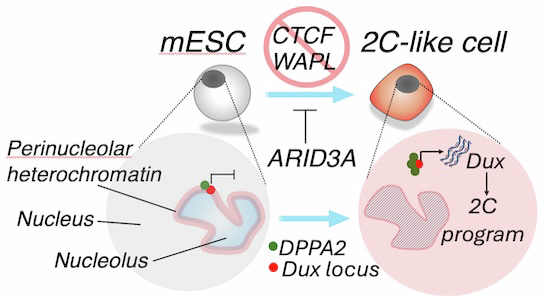

**Figure:**
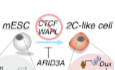

## Introduction

The unlimited potential of a totipotent zygote that can generate both embryonic and extra-embryonic tissues becomes progressively more restricted as the embryo develops. In mice, totipotency is restricted to the zygote and blastomeres from the 2-cell (2C) stage (Lu and Zhang, 2015; Riveiro and Brickman, 2020). Zygotic genome activation (ZGA) at the 2C-stage seems to be the trigger promoting the exit from totipotency (Schultz and Harrison, 2019). ZGA leads to the activation of a transcriptional program unique to the totipotent 2C stage, including the expression of cleavage stage-specific genes (e.g.,* Dux*, members of the *Zscan4* cluster and *Zfp352*) and the murine endogenous retroviral element (MERVL) family of transposable elements (Hendrickson et al, 2017; De Iaco et al, 2017; Whiddon et al, 2017). Importantly, the 2C-associated transcriptional program is quickly silenced following the exit from totipotency at the 4-cell (4C) stage.

Embryonic stem cells (ESC) are a useful model to study totipotency-associated features. A transiently cycling population of cells with totipotent potential (around 1–2%) was identified in mouse ESC (mESC) cultures (Macfarlan et al, 2012; Genet and Torres-Padilla, 2020). These 2C-like cells (2CLC) express high levels of transcripts detected in the 2C embryo, including MERVL elements (Macfarlan et al, 2012; Genet and Torres-Padilla, 2020). These viral relics are silenced in mESC but become derepressed in 2CLC. This specific feature has been used to genetically label 2CLC in vitro using a reporter based on MERVL promoter sequences to study their behavior and properties (Macfarlan et al, 2012; Genet and Torres-Padilla, 2020). Interestingly, the low percentage of endogenous 2CLC can be efficiently increased by the over-expression of the transcription factor DUX (Hendrickson et al, 2017; De Iaco et al, 2017; Whiddon et al, 2017). Furthermore, spontaneously reprogrammed 2CLC depend on the expression of DUX, as *Dux*-knockout mESC do not have an endogenous 2CLC population revealing the relevance of DUX in this reprogramming process (De Iaco et al, 2017).

Chromatin is distinctly folded into dynamic higher-order structures that can regulate gene expression by controlling interactions between different regions of the genome (e.g. enhancers and promoters) (Yu and Ren, 2017; Strickfaden, 2021). Following fertilization, the embryonic genome undergoes a dramatic epigenetic remodeling characterized by an extensive loss of conventional chromatin organization followed by a de novo re-establishment of the 3D organization during development (Ke et al, 2017; Du et al, 2017; Ing-Simmons et al, 2022). Because genome organization impacts the expression of genes, we and others have investigated the role of factors regulating chromatin structure in the expression of the totipotent transcriptional program (Olbrich et al, 2021; Zhu et al, 2021). This idea was supported by two observations. First, totipotent zygotes and 2C-stage embryos are characterized by an unstructured chromatin conformation with weakened compartments and topologically associated domains (TAD). These weak higher-order structures gradually strengthen during embryonic development, correlating with a progressive restriction of developmental potential (Ke et al, 2017; Du et al, 2017; Ing-Simmons et al, 2022). Second, depletion of the cohesin subunit RAD21 in differentiated cells, which disrupts higher-order chromatin structure, facilitates reprogramming to totipotency during somatic cell nuclear transfer experiments (Zhang et al, 2020). Furthermore, depletion of CTCF, a transcription factor involved in chromosome folding and insulation, causes mESC to acquire totipotency-associated features and convert to a 2CLC state (Olbrich et al, 2021; Zhu et al, 2021). Nevertheless, whether the mechanism underlying this conversion depends on CTCF-specific functions or is promoted by alterations in higher-order chromatin structure remains unknown. In this work, we show that depletion of the cohesin releasing factor WAPL promotes efficient 2CLC conversion. This reprogramming is enhanced by CTCF co-depletion but is restricted to pluripotent cells. Moreover, we also determined that the transcription factor ARID3A controls the extent of 2CLC conversion following WAPL/CTCF depletion. Furthermore, our data demonstrate that increased chromatin accessibility precedes the expression of 2C-associated genes and MERVL repeats. Finally, we show that the DPPA2/DUX axis is essential to mediate 2CLC conversion following alterations in chromatin organization and correlates with nucleolar integrity. Overall, our findings provide a framework to study how genome organization impacts the expression of genetic programs during development.

## Results

### WAPL depletion promotes spontaneous conversion to a 2CLC state

**Figure 1:**
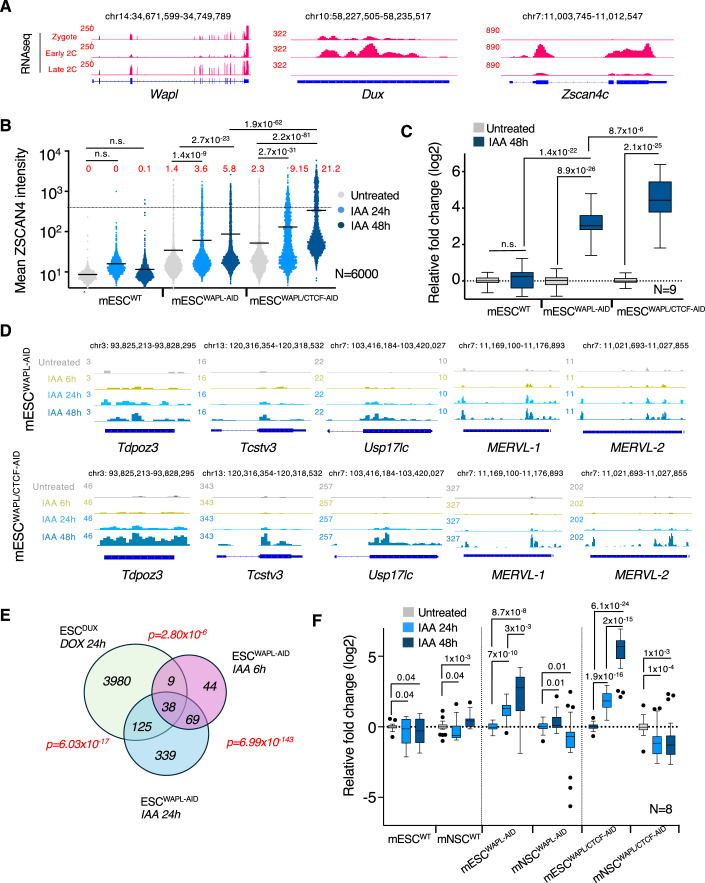
WAPL depletion leads to 2CLC conversion. (**A**) Genome browser tracks showing RNA-seq RPKM read count at the indicated genes in samples obtained from Deng et al, 2014. (**B**) High-throughput imaging quantification of ZSCAN4 mean nuclear intensity in mESC^WT^, mESC^WAPL-AID^ and mESC^WAPL/CTCF-AID^ that were untreated or treated with IAA for 24 or 48 h. Center lines indicate mean values. Numbers in red show the percentage of ZSCAN4^+^ cells above the arbitrary threshold. Three independent experiments were performed but only one representative is shown. Similar results were obtained in independent clones from the same genotype. (**C**) Real time-PCR analysis shown in a box and whisker plot showing the relative fold change (log2) expression of nine 2C-associated transcripts (*Dux, Zscan4c, Zfp352, Tcstv3, Sp110, Tdpoz1, Dub1, Eif1ad8, and MERVLs*) in mESC^WT^, mESC^WAPL-AID^ and mESC^WAPL/CTCF-AID^ that were untreated or treated with IAA for 24 or 48 h. *Gapdh* expression was used to normalize. For the box plot, center line indicates the median, box extends from the 25th to 75th percentiles and whiskers show minimum to maximum values. Two independent experiments were performed but only one representative experiment is shown. (**D**) Genome browser tracks showing RNA-seq RPKM read count at the indicated genes and repeats in samples obtained from ESC^WAPL-AID^ and ESC^WAPL/CTCF-AID^ that were untreated or treated with IAA at the indicated times. RNA-seq data was obtained from Liu et al, 2021; Liu et al, 2025. (**E**) Venn diagram showing the intersection of genes upregulated in mESC^WAPL-AID^ that were treated with IAA for the indicated times with genes upregulated upon DOX-induced DUX expression. Transient transcriptome sequencing (TT-seq) data from ESC^WAPL-AID^ was obtained from Liu et al, 2021. RNA-seq data from DUX-expressing cells was obtained from Hendrickson et al, 2017. Upregulated genes were defined based on Cutoff Log2 > 1, *P* value < 0.05. (**F**) Real time-PCR analysis shown in a box and whisker plot using the Tukey Method showing the relative fold change (log2) expression of the same transcripts as in (**C**) in mESC or mNSC of the indicated genotypes that were untreated or treated with IAA for 24 or 48 h. *Gapdh* expression was used to normalize. For the box plot, center line indicates the median, box extends from the 25th to 75th percentiles and whiskers show minimum to maximum values. Individual dots show outliers. Data information: For (**B**,** C**,** E**, **F**), *P* values are shown from one-tailed unpaired *t* tests. Source data are available online for this figure.

**Figure EV1:**
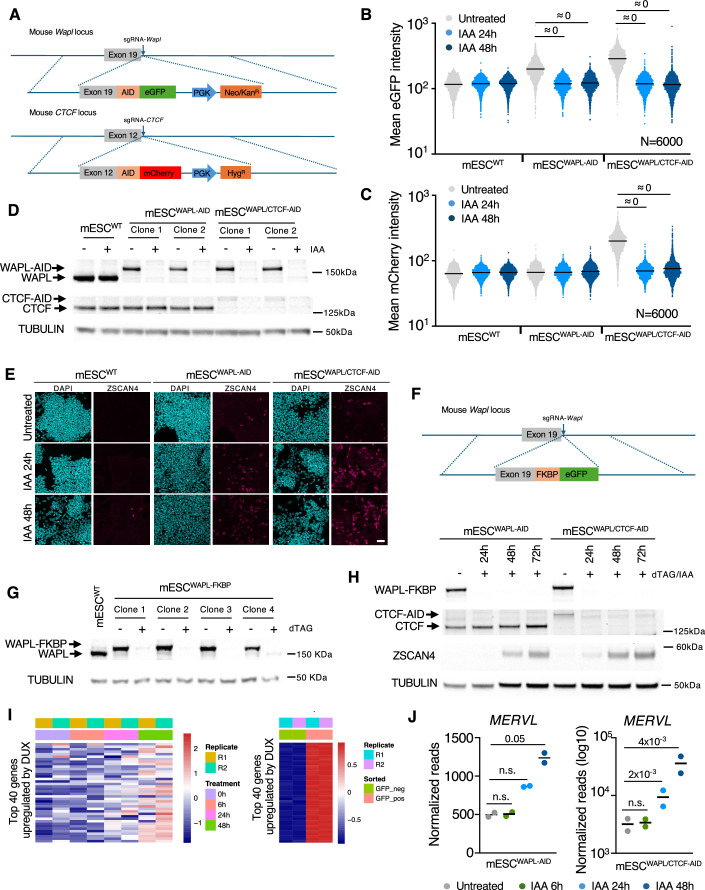
Supporting Fig. 1. 2CLC conversion is induced by the loss of WAPL or WAPL/CTCF. (**A**) Schematic representation of the targeting strategy used to generate the cell lines (mESC^WAPL-AID^ and mESC^WAPL/CTCF-AID^) used in this study. (**B**, **C**) High-throughput imaging quantification of eGFP (**C**) and mCherry (**D**) mean nuclear intensity in mESC^WT^, mESC^WAPL-AID^ and mESC^WAPL/CTCF-AID^ that were untreated or treated with IAA 24 or 48 h. Center lines indicate mean values. *P* values are shown from one-tailed unpaired *t* tests. Two independent experiments were performed but only one representative is shown. (**D**) Western blot analysis of WAPL and CTCF performed in lysates from different mESC^WT^, mESC^WAPL-AID^ and mESC^WAPL/CTCF-AID^ clones that were untreated or treated with IAA for 24 h. mESC^WT^ were included to show the increased size of the tagged WAPL and CTCF compared to their endogenous versions. TUBULIN levels are shown as a loading control. (**E**) Images cropped out from representative images showing ZSCAN4 staining from the experiment shown in Fig. 1B. Scale bar: 50 μM. (**F**) Schematic representation of the targeting strategy used to generate mESC^WAPL-FKBP^. (**G**) Western blot analysis of WAPL performed in lysates from different mESC^WAPL-FKBP^ cell clones that were untreated or treated with dTAG for 24 h. mESC^WT^ were included to show the increased size of the tagged WAPL protein. TUBULIN levels are shown as a loading control. (**H**) Western blot analysis of WAPL, CTCF and ZSCAN4 performed in lysates from a representative mESC^WAPL-FKBP^ or mESC^WAPL-FKBP/CTCF-AID^ clonal line untreated or treated with dTAG for the indicated times. TUBULIN levels are shown as a loading control. (**I**) Heatmaps generated from RNA-seq data from wild-type and DUX-expressing (right) or mESC^WAPL-AID^ that were untreated and treated with IAA at different time points (left). DUX-expressing mESC were sorted in GFP+ or GFP- based on the activation of the LTR-GFP reporter. Heatmaps show the top 40 most upregulated genes upon DUX expression based on Cutoff Log2 > 1, *P* value < 0.05. (**J**) Plots showing normalized MERVL read counts obtained from RNA-seq datasets corresponding to untreated or IAA-treated mESC^WAPL-AID^ and mESC^WAPL/CTCF-AID^ samples. Data was obtained from Liu et al, 2021, 2025. *P* values are shown from one-tailed unpaired *t* tests. Data information: For (**E**, **G**, **H**), two independent experiments were performed but one representative experiment is shown. Source data are available online for this figure.

We recently showed that CTCF-depleted mESC undergo asynchronous but efficient 2CLC conversion, which can be reverted upon restoration of CTCF levels (Olbrich et al, 2021). To clarify whether 2CLC conversion is due to structural alterations in chromatin or to non-structural CTCF-specific effects, we evaluated the role of additional proteins regulating higher-order chromatin organization in promoting totipotent-like features in mESC. Transcription levels of the cohesin release factor *Wapl* are considerably lower in the early 2C-stage embryo compared to zygotes or late 2C-stage embryos (Fig. 1A). These low levels correlate with the highest levels of *Dux* and 2C-associated markers such as *Zscan4c* in the early 2C-stage embryo (Fig. 1A). Thus, we hypothesized that depletion of WAPL might also lead to increased 2CLC conversion. To explore this possibility, we used a previously described mESC line containing an auxin-inducible degron system (Liu et al, 2021). This cell line (mESC^WAPL-AID^ hereafter) contains an auxin-inducible degron (AID) and an enhanced green fluorescent protein (eGFP) sequence inserted at the C terminus of the endogenous *Wapl* gene (Fig. EV1A) (Liu et al, 2021). Addition of indole-3-acetic acid (IAA) to the culture medium promotes a rapid degradation of WAPL (Fig. EV1B–D). Importantly, although levels of the targeted WAPL protein were lower compared to endogenous levels, untreated mESC^WAPL-AID^ show minimal transcriptomic differences compared to the parental cell line (Liu et al, 2021).

To test whether WAPL depletion induced 2CLC conversion, we examined the expression levels of *Zscan4*, a gene cluster of six paralogs (*Zscan4a-f*) that is highly expressed in the 2 C embryo as well as in 2CLC. Remarkably, WAPL depletion induced a rapid increase in ZSCAN4 expression compared to unmodified wild-type ESC (mESC^WT^ hereafter) (Figs. 1B and EV1E). We confirmed these results by generating additional degron mESC lines tagging endogenous *Wapl* with an FKBP domain (mESC^WAPL-FKBP^), resulting in rapid WAPL degradation and increased ZSCAN4 expression upon dTAG treatment (Fig. EV1F–H). We also observed increased expression in a panel of 2C-associated genes and MERVL repeats, confirming the generation of 2CLC upon WAPL depletion (Figs. 1C,D and EV1I,J). We next examined published nascent transcriptome datasets in untreated or IAA-treated mESC^WAPL-AID^ for 6 or 24 h to determine the immediate effects of WAPL depletion on the 2C-associated transcriptome (Liu et al, 2021). We found a significant overlap between the genes upregulated following WAPL depletion and those induced by DUX (Fig. 1E). This overlap was evident as early as 6 h after IAA addition, suggesting that upregulation of the 2C-associated transcriptional program might be an early event.

We next asked whether combined CTCF and WAPL depletion cooperate in promoting further reprogramming. Thus, we used a previously generated cell line where endogenous *Ctcf* was tagged with an AID-mCherry sequence in mESC^WAPL-AID^ (mESC^WAPL/CTCF-AID^ hereafter), allowing for a combined degradation upon IAA addition (Fig. EV1A–E) (Liu et al, 2025). Strikingly, CTCF and WAPL co-depletion cooperated in 2CLC induction (Figs. 1B–D and EV1H–J). This reprogramming was quicker and more efficient than depletion of WAPL alone, as the expression of 2C-associated genes was dramatically increased 24 h after CTCF/WAPL depletion compared to WAPL-depleted cells (Figs. 1B–D and EV1H–J).

We next explored whether lineage commitment imposes a barrier to prevent 2CLC conversion in the absence of WAPL or WAPL/CTCF. Indeed, we previously showed that CTCF-depleted mouse neural stem cells (mNSC) were unable to undergo 2CLC reprogramming (Olbrich et al, 2021). Thus, we differentiated the corresponding mESC to mNSC and observed that WAPL or WAPL/CTCF depletion in mNSC did not induce 2CLC conversion compared to their mESC of origin (Fig. 1F; Appendix Fig. S1). These results show that differentiation is a strong barrier preventing the acquisition of totipotent-associated features and demonstrate that transition to a 2CLC state is a property associated with pluripotent cells.

### Chromatin accessibility precedes 2C-associated gene expression following CTCF/WAPL depletion

**Figure 2:**
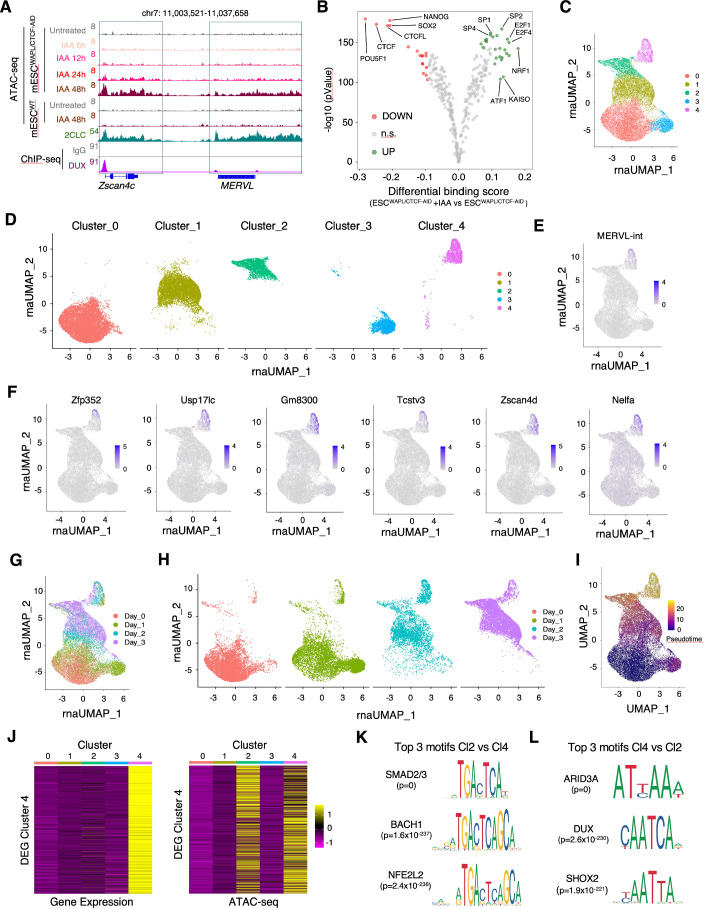
Chromatin opening precedes gene expression of the 2C-associated transcriptome. (**A**) Genome browser tracks showing ATAC-seq reads at the indicated genomic location generated from mESC^WT^ and mESC^CTCF/WAPL-AID^ lines that were untreated and treated with IAA for the indicated times. The 2CLC track shows ATAC-seq data generated from DUX-induced cells sorted based on positive LTR-RFP reporter expression. Additional tracks from published ChIP-seq data show DUX binding enrichment (Hendrickson et al, 2017). (**B**) Volcano plot showing predicted transcription factor motifs differentially enriched in regions gaining accessibility in cells from mESC^CTCF/WAPL-AID^ cell line that were treated with IAA for 72 h compared to the untreated control. Motifs are the top 5% for both *P* value and fold change in each direction. *P* values were computed by comparing observed log2 fold changes to a subsampled empirical background distribution derived from low-signal/unbound motif sites. One cell line was used for this analysis. ATAC-seq data from (**A**) was used to generate this plot. (**C**, **D**) Uniform manifold approximation and projections (UMAPs) of combined single-cell transcriptomes of mESC^CTCF/WAPL-AID^ that were untreated or treated with IAA for 24, 48 or 72 h with representative clusters identified using Seurat. (**E**, **F**) UMAP visualizations highlighting expression of MERVL elements (**E**) or a set of 2C-associated transcriptional markers (**F**). (**G**, **H**) UMAP visualizations of samples separated by treatment length. (**I**) UMAP visualization showing the inferred transcriptional trajectory in a colored pseudotime scale using Monocle. (**J**) Heatmap showing the set of genes differentially expressed in cluster 4 (left) and the level of chromatin accessibility at the proximal regions associated to the same genes in all clusters (right). (**K**, **L**) The top three enriched transcription factor motifs characterizing cells from cluster 2 versus those from cluster 4 (**K**) and vice versa (**L**) are shown.

**Figure EV2:**
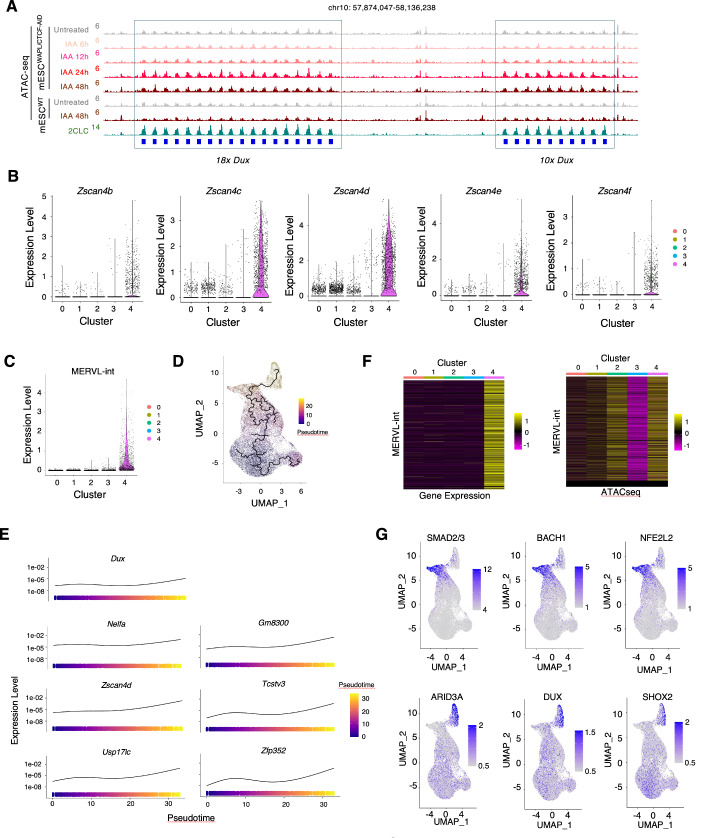
Supporting Fig. 2. WAPL/CTCF depletion leads to transcriptional and chromatin accessibility changes over time. (**A**) Genome browser tracks showing ATAC-seq reads at the genomic region containing the *Dux* cluster of genes generated from mESC^WT^ and mESC^CTCF/WAPL-AID^ lines that were untreated and IAA-treated for the indicated times. Boxed are the two main regions containing 18 and 10 *Dux* gene copies. The 2CLC track shows ATAC-seq data generated from DUX-induced cells sorted based on positive LTR-RFP reporter expression. Tracks shown were derived from one mESC^WT^ and one mESC^CTCF/WAPL-AID^ clonal cell line. (**B**, **C**) Plots showing the expression level of the indicated genes (**B**) or MERVL elements (**C**) in individual cells from corresponding identified clusters. (Cluster 0, *n* = 10123; Cluster 1, *n* = 6203; Cluster 2, *n *= 2697; Cluster 3, *n* = 1960; Cluster 4, *n* = 1444). (**D**) UMAP visualization showing the inferred transcriptional trajectory (black line) over a colored pseudotime scale. (**E**) Plots showing the expression level of the indicated genes over a colored pseudotime scale. (**F**) Heatmaps showing the level of expression (right panel) and chromatin accessibility (left panel) in all annotated MERVL-int elements in all cell clusters. (**G**) UMAP visualization showing the transcription factor motif enrichment (top 3 motifs) characterizing cells from cluster 2 versus those from cluster 4 (upper panels) and vice versa (lower panels). Source data are available online for this figure.

CTCF depletion leads to changes in chromatin accessibility in MLL-rearranged human B cell lymphoblastic leukemia cells (Xu et al, 2021). However, the effects of the combined depletion of CTCF and WAPL on chromatin accessibility in mESC have not been examined. Thus, we measured chromatin accessibility by bulk ATAC-seq in mESC^WAPL/CTCF-AID^ at 6, 12, 24 and 48 h following CTCF/WAPL co-depletion. We also included untreated and IAA-treated mESC^WT^ as well as 2CLC isolated by cell sorting using RFP levels derived from a reporter containing MERVL promoter sequences in doxycycline (DOX)-inducible DUX expressing cells (mESC^DUX^) (Olbrich et al, 2021). We observed a progressive chromatin opening over time associated with DUX-bound sites, including MERVL elements as well as at all *Dux* gene copies (Figs. 2A and EV2A; Appendix Fig. S2). Consistent with a direct role for CTCF in maintaining open chromatin, the CTCF binding motif is enriched in regions that become inaccessible in IAA-treated mESC^WAPL/CTCF-AID^ for 48 h compared to mESC^WT^ (Fig. 2B; Dataset EV1). In addition, motifs for the pluripotency factors NANOG, SOX2 and POU5F1 (OCT4) were also enriched in regions becoming inaccessible (Fig. 2B; Dataset EV1). This observation agrees with the described role for WAPL in sustaining the pluripotent network in mESC (Liu et al, 2021). Additionally, we identified transcription factor motifs, including those for SP and E2F transcription factors, that are enriched in regions that gain accessibility following CTCF/WAPL co-depletion (Fig. 2B).

Given that 2CLC conversion is highly efficient but not fully penetrant in CTCF/WAPL-depleted mESC, the use of bulk analyses might obscure important changes in different subsets of cells. Thus, we performed a single-cell multiome (ATAC-seq + RNA-seq) analysis in untreated and IAA-treated mESC^WAPL/CTCF-AID^ for 24, 48 and 72 h. UMAP plots based on RNA-seq (rnaUMAP) or ATAC-seq (atacUMAP) data as well as both combined using a weighted nearest neighbor (wnnUMAP) analysis revealed 5 distinct cell clusters within all samples using Seurat (Fig. 2C,D; Appendix Fig. S3). Cluster 4 is enriched in 2 CLC, as cells in this cluster express 2C-associated markers including members of the *Zscan4* family and MERVL elements (Figs. 2E,F and EV2B,C; Dataset EV2). rnaUMAP plots showing cells colored by sample (Day 0 to 3) revealed that the bulk of the population shifts transcriptionally over time toward a 2CLC state (Fig. 2G,H). Indeed, pseudotime analyses using Monocle (Trapnell et al, 2014), which identified similar clusters compared to Seurat, corroborated this transcriptional trend (Figs. 2I and EV2D,E; Appendix Fig. S4A). Furthermore, this analysis let us identify modules of genes that change across time and are enriched in specific cell clusters (Appendix Fig. S4B and Dataset EV3). For example, cells located in cluster 2, the cluster that is transcriptionally closer and located immediately prior to cluster 4 in the pseudotime scale, are enriched in genes involved in cell adhesion or the Hippo signaling pathway (Appendix Fig. S4C,D). This suggests that genes controlling these processes and molecular pathways could influence successful 2CLC reprogramming.

Next, we hypothesized that alterations to chromatin accessibility in proximal regions following CTCF/WAPL depletion could correlate with the activation of 2C-associated transcripts. Therefore, we selected a list of chromatin regions that were differentially accessible in cells from clusters 2 and 4 compared to the rest of the clusters and assigned those to the gene with the closest transcription start site. Interestingly, those associated with differentially expressed genes in cells from cluster 4, which correspond to 2CLC, showed negligible expression in cells from cluster 2 (Fig. 2J; Dataset EV4). Similarly, although MERVL elements showed increased chromatin accessibility in cells from clusters 2 and 4 compared to the rest of the cell clusters, only cells from cluster 4 showed elevated MERVL expression (Fig. EV2F). Transcription motif enrichment analyses over regions differentially accessible between clusters 2 vs 4 (enriched in cluster 2) or clusters 4 vs 2 (enriched in cluster 4) revealed factors that potentially could influence the conversion toward a 2CLC state (Figs. 2K,L and EV2G; Tables EV1 and 2).

In summary, these data suggest that chromatin accessibility changes precede the expression of the 2C-associated program during 2CLC conversion in CTCF/WAPL-depleted cells.

### *Arid3a* expression levels determine 2CLC conversion efficiency

**Figure EV3:**
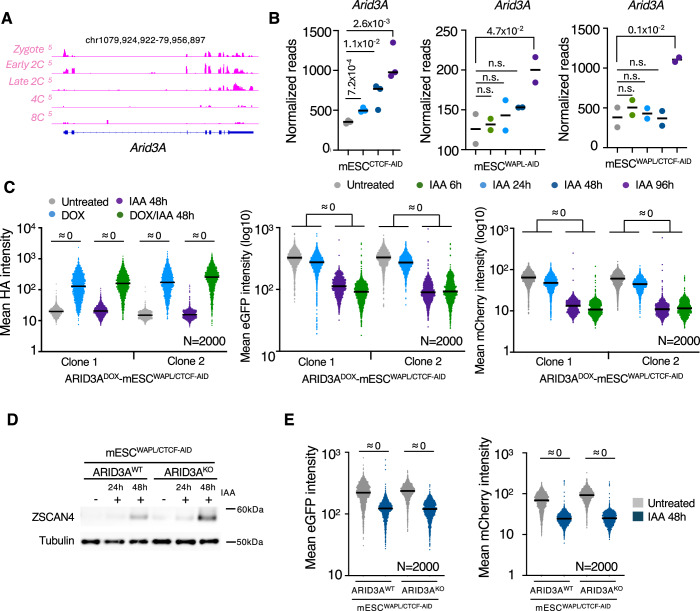
Supporting Fig. 3. ARID3A levels determine 2CLC conversion efficiency. (**A**) Genome browser tracks showing RNA-seq RPKM read count at *Arid3A* in samples obtained from Deng et al, 2014. (**B**) Plots showing normalized read counts obtained from RNA-seq datasets corresponding to untreated or IAA-treated for the indicated times mESC^CTCF-AID^, mESC^WAPL-AID^ and mESC^WAPL/CTCF-AID^ samples. Data was obtained from Nora et al, 2017; Liu et al, 2021, 2025. *P* values are shown from one-tailed unpaired *t* tests. (**C**) High-throughput imaging quantification of HA (left panel) eGFP (center panel) and mCherry (right panel) mean nuclear intensity in ARID3A^DOX^-mESC^WAPL/CTCF-AID^ that were untreated or treated with DOX and/or IAA for 48 h. Center lines indicate mean values. *P* values are shown from one-tailed unpaired *t* tests. (**D**) Western blot analyses of ZSCAN4 performed in lysates from wild-type or ARID3A-deficient mESC^WAPL/CTCF-AID^ untreated or treated with IAA for 48 h. TUBULIN levels are shown as a loading control. (**E**) High-throughput imaging quantification of eGFP (left panel) and mCherry (right panel) mean nuclear intensity in wild-type or ARID3A-deficient mESC^WAPL/CTCF-AID^ that were untreated or treated with IAA for 48 h. Center lines indicate mean values. *P* values are shown from one-tailed unpaired *t* tests. Data information: For (**C**–**E**), two independent experiments were performed but only one representative is shown. Source data are available online for this figure.

**Figure 3:**
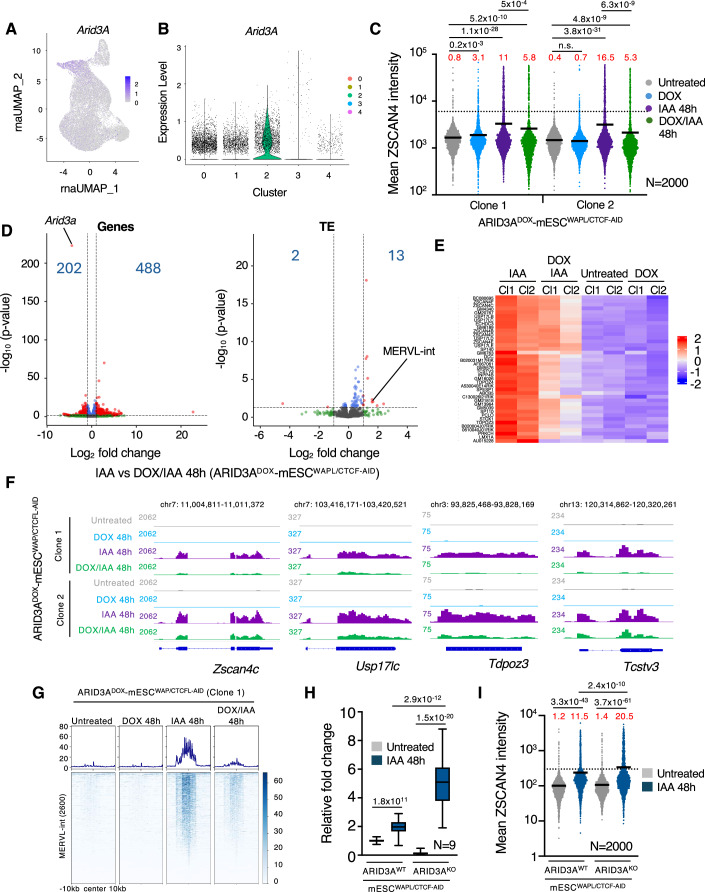
ARID3A levels determine 2CLC conversion efficiency upon CTCF/WAPL depletion. (**A**) UMAP visualization showing expression levels of *Arid3A* in samples from Fig. 2C. (**B**) Plot showing expression levels of *Arid3A* in individual cells from identified clusters (Fig. 2C). (Cluster 0, *n* = 10123; Cluster 1, *n* = 6203; Cluster 2, *n* = 2697; Cluster 3, *n* = 1960; Cluster 4, *n* = 1444). (**C**) High-throughput imaging quantification of ZSCAN4 mean nuclear intensity in two independent ARID3A^DOX^ mESC^WAPL/CTCF-AID^ clonal lines that were untreated or treated with DOX and/or IAA for 48 h. Center lines indicate mean values. *N* = 2000. Numbers in red show the percentage of ZSCAN4^+^ cells above the arbitrary threshold. Three independent experiments were performed but only one representative is shown. *P* values are shown from one-tailed unpaired *t* tests. (**D**) Volcano plots showing differentially expressed genes (left panel) and TE (right panel) in ARID3A^DOX^ mESC^WAPL/CTCF-AID^ that were treated with IAA or DOX/IAA for 48 h. Two independent cell clones were used in this analysis. The number of differentially expressed genes and TE families are indicated. A Wald test was used to determine statistical significance. Thresholds are Log2 fold change>1 and *P* value < 0.01. (**E**) Heatmap generated from RNA-seq data from (**D**) showing the expression level of the top 40 most upregulated genes upon DUX expression based on Cutoff Log2 > 1, *P* value < 0.05 (dataset from Hendrickson et al, 2017). (**F**) Genome browser tracks showing RNA-seq RPKM read count at the indicated genes in samples obtained from ARID3A^DOX^ mESC^WAPL/CTCF-AID^ that were untreated or treated with DOX and/or IAA for 48 h. (**G**) Read density plots (RPGC) and corresponding heatmaps showing RNA-seq RPKM read count at the set of MERVL-int elements in one representative ARID3A^DOX^ mESC^WAPL/CTCF-AID^ clone that were untreated or treated with DOX and/or IAA for 48 h. (**H**) Real time-PCR analysis shown in a box and whisker plot showing the relative fold change (log2) expression of the same nine 2C-associated transcripts as in Fig. 1C in wild-type or *Arid3A* -deficient mESC^WAPL/CTCF-AID^ that were untreated or treated with IAA for 48 h. *Gapdh* expression was used to normalize. For the box plot, center line indicates the median, box extends from the 25th to 75th percentiles and whiskers show minimum to maximum values. (**I**) High-throughput imaging quantification of ZSCAN4 mean nuclear intensity in wild-type or ARID3A-deficient mESC^WAPL/CTCF-AID^ that were untreated or treated with IAA for 48 h. Center lines indicate mean values. Numbers in red show the percentage of ZSCAN4^+^ cells above the arbitrary threshold. Similar results were obtained in independent clones from the same genotype. Data information: For (**H**, **I**), two independent experiments were performed but only one representative is shown. Source data are available online for this figure.

To gain further mechanistic insights into the 2CLC conversion mediated by CTCF/WAPL depletion, we focused on the AT-rich interaction domain 3 A (ARID3A) transcription factor, whose motif is enriched in regions that are differentially accessible in cells from cluster 4 (Figs. 2L and EV2F) suggesting a role during 2 CLC conversion. ARID3A has been implicated in B lymphocyte development and autoimmunity as well as in regulating trophectoderm lineage commitment and differentiation (Rhee et al, 2014; Guo et al, 2023). However, whether ARID3A participates in ZGA is unknown. Interestingly, ARID3A showed elevated levels in zygotes and early 2C-stage embryos suggesting maternal inheritance (Fig. EV3A). We first examined *Arid3A* expression levels in WAPL, CTCF and WAPL/CTCF-depleted mESC and observed increased levels over time (Fig. EV3B). Moreover, we observed that *Arid3A* expression is specifically enriched in cells from cluster 2 and is decreased during the transition to 2CLC from cluster 4 (Fig. 3A,B). This observation suggests that ARID3A might be detrimental for 2CLC conversion. To explore this possibility, we generated DOX-inducible ARID3A expressing cells in a WAPL/CTCF-degron background (ARID3A^DOX^ mESC^WAPL/CTCF-AID^) and examined the effects of ARID3A overexpression in 2CLC conversion. Remarkably, ARID3A expression reduced the percentage of ZSCAN4 positive cells in IAA-treated cells (Figs. 3C and EV3C). We next performed RNA-seq analyses in untreated, IAA and DOX/IAA-treated ARID3A^DOX^ mESC^WAPL/CTCF-AID^ for 48 h. To examine the transcriptional changes induced by ARID3A overexpression, we focused our analysis on comparing IAA and DOX/IAA-treated cells (Dataset EV5). We identified hundreds of differentially expressed genes between these conditions (Fig. 3D). Importantly, we observed that ARID3A limited the induction of the 2C-transcriptional program upon IAA treatment, including MERVL elements (Fig. 3E–G). Conversely, ARID3A inactivation further promoted 2 CLC conversion and enhanced the induction of the 2C-transcriptional program following CTCF/WAPL depletion (Figs. 3H,I and EV3D,E). In summary, our data revealed an unexpected role for ARID3A in limiting the extent of 2CLC conversion.

### CTCF or WAPL depletion in hESC does not trigger the expression of early embryonic transcripts

**Figure 4:**
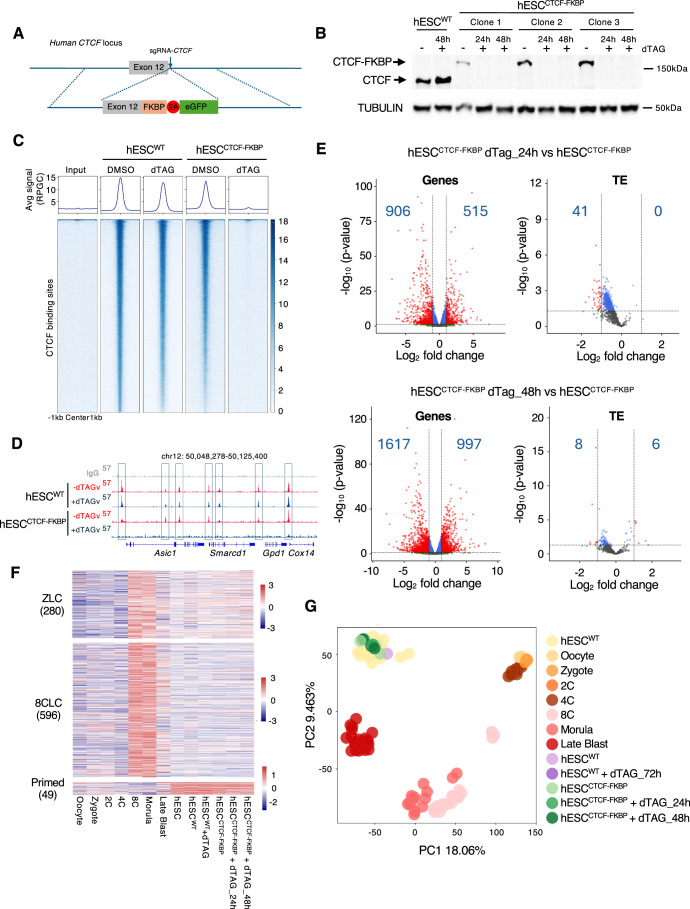
Loss of CTCF in hESC does not trigger expression of earlier transcriptional programs. (**A**) Schematic representation of the targeting strategy for tagging the human CTCF allele with an FKBP-2A-eGFP cassette. (**B**) Western blot analysis of CTCF performed in lysates from different hESC^CTCF-FKBP^ cell clones that were untreated or treated with dTAG for 24 or 48 h. hESC^WT^ were included to show the increased size of the tagged CTCF protein. TUBULIN levels are shown as a loading control. (**C**) Heatmaps derived from Cut&Run experiments in hESC^WT^ and a representative hESC^CTCF-FKBP^ clone that were untreated or treated with dTAG for 24 h, showing CTCF occupancy at the CTCF binding sites identified in hESC^WT^. (**D**) Genome browser tracks showing CTCF occupancy at the indicated genomic location in hESC^WT^ and a representative hESC^CTCF-FKBP^ cell clone that were untreated or treated with dTAG for 24 h. (**E**) Volcano plots showing differentially expressed genes (left panels) and TE (right panels) in hESC^CTCF-FKBP^ that were untreated or treated with dTAG for 24 h (upper panels) or 48 h (lower panels). Three independent cell clones were used in this analysis. The number of differentially expressed genes and TE families are indicated. A Wald test was used to determine statistical significance. Thresholds are Log2 fold change>1 and *P* value < 0.01. (**F**) Heatmap generated from the RNA-seq data shown in (**E**), including hESC^WT^ that were untreated or treated with dTAG for 48 h as well as published RNA-seq datasets from single cells derived from different stages of human embryonic development. Heatmaps show subsets of genes enriched in ZLC (280 genes), 8CLC (596 genes) and in primed pluripotent cells (49 genes). (**G**) PCA plot including the same samples as in (**F**). Source data are available online for this figure.

We next investigated whether the expression of ZGA-associated genes due to alterations in higher-order chromatin organization is conserved across mammals. Therefore, we generated a degron system in human ESC (hESC) by targeting an FKBP-2A-eGFP cassette to the endogenous *CTCF* loci (hESC^CTCF-FKBP^ hereafter; Fig. 4A). hESC^CTCF-FKBP^ did not show any morphological abnormality or growth disadvantage compared to hESC^WT^ (Appendix Fig. S5A). By performing Cut&Run experiments to examine CTCF binding, we observed that over 87% of CTCF peaks identified in hESC^WT^ overlap with those from published ChIP-seq datasets (Appendix Fig. S5B and Dataset EV6) (Dixon et al, 2015). Moreover, over 85% of CTCF peaks found in hESC^WT^ overlap with those of hESC^CTCF-FKBP^ tagged cells (Appendix Fig. S5B,C). These data suggest that CTCF binding to chromatin is not majorly affected by the FKBP tagging. dTAG treatment efficiently degrades the CTCF-FKBP protein in hESC^CTCF-FKBP^ and it is undetected in chromatin (Fig. 4B–D). Interestingly, CTCF depletion in hESC leads to massive cell death 48 h after dTAG addition (Appendix Fig. S5A).

To examine the transcriptional consequences following CTCF depletion, we performed RNA-seq analysis on three independent hESC^CTCF-FKBP^ cell lines, either untreated and dTAG-treated for 24 or 48 h. Volcano plots showed that 24 h after CTCF depletion, the number of downregulated genes (906) almost double the number of upregulated genes (515) (Fig. 4E; Dataset EV7). This trend is maintained after 48 h of dTAG treatment. Moreover, up to 41 families of transposable elements are downregulated 24 h after CTCF depletion, but this change seems to disappear 24 h later (Fig. 4E; Dataset EV7). Gene Ontology term analysis showed enrichment in neural development, cell adhesion and motility and developmental growth for downregulated genes (Dataset EV8). We also found a similar percentage of genes differentially expressed independent of their proximity to a CTCF-bound site (Appendix Fig. S5D). Furthermore, we observed both up- and downregulated genes with CTCF-bound sites near their promoters (Appendix Fig. S5E,F). Interestingly, we detected upregulation of multiple differentiation genes in dTAG-treated hESC^CTCF-FKBP^ cell lines, including *LEFTY1* and *EOMES*, suggesting the relevance of CTCF in preserving hESC identity (Fig. Appendix Fig. 5G).

We next determined whether CTCF depletion in human cells promoted the expression of early embryonic transcripts. For this, we used a subset of genes differentially expressed in two recently reported human cell models expressing ZGA genes, zygotic-like cells (ZLC, 280 genes) and 8C-like cells (8CLC, 596 genes) (Yan et al, 2013; Li et al, 2024). We also used a set of genes differentially expressed in primed hESC to confirm changes in cell identity (49 genes) (Yan et al, 2013; Li et al, 2024). Using these gene lists, we generated a heatmap using our RNA-seq data from untreated or dTAG-treated hESC^WT^ and hESC^CTCF-FKBP^ as well as published RNA-seq data from single cells derived from different human embryonic stages from oocyte to late blastocyst (Fig. 4F,G) (Yan et al, 2013). Surprisingly, we did not detect expression of ZGA-associated genes in CTCF-depleted hESC. Furthermore, CTCF-depleted hESC clustered together with their corresponding controls rather than with early stages of human development. We thus conclude that CTCF depletion is not sufficient to induce expression of early embryonic transcripts in hESC.

**Figure EV4:**
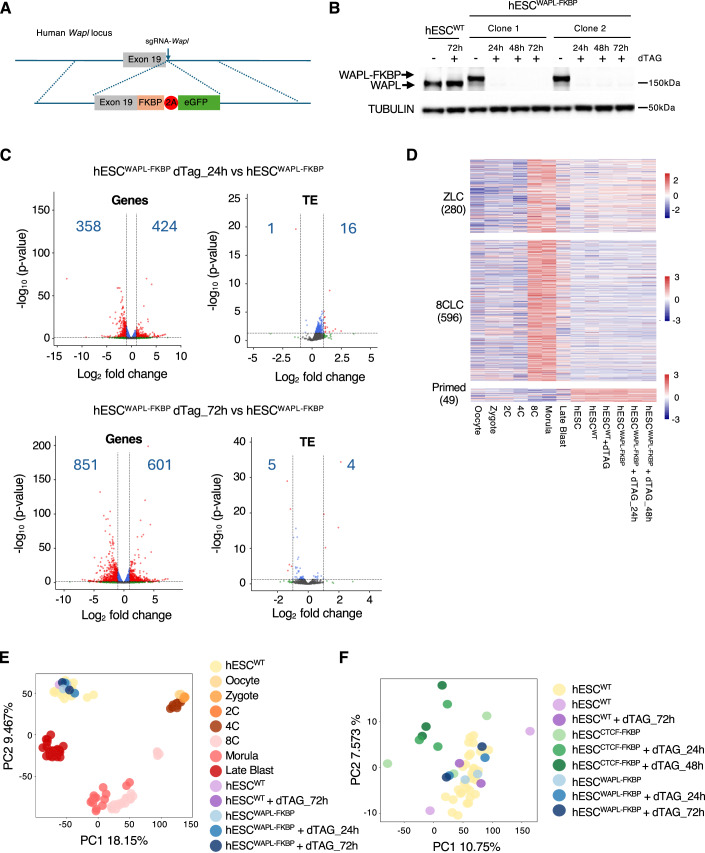
Supporting Fig. 4. Loss of WAPL in hESC does not trigger expression of earlier transcriptional programs. (**A**) Schematic representation of the targeting strategy for tagging the human WAPL allele with an FKBP-2A-eGFP cassette. (**B**) Western blot analysis of WAPL performed in lysates from different hESC^WAPL-FKBP^ cell clones that were untreated or treated with dTAG for 24, 48 and 72 h. hESC^WT^ were included to show the increased size of the tagged WAPL protein. TUBULIN levels are shown as a loading control. Two independent experiments were performed but only one representative is shown. (**C**) Volcano plots showing genes (left panels) and TE (right panels) that are differentially expressed in hESC^WAPL-FKBP^ that were untreated or treated with dTAG for 24 (upper panels) or 72 h (lower panels). Three independent cell clones were used in this analysis. Indicated are the number of differentially expressed genes and TE families. A Wald test was used to determine statistical significance. Thresholds are Log2 fold change>1 and *P* value < 0.01. (**D**) Heatmaps generated from the RNA-seq data shown in (**C**), including hESC^WT^ that were untreated or treated with dTAG for 48 h as well as published RNA-seq datasets from single cells derived from different stages of human embryonic development. Heatmaps show subsets of genes enriched in ZLC (280 genes), 8CLC (596 genes) and in primed pluripotent cells (49 genes). (**E**) PCA plot including the same samples as in (**D**). (**F**) PCA plot including all hESC^WT^, hESC^WAPL-FKBP^ hESC^CTCF-FKBP^ cell lines that were untreated and treated with dTAG for the indicated times. Source data are available online for this figure.

To extend these observations, we tagged endogenous *WAPL* with the FKBP degron construct in hESC (hESC^WAPL-FKBP^; Fig. EV4A,B) and examined gene and transposable elements expression (Fig. EV4C and Datasets EV9 and 10). Similar to our observations in CTCF-depleted hESC, we did not detect upregulation of the gene subsets characterizing ZLC and 8CLC, and WAPL-depleted hESC still clustered with untreated cells (Fig. EV4D–F). In summary, the reactivation of transcriptional programs from early developmental stages following depletion of proteins regulating higher-order chromatin organization does not seem to be conserved in hESC.

### 2CLC conversion induced by the loss of CTCF is DUX-dependent

**Figure 5:**
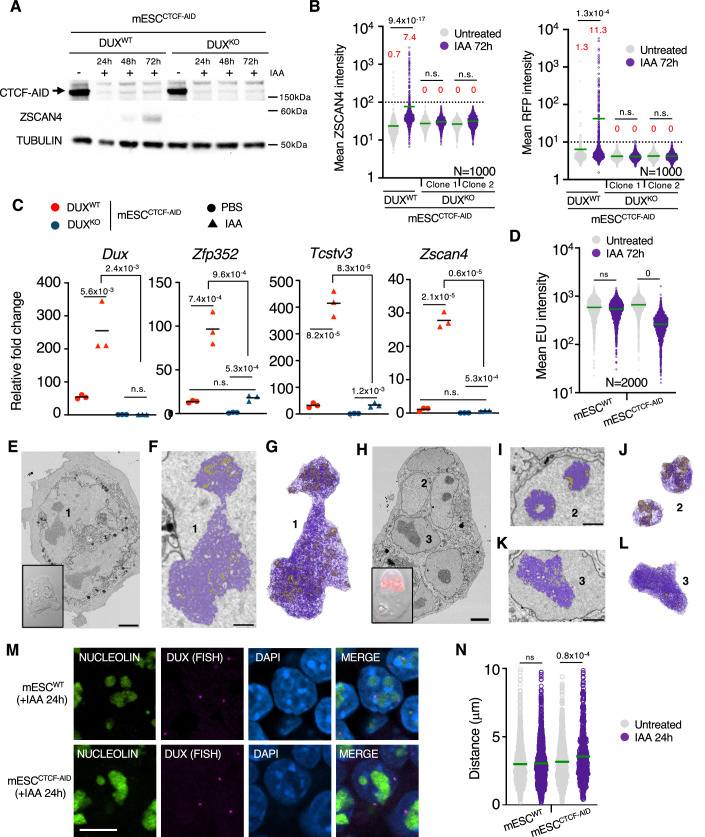
2CLC conversion upon CTCF loss depends on endogenous *Dux* reactivation. (**A**) Western blot analysis of CTCF and ZSCAN4 performed in lysates from wild-type and DUX-deficient mESC^CTCF-AID^ clones untreated or IAA-treated for 24, 48 or 72 h. TUBULIN levels are shown as a loading control. (**B**) High-throughput imaging quantification of ZSCAN4 (left panel) and RFP derived from a MERVL-driven reporter (right panel) mean nuclear intensity in wild-type and DUX-deficient mESC^CTCF-AID^ clones that were untreated or treated with IAA for 72 h. Center lines indicate mean values. Numbers in red show the percentage of ZSCAN4^+^ cells above the arbitrary threshold. Three independent experiments were performed but only one representative is shown. (**C**) Real time-PCR analysis showing the relative fold change expression of the indicated genes in wild-type and DUX-deficient mESC^CTCF-AID^ clones that were untreated or treated with IAA for 72 h. *Gapdh* expression was used to normalize. Two independent experiments with reactions in triplicate were performed but only one representative is shown. (**D**) High-throughput imaging quantification of EU mean nuclear intensity in wild-type and DUX-deficient mESC^CTCF-AID^ clones that were untreated or treated with IAA for 72 h. Center lines indicate mean values. Three independent experiments were performed but only one representative is shown. (**E**–**L**) Selected 2D image planes from volume electron microscopy reconstructions from mESC^CTCF-AID^ treated with IAA for 48 h (**E**, **H**), and high-resolution images of corresponding numbered nuclei with segmented granular components (purple) and dense fibrillar component (yellow) of nucleoli shown in 2D (**F**, **I**, **K**) or 3D (**G**, **J**, **L**). Insets in (**E**, **H**) show correlative fluorescence images of mESC clusters overlayed with RFP signal derived from a MERVL-driven reporter to indicate 2CLC. Different z-slices are shown in (**H**, **I**). Scale bar (**E**, **H**): 4 μm; (**F**, **I**, **K**): 1 μm. Numbers on images indicate the representative specific nuclei, and its corresponding nucleoli shown. (**M**) Confocal microscopy images showing immunostaining for nucleolin and DNA FISH signal to detect DUX loci in representative mESC^WT^ and mESC^CTCF-AID^ treated with IAA for 24 h. Scale bar: 10 μm. (**N**) Plot showing the distance from each individual *Dux* FISH spot to the center of the nucleoli. At least 500 independent loci are plotted per condition. Data information: For (**B**–**D**, **N**), *P* values are shown from one-tailed unpaired *t* tests. Source data are available online for this figure.

Finally, we explored the molecular mechanism underlying the 2CLC reprogramming phenotype. Due to the essential role of DUX during spontaneous reprogramming to 2CLC (De Iaco et al, 2017), we first examined the relevance of DUX during 2CLC conversion following CTCF depletion. We introduced an AID-eGFP sequence at the C-terminus of the endogenous *Ctcf* locus (mESC^CTCF-AID^) in wild-type or *Dux*-knockout mESC (De Iaco et al, 2017; Nora et al, 2017). Addition of IAA promoted rapid CTCF degradation in both cell lines (Fig. 5A). Importantly, although DUX expression increases and 2 CLC conversion is efficiently induced upon CTCF depletion, this reprogramming is completely abolished in *Dux*-deficient mESC^CTCF-AID^, as evidenced by the lack of expression of 2C-associated markers, including MERVL elements (Fig. 5A–C; Appendix Fig. S6A). Therefore, we concluded that 2CLC conversion upon CTCF depletion is DUX-dependent in mESC.

We next sought to determine how CTCF depletion leads to DUX activation. Recent studies have shown that inhibition of ribosomal RNA (rRNA) biogenesis and disruption of nucleolar integrity can promote 2CLC transition (Xie et al, 2022; Yu et al, 2021; Sun et al, 2022). Phase-separated nucleolar integrity is essential to maintain the 3D structure of the nucleolus and the perinucleolar heterochromatin (PNH). Alterations in nucleolar integrity changes the epigenetic and 3D state of the PNH in mESC, leading to the release and activation of the *Dux* locus, which is inactive and localized to the repressive PNH region (Xie et al, 2022; Yu et al, 2021; Sun et al, 2022). Supporting these observations in mESC, exit from totipotency in mouse embryos correlated with increased rRNA expression, nucleolus maturation, PNH formation, and *Dux* silencing through its recruitment to the nucleolar periphery (Xie et al, 2022; Yu et al, 2021; Sun et al, 2022). We hypothesized that because the nucleolus requires a specific 3D organization, alterations in higher-order chromatin organization could impact nucleolar integrity. Consistent with this hypothesis, cohesin mutations in yeast are associated with defects in ribosome biogenesis and nucleolar integrity (Bose et al, 2012; Harris et al, 2014). To assess the functionality of the nucleolus in CTCF-depleted mESC, we first examined the overall transcriptional level by detecting 5-ethynyl uridine (EU) incorporation in untreated or IAA-treated mESC^CTCF-AID^. We detected a dramatic decrease in overall transcription levels in all CTCF-depleted cells (Fig. 5D). To visualize potential changes to nucleolar structure, we performed focused ion beam scanning electron microscopy (FIB-SEM) to enable a high-resolution 3D reconstruction in untreated and IAA-treated mESC^CTCF-AID^ for 48 h (Fig. 5E–L; Movies EV1 and 2). To distinguish between the effects of CTCF depletion and 2CLC reprogramming on the nucleolus, we used the MERVL-driven reporter expressing RFP to identify pluripotent mESC (RFP-) and 2CLC (RFP + ) from the population of CTCF-depleted cells. Nucleolus reconstructions revealed a tendency to a smaller overall volume, including granular content, and the presence of nucleolar fragmentation in CTCF-depleted cells (Appendix Fig. S6B,C). Transmission electron tomography also showed a consistent decrease in the size of fibrillar centers, sites of rRNA transcription (Appendix Fig. S6D). Combined, these observations suggest that CTCF depletion alters nucleolar integrity and might affect PNH-dependent *Dux* silencing. We then performed DNA fluorescence in situ hybridization (FISH) analyses in untreated or IAA-treated mESC^WT^ and mESC^CTCF-AID^ to determine the location of *Dux* loci in relation to the nucleolus position. We found that *Dux* loci are consistently relocated further from the PNH in CTCF-depleted cells compared to untreated mESC^WT^ and mESC^CTCF-AID^ or IAA-treated mESC^WT^ (Fig. 5M,N). These results indicate that CTCF depletion, leads to the release of the *Dux* loci from repressive PNH, favoring DUX expression and downstream induction of 2C-associated transcripts.

Importantly, global transcription levels were also severely reduced in WAPL depleted cells, suggesting that nucleolar integrity and PNH-directed *Dux* retention might also be compromised in this cellular setting (Appendix Fig. S7A). Accordingly, dTAG-treated DUX-deficient mESC^WAPL-FKBP^ do not show any expression of 2C-associated markers confirming that DUX is also essential for WAPL-mediated 2CLC conversion (Appendix Fig. S7B–D). These findings demonstrate that CTCF or WAPL depletion promotes efficient 2CLC conversion in a DUX-dependent manner.

### DPPA2 is required for *Dux* expression following combined CTCF/WAPL loss

**Figure EV5:**
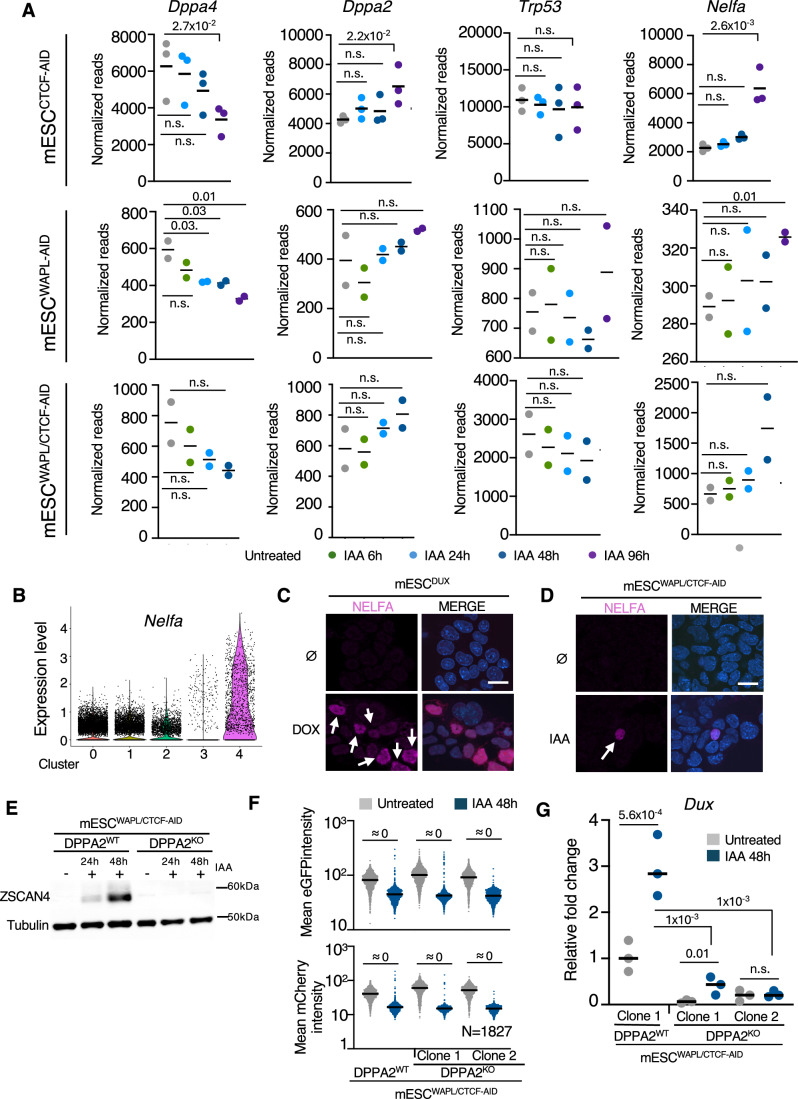
Supporting Fig. 6. DPPA2 is essential for 2CLC conversion upon CTCF/WAPL depletion. (**A**) Plots showing normalized read counts obtained from RNA-seq datasets corresponding to untreated or IAA-treated for the indicated times mESC^CTCF-AID^, mESC^WAPL-AID^ and mESC^WAPL/CTCF-AID^ samples. Data was obtained from Nora et al, 2017; Liu et al, 2021 and Liu et al, 2025. (**B**) UMAP visualization showing expression levels of *Nelfa* in samples from Fig. 2C. (**C**, **D**) Representative confocal microscopy images showing representative NELFA staining in mESC^DUX^ that were either untreated or DOX-treated for 16 h (**C**) and mESC^WAPL/CTCF-AID^ that were either untreated or IAA treated for 48 h (**D**). Arrows indicate NELFA positive cells. DAPI was used to visualize nuclei. Scale bar: 10 μm. (**E**) Western blot analysis of ZSCAN4 performed in lysates from untreated or IAA-treated wild-type and DPPA2-deficient mESC^CTCF-AID^ clones. TUBULIN levels are shown as a loading control. (**F**) High-throughput imaging quantification of eGFP (upper panel) and mCherry (lower panel) mean nuclear intensity in wild-type and *Dppa2*-deficient mESC^WAPL/CTCF-AID^ that were untreated or treated with IAA for 48 h. Center lines indicate mean values. Two independent experiments were performed but only one representative is shown. For (**A**, **C**, **E**, **F**), *P* values are shown from one-tailed unpaired *t* tests. (**G**) Plot generated with real time-PCR analysis data showing the relative fold change expression of *Dux* in wild-type and *Dppa2*-deficient mESC^WAPL/CTCF-AID^ that were untreated or treated with IAA for 48 h. *Gapdh* expression was used to normalize. Center lines indicate mean values. Two independent experiments were performed but only one representative experiment is shown. Source data are available online for this figure.

**Figure 6:**
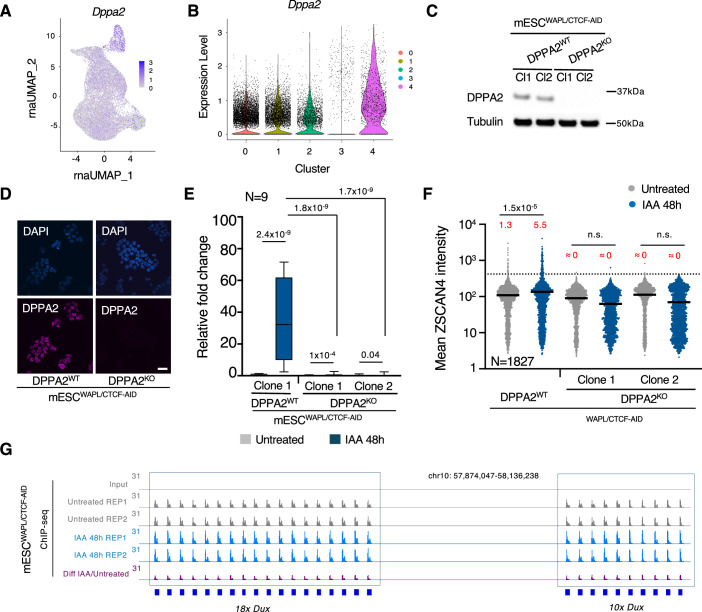
DPPA2 is required for 2CLC conversion upon CTCF/WAPL depletion. (**A**) UMAP visualization showing expression levels of *Dppa2* in samples from Fig. 2C. (**B**) Plot showing expression levels of *Dppa2* in individual cells from identified clusters (Fig. 2C). (Cluster 0, *n* = 10123; Cluster 1, *n* = 6203; Cluster 2, *n* = 2697; Cluster 3, *n* = 1960; Cluster 4, *n* = 1444). (**C**) Western blot analysis of DPPA2 performed in lysates from wild-type and DPPA2-deficient mESC^WAPL/CTCF-AID^ clones. TUBULIN levels are shown as a loading control. (**D**) Confocal microscopy images showing representative DPPA2 staining in wild-type and *Dppa2*-deficient mESC^WAPL/CTCF-AID^. DAPI was used to visualize nuclei. Scale bar: 20 μm. (**E**) Real time-PCR analysis shown in a box and whisker plot showing the relative fold change expression of the same nine 2C-associated transcripts as in Fig. 1C in wild-type and *Dppa2*-deficient mESC^WAPL/CTCF-AID^ clones that were untreated or treated with IAA for 48 h. *Gapdh* expression was used to normalize. For the box plot, center line indicates the median, box extends from the 25th to 75th percentiles and whiskers show minimum to maximum values. Two independent experiments were performed but only one representative experiment is shown. (**F**) High-throughput imaging quantification of ZSCAN4 mean nuclear intensity in wild-type and DPPA2-deficient mESC^WAPL/CTCF-AID^ clones that were untreated or treated with IAA for 48 h. Center lines indicate mean values. Two independent experiments were performed but only one representative is shown. (**G**) Genome browser tracks showing ChIP-seq reads at the genomic region containing the *Dux* cluster of genes generated from mESC^CTCF/WAPL-AID^ lines that were untreated and treated with IAA for 48 h. The lower track shows the subtraction of the averaged signal between untreated and IAA-treated cells. Boxed are the two main regions containing 18 and 10 *Dux* gene copies. Two replicates per condition are shown. Data information: For (**E**, **F**), *P* values are shown from one-tailed unpaired *t* tests. Source data are available online for this figure.

We hypothesized that the release of the *Dux* loci from the repressive PNH could facilitate binding accessibility to transcriptional regulators. Expression of *Dux* is directly regulated by the transcription factors TRP53, DPPA2/4 and the negative elongation factor complex member A (NELFA) (Grow et al, 2021; Hu et al, 2020; Eckersley-Maslin et al, 2019). Alterations in chromatin organization could alter the expression of these factors, directly or indirectly, leading to *Dux* expression and 2CLC conversion. Interestingly, while *Dppa4* is downregulated and *Trp53* remains unchanged over time, *Dppa2* and *Nelfa* show a slight tendency to become upregulated over time in all genetic conditions (Fig. EV5A). Thus, these transcription factors could potentially participate in promoting *Dux* expression and/or influence successful 2CLC reprogramming. Importantly, the use of bulk analyses on heterogeneous populations undergoing 2CLC conversion could obscure important expression changes in different subsets of cells. Thus, we used our multiome dataset (Fig. 2) to examine the transcriptional dynamics of *Dppa2* and *Nelfa* following CTCF/WAPL depletion. Although *Dppa2* and *Nelfa* expression is enriched in cells from cluster 4, they also seem expressed in cells from all other clusters (Figs. 2F, 6A,B and EV5B). However, while DPPA2 protein expression is ubiquitous in untreated cells, NELFA protein expression is negligible and can only be detected following CTCF/WAPL-depletion or DUX expression (Figs. 6C,D and EV5C,D). Furthermore, recent work has suggested that NELFA could be a marker rather than a driver of the 2CLC state (Grow et al, 2021). Therefore, we decided to focus on the role of DPPA2 as it is already expressed in untreated mESC^WAPL/CTCF-AID^ and could influence 2CLC reprogramming at different steps during the process. Moreover, *Dppa2*-deficient mESC do not show spontaneous reprogramming to 2CLC (Eckersley-Maslin et al, 2019; De Iaco et al, 2019).

To determine the relevance of DPPA2 in CTCF/WAPL-mediated 2CLC conversion, we generated *Dppa2*-deficient mESC^CTCF/WAPL-AID^ (Fig. 6C,D). Remarkably, DPPA2 deficiency completely abrogated the generation of ZSCAN4 positive cells as well as the expression of 2CLC-associated transcripts in IAA-treated mESC^WAPL/CTCF-AID^ (Figs. 6E,F and  EV5E,F). Furthermore, the lack of 2CLC conversion seems due to the inability to promote meaningful levels of *Dux* expression in the absence of DPPA2 (Fig. EV5G). Interestingly, we observed increased DPPA2 binding at all *Dux* gene copies from the cluster following CTCF/WAPL depletion suggesting a direct role of DPPA2 in *Dux* expression (Fig. 6G). In summary, our results demonstrate the essential role of DPPA2 in promoting *Dux* expression and 2 CLC conversion upon CTCF/WAPL loss.

## Discussion

Higher-order chromatin structure plays a key role in regulating gene expression. Whether chromatin dynamics affect the expression of transcriptional programs impacting cell transition states during development remains to be determined. In this work, we demonstrate the role of proteins involved in chromatin organization in controlling the expression of the totipotency-associated transcriptional program. Depletion of CTCF or WAPL promoted efficient 2CLC conversion in pluripotent mESC. Furthermore, the combined loss of CTCF and WAPL had an additive effect on 2CLC conversion efficiency. Interestingly, the loss of CTCF in mESC leads to TAD disruption (Nora et al, 2017), whereas WAPL depletion results in altered cohesin turnover and induce mESC differentiation (Liu et al, 2021). These observations suggested that CTCF and WAPL perform distinct but complementary functions during 2CLC reprogramming and might explain the additive effect of co-depletion.

Single-cell RNA-seq/ATAC-seq analyses revealed that the combined loss of CTCF and WAPL led over time to a general shift toward a 2CLC transcriptional state (cluster 4). In addition, we observed increased chromatin accessibility in regions near 2C-associated genes and MERVL repeats prior to their activation. Interestingly, acute loss of either CTCF or WAPL did not induce major changes in steady-state RNA levels 24 h after IAA treatment (Nora et al, 2017; Liu et al, 2021). We also observed that mESC largely remain in the same transcriptomic space (cluster 0) following 24 h of CTCF/WAPL depletion. However, single-cell ATAC-seq data revealed that CTCF/WAPL depleted cells are found in a different chromatin state only 24 h after IAA treatment compared to untreated cells. Similarly, acute CTCF depletion led to a genome-wide rewiring in chromatin accessibility (Xu et al, 2021). These observations suggested that overall changes in gene expression due to the loss of CTCF or CTCF/WAPL follow alterations in chromatin accessibility. Independent of these results, we detected differential nascent RNA expression of 2C-associated genes following acute WAPL depletion as early as 6 h, suggesting that the absence of WAPL influences their deregulation. This could be due to the existence of a subpopulation of cells already biased and transcriptionally closer to a 2CLC state (cells in cluster 2 in untreated ESC^WAPL/CTCF-AID^). Thus, we propose that CTCF/WAPL depletion in mESC favors changes in chromatin accessibility in an asynchronous manner ultimately leading to DUX induction and a sustained and stable expression of the 2C-associated transcriptional program. Furthermore, we identified the transcription factor ARID3A as a regulator of the 2CLC state. Indeed, ARID3A levels determined 2CLC conversion efficiency levels in vitro. Because ARID3A seems to be maternally inherited, future studies will need to determine its relevance during ZGA in vivo.

Our results revealed that 2CLC conversion following CTCF or WAPL depletion requires *Dux* expression. We observed that CTCF depletion leads to a global decrease in the levels of transcription, correlating with nucleolar structural alterations. Interestingly, the use of specific treatments to disrupt nucleolar integrity such as Pol I inhibitors or 1, 6-hexanediol (HDL), which affect rRNA biogenesis and disrupt liquid-like condensates, respectively, promote 2CLC conversion (Yu et al, 2021; Xie et al, 2022). These treatments facilitate the release of *Dux* from PNH and its subsequent activation (Yu et al, 2021; Xie et al, 2022). This activation seems stochastic as 2CLC conversion following Pol I inhibitors, HDL treatments or CTCF or WAPL depletion is not fully penetrant. Thus, *Dux* release from the PNH is not sufficient to trigger its expression, and additional factors likely determine its reactivation. In this regard, we identified the relevance of DPPA2 mediating 2CLC conversion upon CTCF/WAPL depletion, as *Dppa2*-deficient cells are unable to induce 2C-associated transcripts. Interestingly, although DPPA2 is expressed in untreated pluripotent mESC and has been shown to directly bind the *Dux* promoter but not to other 2C-associated genes (Fig. 6G; Eckersley-Maslin et al, 2019), it seems unable to activate *Dux* expression in most cells. Importantly, CTCF/WAPL depletion leads to increased DPPA2 occupancy and chromatin accessibility at all *Dux* gene copies (Figs. 6G and EV2A). DPPA2 lacks any known enzymatic activity and has been suggested to be an epigenetic priming factor, as its binding could be required for future activation upon specific signals or transcription factors (Eckersley-Maslin, 2020). Future studies will be needed to determine the precise role of DPPA2 enrichment at the *Dux* gene cluster. Nevertheless, our findings suggest a model where the loss of CTCF/WAPL leads to the release of the *Dux* loci from the repressive PNH environment, enabling further DPPA2 enrichment at the *Dux* gene copies and facilitating *Dux* expression in a DPPA2-dependent manner to promote 2CLC conversion.

Finally, we found that CTCF or WAPL depletion in hESC did not induce the expression of ZGA-associated transcripts, suggesting a lack of conservation in mammals. However, hESC reside in a state of primed pluripotency resembling cells from the pre-implantation epiblast compared to naïve pluripotent mESC, which are more similar to earlier stages of development (Manor et al, 2015). This is relevant because 8 CLCs, a subpopulation of cells found endogenously in hESC cultures and transcriptionally resembling blastomeres from the 8C-stage human embryo, can only be found within cultures of naïve hESC but not in primed or differentiated cells (Taubenschmid-Stowers et al, 2022). Indeed, we could not detect the expression of 2C-associated markers in CTCF/WAPL-depleted mNSC, despite efficiently inducing 2CLC conversion in mESC. Alternatively, expression of species-specific factors might account for the differences observed. In summary, our data suggest that cell commitment and differentiation during development impose barriers (e.g. epigenetic or associated to higher-order chromatin structures) to prevent the activation of the totipotent transcriptional program in cells with restricted developmental potential. Furthermore, the ability to acquire totipotent-like features might be restricted as early as during the naïve-to-primed pluripotency transition. In fact, this transition is characterized by an extensive 3D chromatin remodeling that could contribute to imposing a totipotent-like conversion barrier (Novo et al, 2018; Battle et al, 2019; Joshi et al, 2015). Future studies will need to address whether depletion of CTCF or WAPL in naïve hESC or in primed mouse epiblast cells could promote the formation of 8CLC or 2CLC, respectively. In summary, our work revealed the role of proteins regulating chromatin organization controlling the expression of totipotency-associated transcriptional programs.

## Methods

**Table:** Reagents and tools table

Reagent/resource	Reference or source	Identifier or catalog number
**Experimental models**		
R1 wild-type mESC	This study	N/A
G4 wild-type mESC	This study	N/A
E14 wild type mESC	This study	N/A
mESC^CTCF-AID^	Nora et al, 2017	EN52.9.1
Doxycycline-inducible DUX mESC	Olbrich et al, 2021	N/A
mESC^WAPL-FKBP^	This study	N/A
Doxycycline-inducible ARID3A mESC	This study	N/A
DUX-deficient mESC	De Iaco et al, 2017	N/A
DUX-deficient mESC^WAPL-FKBP^	This study	N/A
DUX-deficient mESC^CTCF-AID^	This study	N/A
hESC^WAPL-FKBP^	This study	N/A
hESC^CTCF-FKBP^	This study	N/A
DPPA2-deficient mESC^WAPL/CTCF-AID^	This study	N/A
ARID3A-deficient mESC^WAPL/CTCF-AID^	This study	N/A
hESC (H9 cell line)	Wicell Institute	Cat #WA09
**Recombinant DNA**		
CTCF-mAC donor	Addgene	Cat #140646
pEN244	Addgene	Cat #92140
pEN113	Addgene	Cat #86233
pX330-EN479	Addgene	Cat #86234
LentiCRISPR v2	Addgene	Cat #52961
pSpCas9(BB)-2A-GFP (PX458)	Addgene	Cat #48138
PB-TRE-dCas9-VPR	Addgene	Cat #63800
mWAPL-2A-FKBP-GFP (recombination vector)	This study	N/A
hWAPL-2A-FKBP-GFP (recombination vector)	This study	N/A
hCTCF-2A-FKBP-GFP (recombination vector)	This study	N/A
PX458-mARID3A-1	This study	N/A
PX458-mARID3A-2	This study	N/A
PX458-mARID3A-3	This study	N/A
PX458-mDPPA2-1	This study	N/A
PX458-mDPPA2-2	This study	N/A
PX458-mDPPA2-3	This study	N/A
**Antibodies**		
ZSCAN4C	Millipore Sigma	Cat #AB4340
CTCF	Millipore Sigma	Cat #07-729
WAPL	Proteintech	Cat #16370-1-AP
TUBULIN	Millipore Sigma	Cat #T9026
DPPA2	Millipore Sigma	Cat #MAB4356
HRP Goat anti-Rabbit IgG	Thermo Fisher Scientific	Cat #31466
HRP Goat anti-Mouse IgG	Thermo Fisher Scientific	Cat #31431
HA	Cell Signaling	Cat #6E2
HA	Cell Signaling	Cat #3724
NUCLEOLIN	Cell Signaling	Cat #14574
NELFA	Bethyl Laboratories	Cat #A301-910A
Guinea Pig anti-Rabbit IgG	Antibodies-online	Cat #ABIN101961
Chicken anti-Mouse IgG (H + L), Alexa Fluor™ 647	Thermo Fisher Scientific	Cat #A21463
Chicken anti-Rabbit IgG (H + L), Alexa Fluor™ 647	Thermo Fisher Scientific	Cat #A21443
Chicken anti-Rabbit IgG (H + L), Alexa Fluor™ 488	Thermo Fisher Scientific	Cat #A21441
**Oligonucleotides and other sequence-based reagents**		
Oligonucleotide sequence information	This paper	Table EV3
**Chemicals, enzymes and other reagents**		
DMEM High Glucose	ThermoFisher Scientific	Cat #11965-092
Non-essential amino acids	ThermoFisher Scientific	Cat #11140-050
Glutamax	ThermoFisher Scientific	Cat #35050-061
β-mercaptoethanol	ThermoFisher Scientific	Cat #31350-010
Penicillin/streptomycin	ThermoFisher Scientific	Cat #15140-122
Gelatin	Sigma-Aldrich	Cat #G1890
Fetal Bovine Serum	Gibco	Cat #10439-024
Trypsin 0.05%	ThermoFisher Scientific	Cat #25300-054
Gibson assembly	New England Biolabs	Cat #M5510A
jetPRIME	PolyPlus	Cat #101000015
Indole-3-acetic acid	Sigma-Aldrich	Cat #I5148
dTAGv	Tocris Biosciences	Cat #6914
Matrigel (growth factor reduced)	Corning	Cat #356231
mTeSR plus	Stem Cell Technologies	Cat #100-0276
Dispase	Stem Cell Technologies	Cat #07923
Accutase	Gibco	Cat #A11106-01
DMEM/F12	ThermoFisher Scientific	Cat #11320-033
Neurobasal medium	ThermoFisher Scientific	Cat #21103-049
N2 supplement	Gibco	Cat #17502048
B27 supplement	Gibco	Cat #17504044
TryplE	Gibco	Cat #12604-013
EGF	PeproTech	Cat #315-09
FGF2/bFGF	R&D Systems	Cat #233-FB-025
Alexa Fluor 647-azide	Vector Laboratories	Cat #CCT-1299
16% paraformaldehyde	Electron Microscopy Sciences	Cat #15710
μSlide 8 well coverslips	Ibidi	Cat #80826
Cot-1 DNA	Invitrogen	Cat #18440-016
Concanavalin A beads	Polysciences	Cat #86057-3
Digitonin	Sigma-Aldrich	Cat #300410
BSA	Millipore Sigma	Cat #03117332001
Glycogen	Roche	Cat #64438320
RNase A	ThermoFisher Scientific	Cat #EN03521
Proteinase K	Meridian Biosciences	Cat #BIO-37084
AMPure XP beads	Beckman Coulter	Cat #A63880
Tn5 Transposase	Illumina	Cat #20034197
Zymo DNA Clean and Concentrator kit	Zymo	Cat # D4033
Q5 Polymerase	New England Biolabs	Cat #M0491S
T4 DNA polymerase	New England Biolabs	Cat #M0203L
T4 Polynucleotide Kinase	New England Biolabs	Cat #M0201L
Klenow fragment	New England Biolabs	Cat #M0210L
Klenow fragment exo-	New England Biolabs	Cat #M0212S
Quick Ligase	New England Biolabs	Cat #M2200L
35 mm gridded glass bottom dishes	MatTek Corporation	Cat #P35G-1.5-14-C-GRID
5-ethynyl uridine	Vector Laboratories	Cat #CCT-1261
MyTaq HS DNA Polymerase	Meridian Biosciences	Cat #BIO21111
SensiFAST cDNA Synthesis Kit	Meridian Biosciences	Cat #BIO65054
ISOLATE II RNA Mini Kit	Meridian Biosciences	Cat #BIO52072
PowerUp™ SYBR™ Green Master Mix for qPCR	ThermoFisher Scientific	Cat #A25742
SuperSignal West Pico PLUS	ThermoFisher Scientific	Cat #34580
SuperSignal West Femto	ThermoFisher Scientific	Cat #34094
KAPA Library Quantification Kit for Illumina Platforms	Roche – KAPA Biosystems	Cat #07960140001
2X Kapa HiFi HotStart Ready mix	Roche – KAPA Biosystems	Cat #08202940001
NEBNext Ultra II Directional RNA Library Prep Kit	New England Biolabs	Cat #E7760S
NEBNext® rRNA Depletion Kit v2	New England Biolabs	Cat #E7400L
**Software**		
Signal Image Artist	Revvity signals (https://revvitysignals.com/products/research/signals-image-artist)	N/A
R (version 4.3.1)	https://www.r-project.org/	N/A
Prism (version 10.4.0)	GraphPad	N/A
Imaris (version 10.2)	Oxford Instruments	N/A
fastp v.0.20.0.	https://github.com/OpenGene/fastp	N/A
bowtie2 v2.2.6	https://sourceforge.net/projects/bowtie-bio/files/bowtie2/2.2.6/	N/A
macs2 v2.1.0	Zhang et al, 2008	N/A
Cutadapt (version 4.4)	Martin, 2011	N/A
deepTools2	Ramírez et al, 2016	N/A
STAR v2.7.6a	Dobin et al, 2013	N/A
tetoolkit v2.1.4	Jin et al, 2015	N/A
DESeq2 package	Love et al, 2014	N/A
limma (version 3.62.1)	https://bioconductor.org/packages/release/bioc/html/limma.html	N/A
COMBAT from the sva (version 3.54.0)	https://bioconductor.org/packages/release/bioc/html/sva.html	N/A
ATAC-seq PipEliNe (version 0.5.2)	(https://github.com/CCBR/ASPEN	N/A
TOBIAS (version 0.13.3)	Bentsen et al, 2020	N/A
HOCOMOCO mouse core motifs (version 11)	Kulakovskiy, et al, 2018	N/A
3D Slicer	https://www.slicer.org	N/A
SerialEM	Mastronarde, 2005	N/A
IMOD software package (version 4.11)	Mastronarde and Held, 2017	N/A
RTA (version 3.4.4)	Illumina	N/A
cellranger-arc (version 2.0.2)	10x Genomics	N/A
Seurat (version 5.1.0)	Stuart et al, 2021	N/A
Signac (version 1.14.0)	Hao et al, 2024	N/A
SCTransform	Hafemeister and Satija, 2019	N/A
JASPAR 2020 database	Fornes et al, 2020	N/A
monocle3 (version 1.3.7)	Qiu et al, 2017, Trapnell et al, 2014	N/A
**Other**		

### Cell culture

Wild-type (R1, G4, E14) mESC, mESC^DUX^ (doxycycline-inducible mESC expressing DUX) (Olbrich et al, 2021), mESC^WAPL-FKBP^, mESC^CTCF-AID^ (ID: EN52.9.1, a generous gift from Dr. Elphege Nora, UCSF, San Francisco, USA) and cell derivatives, DUX-deficient mESC (a generous gift from Dr. Didier Trono, Laboratory of Virology and Genetics, EPFL, Lausanne, Switzerland) and cell derivatives, mESC^WAPL-AID^ and mESC^WAPL/CTCF-AID^ (a generous gift from Dr. de Wit, NKI, Amsterdam, The Netherlands) were grown on 0.1% gelatin-coated plates in high-glucose DMEM (Gibco) supplemented with 15% FBS, 1:500 LIF (made in house), 0.1 mM non-essential amino acids, 1% glutamax, 55 mM β-mercaptoethanol, and 1% penicillin/streptomycin (all from ThermoFisher Scientific) at 37 °C and 5% CO_2_. Alternatively, mESC cells were also grown on a feeder layer of growth-arrested MEFs. Cells were routinely passaged with Trypsin 0.05% (Gibco). Media was changed every other day and passaged every 2-3 days.

To generate mESC^WAPL-FKBP^ cell lines in a wild-type (R1 or G4) or mESC^DUX^ background and in *Dux*-deficient mESC^WAPL-FKBP^, we made a targeting construct using the backbone from the CTCF-mAC donor (gift from Masato Kanemaki, 140646, Addgene) and containing recombination arms from the *Wapl* locus flanking a 2A-FKBP-GFP sequence all amplified by PCR and followed by Gibson assembly (New England Biolabs) (Table EV3). To generate *Dux*-deficient mESC^CTCF-AID^ lines we used the recombination plasmid pEN244 and pEN113 (gifts from Elphege Nora, 92140 and 86233, Addgene). For all newly generated cell lines, recombination plasmids together with a vector expressing Cas9 and sgRNAs targeting the sequences surrounding the stop codon of *Ctcf* or *Wapl* loci (gifts from Elphege Nora and Feng Zhang, 86234 and 52961, respectively, Addgene; Table EV3) were co-transfected using jetPRIME (PolyPlus transfection). Successful targeting events were sorted on a BD FACSAria Fusion instrument based on GFP expression and all independent mESC clonal lines were tested for CTCF or WAPL depletion following IAA or dTAG treatments correspondingly. To generate ARID3A and DPPA2 knockout mESC^WAPL/CTCF-AID^, we cloned sgRNAs in the plasmid pX458 which also expresses Cas9 and GFP (gift from Feng Zhang, Addgene 48138) and transfected cells using jetPRIME. GFP-positive cells were sorted and plated on a feeder layer of growth-arrested MEFs to establish new clonal lines. We performed western blots (for DPPA2) or followed a PCR-based approach (for ARID3A) to verify the gene knockouts. To generate doxycycline-inducible ARID3A expressing mESC^WAPL/CTCF-AID^, we ordered a gene fragment containing the HA-ARID3A coding sequence and cloned it in a piggybac-based vector (gift from George Church, Addgene 63800) by using Gibson assembly. Cells were co-transfected with a supertransposase encoding vector and selected with antibiotics to establish new clonal lines.

Human ESC (hESC, H9 cell line, Wicell Institute) were cultured in wells coated with growth factor reduced Matrigel (354230, Corning) in mTeSR plus medium (Stemcell, #100-0276) with daily changes of media. Cells were passaged at 80% confluency using 1 mg/mL Dispase (Stemcell, #07923) and manual scrapping and cultured at 37 °C and 5% CO_2_. To generate hESC^CTCF-FKBP^ and hESC^WAPL-FKBP^ knock-in lines, we made a targeting construct using the backbone from the CTCF-mAC donor (Gift from Masato Kanemaki, 140646, Addgene) and containing recombination arms from the *Ctcf* or *Wapl* locus flanking an FKBP-2A-GFP sequence after amplifying the corresponding sequences by PCR followed by Gibson assembly (New England Biolabs) (Table EV3). hESC were individualized with Accutase and electroporated using a NEPA21 electroporator system (Nepagene) following manufacturer’s recommendations with the recombination vector and a vector expressing Cas9 and sgRNAs targeting the sequences surrounding the stop codon of *CTCF* or *WAPL* gene (LentiCRISPRv2, gift from Feng Zhang, 52961, Addgene). Positive targeting events were sorted on a BD FACSAria Fusion instrument. All experiments conducted with hESC followed protocols approved by the National Institutes of Health ESCRO committee.

To induce differentiation of mESC^WAPL-AID^ and mESC^WAPL/CTCF-AID^ toward neural progenitor stem cells (mNSCs) we seeded 0.5 × 10^6^ mESC^WAPL-AID^ and mESC^WAPL/CTCF-AID^ in a 10 cm plate. The following day, media was changed to N2/B27 medium: DMEM/F12 and Neurobasal (1:1), N2 supplement, B27 supplement, 1% glutamax, 55 mM β-mercaptoethanol, and 1% penicillin/streptomycin (all from ThermoFisher Scientific) and refreshed daily for a total of 7 days. On day 7, cells were dissociated with TryplE (Gibco) and 3 × 10^6^ cells were plated in low-binding plates in N2B27 with 10 ng/mL EGF (PeproTech) and FGF (R&D Systems) to promote the growth in suspension as spheres. Three days later, cell aggregates were plated in gelatinized plates and grown as a monolayer of mNPCs in N2B27 with 10 ng/mL EGF/FGF. After 2–4 days, cells were passaged at least five times with Accutase before performing experiments. We performed the same experiments on mNSC^WAPL-AID^ and mNSC^WAPL/CTCF-AID^ derived from two independent ESC clonal lines with similar results. However, only one set of mNSC lines is shown in this manuscript. None of the cell lines used in this manuscript are listed in the International Cell Line Authentication Committee (ICLAC) Database of Cross-contaminated or Misidentified Cell Lines. Testing for mycoplasma contamination in our cells was performed routinely.

### RNA extraction and qPCR

Isolation of total RNA was performed by using the Isolate II RNA Mini Kit (Bioline) followed by cDNA synthesis (SensiFAST cDNA Synthesis Kit, Bioline). Quantitative real time PCR was performed with PowerUp SYBR Master mix in a QuantStudio 6 Pro system. When analyzing quantitative real time PCR data, we considered statistically significant those samples that when compared showed an unpaired one-tail *t* test with a *P* value of at least 0.05 or lower. Three independent experiments with three replicates including at least two independent clonal cell lines were performed but only one is shown in the figures.

### Western blot

Cells grown in culture were trypsinized and lysed in 50 mM Tris pH 8, 8 M Urea (Sigma) and 1% Chaps (Millipore) followed by 30 min of shaking at 4 °C. A total of 25 μg of extracts were run on 4–12% NuPage Bis-Tris Gel (Invitrogen) and transferred onto Nitrocellulose Blotting Membrane (GE Healthcare). Transferred membranes were incubated with the following primary antibodies overnight at 4 °C: ZSCAN4C (1:500, AB4340, Millipore Sigma), CTCF (1:1000, 07-729, Millipore), WAPL (1:1000, 16370-1-AP, Proteintech), DPPA2 (MAB4356, 1:2000; Millipore Sigma) and TUBULIN (1:50000, T9026, Sigma-Aldrich). The next day the membranes were incubated with HRP-conjugated secondary antibodies Goat anti-Rabbit IgG (H + L) (1:5000; Thermo Fisher Scientific, Cat# 31466) or Goat anti-Mouse IgG (H + L) (1:5000; Thermo Fisher Scientific, Cat# 31431) for 1 h at room temperature. Membranes were developed using SuperSignal West Pico PLUS or SuperSignal West Femto Maximum sensitivity (Thermo Scientific). For westerners, two independent experiments were performed but only one representative is shown in the figures.

### High throughput imaging (HTI)

A total of 10,000 mESC were plated on gelatinized μCLEAR bottom 96-well plates (Greiner Bio-One, 655087). mESC were incubated with 500 μM Indole-3-acetic acid (IAA, Sigma) or 500 nM dTAGv (Tocris Bioscience) for the indicated times depending on the experiment before fixation with 4% PFA in PBS for 10 min at room temperature followed by immunostaining when needed. Briefly, cells were permeabilized for 10 min using the following permeabilization buffer (100 mM Tris-HCl pH 7.4, 50 mM EDTA pH 8.0, 0.5% Triton X-100). mESC were incubated overnight with ZSCAN4C (1:500, AB4340, Millipore Sigma) primary antibodies, washed in 0.1% Tween in 1× PBS, and incubated with the secondary antibody accordingly for one hour at room temperature. For 5-ethynyl uridine (EU) analyses cells were incubated with 10 μM EU (Click Chemistry Tools) for 30 min before fixation with 4% PFA in PBS for 10 min at room temperature. EU incorporation was visualized using Alexa Fluor 647-azide (Click Chemistry Tools) Click-iT labeling chemistry and DNA was stained using DAPI (4′,6-diamidino-2-phenylindole). Images were automatically acquired using a CellVoyager CV7000 or CV8000 high throughput spinning disk confocal microscope (Yokogawa, Japan). Each condition was always performed in triplicate wells and at least nine different fields of view (FOV) were acquired per well. Image analysis was performed using the Signals Image Artist system (Revvity Signals). Nuclei were segmented based on DAPI and mean fluorescence intensities from nuclei were quantified. Cell-level data was exported as text files, then analyzed and plotted using R version 4.3.1 or GraphPad Prism version 10.4.0. When analyzing HTI data, we considered statistically significant those samples that when compared showed a 1.5-fold difference in the averaged mean (and percentage of ZSCAN4-positive cells where correspond), and an unpaired one-tailed *t* test with a *P* value of at least 0.05 or lower. For all HTI experiments, three independent experiments including at least two independent clonal cell lines were performed but only one representative experiment is shown in the figures.

### Immunofluorescence

A total of 50,000 mESC were plated on gelatinized μSlide 8 well coverslips (Ibidi). mESC were incubated with 500 μM Indole-3-acetic acid (IAA, Sigma) or 150 ng/ml Doxycycline for the indicated times depending on the experiment before fixation with 2% PFA in PBS for 10 min at room temperature. Cells were permeabilized for 10 min using the following permeabilization buffer (100 mM Tris-HCl pH 7.4, 50 mM EDTA pH 8.0, 0.5% Triton X-100) and incubated overnight with DPPA2 (1:500, MAB4356, Millipore Sigma) or NELFA (1:500, Bethyl Laboratories, A301-910A) primary antibodies, washed in 0.1% Tween in 1× PBS, and incubated with the secondary antibody accordingly for one hour at room temperature.

### DNA FISH

mESC lines were grown in glass coverslips and fixed in 4% paraformaldehyde (Electron Microscopy Sciences) for 10 min at room temperature, washed three times with PBS 1× and permeabilized for additional 20 min using the following permeabilization buffer (100 mM Tris-HCl pH 7.4, 50 mM EDTA pH 8.0, 0.5% Triton X-100). Cells were incubated for 15 min in 0.1 N HCl at room temperature. HCl was removed and cells were treated with 2× SSC for 5 min to neutralize the acid. Coverslips were then transferred to equilibration buffer (1 volume of 4× SSC/1 volume of deionized formamide) for 30 min at room temperature. Equilibration buffer was removed and hybridization buffer (1 volume of 2× SSC, 0.1% Tween and 15% dextran sulfate/1 volume of deionized formamide) containing a mix of 20 nM *Dux* probes (a total of 25 probes to detect *Dux* DNA, Table EV3), 140 nM of a 647 Alexa-fluor conjugated readout probe (Table EV3) and 10μg of Cot-1 DNA was added to the coverslip, heated at 80 C for 7.5 min and immediately transferred to a hybridization chamber at 37 C overnight. Following hybridization, coverslips were washed three times for 5 min with 2× SSC at 42 °C, three times for 5 min with 2× SSC at 60 °C and three times for 5 min with PBS. After the washes, cells were incubated with blocking buffer 30 min at room temperature followed by incubation with primary antibodies against nucleolin (1:1000, Cell Signaling, D4C70, #14574) for 3 h and with a secondary antibody accordingly for one hour at room temperature. Confocal images were acquired using a Nikon CSU-W1 spinning disk microscope (Nikon) equipped with a ×60 objective (N.A. 1.49) and Photometrics Prime BSI sCMOS camera. Images were denoised and deconvolved in Nikon Elements software using Denoise.AI and Lucy-Richardson deconvolution, respectively. Nuclei and nucleoli were segmented based on DAPI and nucleolin intensity, respectively, using custom-trained Cellpose models. AI models were trained using the NIH HPC Biowulf cluster (https://hpc.nih.gov). Labeled segments were used to create Surfaces in Imaris (v. 10.2, Bitplane) and *Dux* FISH foci were identified using the Surfaces module. Fluorescence intensities and positional data for DUX FISH foci and nucleoli were quantified using Imaris, while keeping track of each parent nuclei. Data analysis was performed using R. The minimum distance of every FISH foci to the nucleoli within the same parent nucleus was calculated.

### CUT&RUN

The CUT&RUN protocol was performed as described with some variations (Skene et al, 2017; Meers et al, 2019). In brief, 450,000 hESC were individualized with Accutase, washed a total of three times with Wash Buffer (20 mM HEPES-KOH pH 7.5, 150 mM NaCl, 0.5 mM spermidine, Roche complete Protease Inhibitor tablet EDTA free) and bound to activated Concanavalin A beads (Polysciences) for 10 min at room temperature. Cell permeabilization was performed in 200 μl of Digitonin Buffer (0.05% Digitonin and 0.1% BSA in Wash Buffer) followed by incubation with 4μl of the antibody against CTCF (07-729, Millipore) at 4 °C for 2 h. For negative controls, we used a Guinea Pig anti-Rabbit IgG (ABIN101961, Antibodies-online). After antibody incubation, cells were washed with Digitonin Buffer, followed by incubation with in-house purified hybrid protein A-protein G-Micrococcal nuclease (pAG-MNase) at 4 °C for 1 h. Samples were washed again in Digitonin Buffer, resuspended in 150 μl Digitonin Buffer and equilibrated to 0 °C on ice water for 5 min. In total, 3 μl of 100 mM CaCl_2_ was added to cells to start with MNase cleavage, and after 1 h of digestion, reactions were stopped with the addition of 150 μl 2× Stop Buffer (340 mM NaCl, 20 mM EDTA, 4 mM EGTA, 0.02% Digitonin, 50 μg/ml RNase A, 50 μg/ml Glycogen). Samples were incubated at 37 °C for 10 min to release DNA fragments and centrifuged at 16,000× *g* for 5 min. Supernatants were collected and a mix of 1.5 μl 20% SDS/2.25 μl 20 mg/ml Proteinase K was added to each sample followed by incubation at 65 °C for 35 min. DNA was precipitated with ethanol and sodium acetate and pelleted by centrifugation at 4 °C, washed, air-dried and resuspended in 10 μl 0.1× TE.

### CUT&RUN library preparation and sequencing

We used half of the precipitated DNA obtained from CUT&RUN to prepare Illumina compatible sequencing libraries. In brief, end-repair was performed in 50 μl of T4 ligase reaction buffer, 0.4 mM dNTPs, 3 U of T4 DNA polymerase (NEB), 9 U of T4 Polynucleotide Kinase (NEB) and 1 U of Klenow fragment (NEB) at 20 °C for 30 min. Following this step, end-repair reaction was cleaned using AMPure XP beads (Beckman Coulter) and eluted in 16.5 μl of Elution Buffer (10 mM Tris-HCl pH 8.5) followed by A-tailing reaction in 20 μl of dA-Tailing reaction buffer (NEB) with 2.5 U of Klenow fragment exo- (NEB) at 37 °C for 30 min. The total volume of the A-tailing reaction was mixed with Quick Ligase buffer 2X (NEB), 3000 U of Quick Ligase (NEB) and 10 nM of annealed adaptor (Illumina truncated adaptor) in a final volume of 50 μl and incubated for 20 min at room temperature. The adaptor was prepared by annealing the following HPLC-purified oligos: 5′-Phos/GATCGGAAGAGCACACGTCT-3′ and 5′-ACACTCTTTCCCTACACGACGCTCTTCCGATC^∗^T-3′ (^∗^phosphorothioate bond). Ligation was stopped by adding 50 mM of EDTA, cleaned with AMPure XP beads and eluted in 14 μl of Elution Buffer. All volume was used for PCR amplification in a 50 μl reaction with 1 μM primers TruSeq barcoded primer p7, 5′-CAAGCAGAAGACGGCATACGAGATXXXXXXXXGTGACTGGAGTTCAGACGTGTGCTCTTCCGATC^∗^T-3′ and TruSeq barcoded primer p5 5′-AATGATACGGCGACCACCGAGATCTACACXXXXXXXXACACTCTTTCCCTACACGACGCTCTTCCGATC*T-3′ (^∗^ represents a phosphothiorate bond and XXXXXXXX a barcode index sequence), and 2× Kapa HiFi HotStart Ready mix (Kapa Biosciences). PCR amplification was performed with the following program: 45 s at 98 °C followed by 15 cycles of 15 s at 98 °C, 30 s at 63 °C, 30 s at 72 °C and a final 5 min extension at 72 °C. PCR reactions were cleaned with AMPure XP beads (Beckman Coulter), run on a 2% agarose gel and a band of 300 bp approximately was cut and gel purified using QIAquick Gel Extraction Kit (QIAGEN). Library concentration was determined with KAPA Library Quantification Kit for Illumina Platforms (Kapa Biosystems). Sequencing was performed on the Illumina NextSeq550 (75 bp pair-end reads).

### CUT&RUN data processing and analysis

Data were processed as previously reported (Ramírez et al, 2016; Martin, 2011) (10.5281/zenodo.10658411). Here, reads were adapter trimmed using fastp v.0.20.0. An additional trimming step was performed to remove up to 6 bp adapter from each read. Next, reads were aligned to the hg38 genome using bowtie2 v2.2.6 with the ‘dovetail’ and ‘sensitive’ settings enabled. Peaks were called using macs2 v2.1.0 (Zhang et al, 2008) with a q-value cutoff of <0.01. Signal tracks normalized to 1× coverage (reads per genomic coverage, RPGC) were generated using the ‘bamCoverage’ utility from deepTools v3.3.0 (Ramírez et al, 2016) with parameters bin-size=25, smooth length=75, and ‘center_reads’ and ‘extend_reads’ options enabled.

### ChIP-seq

Twenty million of untreated or IAA-treated mESC^WAPL/CTCF-AID^ cells were fixed using 1% Formaldehyde (Sigma, F1635) at room temperature for 10 min. Fixation was then quenched with 125 mM glycine (Sigma). Cell pellets were washed twice with cold PBS and samples were snap-frozen and stored in −80 °C. Frozen pellets were resuspended in 1 mL RIPA buffer (10 mM Tris-HCl pH 7.5, 1 mM ethylenediaminetetraacetic acid (EDTA), 0.1% sodium dodecyl sulfate, 0.1% sodium deoxycholate, 1% Triton X-100, and 1 Complete Mini EDTA-free proteinase inhibitor tablet (Roche)). Sonication was performed using Covaris S220 (duty cycle 20%, peak incident power 175, and cycle/burst 200 for 30 min at 4 °C). Chromatin was pre-clarified with 40 μL prewashed Dynabeads Protein G (ThermoFisher) for 30 min at 4 °C and then incubated with 40 μL Dynabeads Protein G bound to 5 μg of anti-DPPA2 antibody (Millipore Sigma MAB4356) in 900 μL of RIPA buffer overnight at 4 °C. Beads were then collected in a magnetic separator (DynaMag-2 Invitrogen), washed twice with cold RIPA buffer, twice with RIPA buffer containing 0.3 M NaCl, twice with LiCl buffer (0.25 M LiCl, 0.5% Igepal-630, 0.5% sodium deoxycholate), once with TE (10 mM Tris pH 8.0, 1 mM EDTA) + 0.2% Triton X-100, and once with TE. Crosslinking was reversed by incubating the chromatin bound beads at 65 °C for 4 h in the presence of 0.3% SDS and 1 mg/mL of Proteinase K (Qiagen). Chromatin DNA extraction from beads and library preparation were performed as described (Canela et al, 2019).

### ChIPseq data processing and analysis

ChIPseq data were processed with version 2.1.0 of the nf-core/chipseq pipeline (citation: 10.5281/zenodo.3240506) using a custom mm10 genome containing an integrated DUX contig (Grow et al, 2021) with the--keep_multi_map parameter set to retain reads mapping to repetitive elements.

### RNA-seq libraries from ESC samples

RNA-seq libraries were prepared using NEBNext Ultra II Directional RNA Library Prep Kit for Illumina (New England Biolabs, NEB) and NEBNext rRNA Depletion Kit (Human/Mouse/Rat) (NEB) according to the manufacturer’s protocol. RNA quality and yield were measured using RNA integrity number (RIN) algorithm, using the Fragment Analyzer (Agilent) or LabChip Gx Touch 24 (Perkin Elmer). Samples with 7.0–10.0 RIN value were used for library preparation. In total, 2 μg of total RNA was used as input for VAHTS Stranded mRNA-seq Library preparation. Sequencing was performed on an Illumina NextSeq550 (75 bp pair-end reads).

### RNA-seq data processing and batch correction

Fastq files for publicly available RNA-seq experiments were downloaded from SRA. RNA-seq data were processed as previously reported (Vega-Sendino et al, 2024; Tillo, 2024). Briefly, RNA-seq reads were adapter trimmed using cutadapt v4.4 (Martin, 2011). Trimmed reads were aligned to the hg38 genome with the GENCODE transcriptome annotation release 35 using STAR v2.7.6a (Dobin et al, 2013). Transcript and transposable element expression was quantified using TEcount from tetoolkit v2.1.4 (Jin et al, 2015). Differential expression analysis was performed using the DESeq2 package (Love et al, 2014). To compare the RNA-seq experiments with public data, batch correction was performed. Gene counts across samples were first quantile-normalized using the limma v3.62.1 package. Batch correction was then performed on quantile-normalized counts using COMBAT from the sva v3.54.0 package.

### ATAC-seq analysis

ATAC-seq was performed as according previously reported with slight modifications (Buenrostro et al, 2013; Corces et al, 2017). Briefly, 50,000 cells were first washed with 50 μl cold 1× PBS and nuclei were isolated by resuspending the cells in 50 μl Lysis Buffer (10 mM Tris-HCl pH 7.5, 10 mM NaCl, 3 mM MgCl_2_, 0.1% IGEPAL CA-630, 0.1% Tween-20, and 0.01% Digitonin) on ice for three minutes. Nuclei were washed once with 1 ml Wash Buffer (10 mM Tris-HCl pH 7.5, 10 mM NaCl, 3 mM MgCl_2_, and 0.1% Tween-20) and further resuspended in 50 μl Transposition Reaction Mix (Tn5 Transposase, 1× TD Buffer, 0.1% Tween-20, 0.01% Digitonin) for exactly 30 min at 37 °C. Following transposition, DNA was purified using the Zymo DNA Clean and Concentrator kit (Zymo, D4033). Libraries were prepared using the entire purified DNA, Q5 Polymerase, and unique barcoded primers. An initial PCR amplification was performed at 72 °C for 5 min followed by five cycles of 98 °C, 10 s, 63 °C, 30 s, and 72 °C 1 min. Then, tubes were held on ice while a quantitative PCR was run on 5 μl of the pre-amplified library to determine the additional number of cycles needed. PCR reactions were cleaned up with AMPure XP beads (Beckman Coulter) and concentration was determined with KAPA Library Quantification Kit for Illumina Platforms (Kapa Biosystems). Sequencing was performed on the Illumina NextSeq550 (75 bp pair-end reads). ATAC-seq data were processed using the Center for Cancer Research Collaborative Bioinformatics Resource’s ASPEN (ATAC-seq PipEliNe) version 0.5.2 (https://github.com/CCBR/ASPEN) (Sevilla et al, 2024) to generate signal tracks (bigwigs) and peak calls using macs2 v2.1.0 (Zhang et al, 2008). Prediction of transcription factor occupancy from ATAC-seq signal was performed using TOBIAS v0.13.3 (Bentsen et al, 2020) using the HOCOMOCO mouse core motifs v11 (Kulakovskiy et al, 2018).

### Sample preparation for electron microscopy

In total, 50,000 mESC were grown on 35 mm gridded glass bottom dishes (MatTek Corporation) and treated with either IAA (test) or DMSO (control) for 48 h. Cells were imaged by confocal microscopy using a Zeiss LSM780 (Carl Zeiss Microscopy, LLC, Jena, Germany) equipped with a 63× plan-apochromat (N.A. 1.4) objective lens. Z-stack and/or extended field of view confocal fluorescence images were acquired with 0.09 μm x-y pixel size, 1.0 um z-step size, and pinhole set to 2.0 μm optical section thickness. Concomitant transmitted light images were collected using the Trans-PMT detector on the confocal microscope. Immediately following imaging, cells were fixed with Karnovsky’s fixative before transport. Plates with cells were post-fixed in 2% osmium tetroxide and 1.5% potassium ferricyanide in 0.1 M sodium cacodylate for 1 h at RT, washed with ultrapure water, and then stained with 1% aqueous uranyl acetate for 1 h at RT. After washing with water, the cells were treated with lead aspartate at 60 °C for 30 min and washed again. The samples were dehydrated through graded ethanol, with 10-min incubations each of 35%, 50%, 70%, 95%, and 100% with the 100% repeated 5 times. The plates were infiltrated with increasing amounts of Polybed 812 resin (hard formulation) with ethanol (resin: ethanol; 1:3, 1:2, 3:1, and 100%) and finally embedded in 100% degassed and incubated at 65 °C for 48 h. (EMS, Warrington, PA, USA). Glass coverslips were removed from the resin by heat shock. ROIs identified by LM were cut out using a jeweler’s saw and were either mounted on pin stubs, silver painted, and carbon-coated (10 nm) for FIB-SEM or mounted onto blank resin blocks with super glue for TEM tomography.

### FIB-SEM imaging and segmentation

Cell Regions of Interest (ROIs) previously identified by LM imaging were located using the gridded pattern in the resin left by the coverslip along with SEM imaging on a Zeiss Crossbeam 550 microscope at 1.5 kV (accelerating voltage) and beam current at 1.1 nA. After target cell identification, the FIB sample preparation workflow was initiated using the Fibics (Ottawa, CA) ATLAS3D sample preparation workflow. First, a 1-um platinum pad was laid over the target cell. Then, tracking and autofocus lines were milled into the platinum surface and covered by carbon deposition. Once the pad was complete, a coarse trench was milled using a 30 nA FIB beam, stopping just short of the front edge of the platinum pad. The trench face was then fine polished using a 3 nA beam. SEM imaging parameters were set at 1.5 kV accelerating voltage with a 1.1 nA beam current, a pixel resolution of 5 nm with a 15 nm slice thickness, and the in-lens EsB detector grid voltage was set at 820 V. The raw image stacks were then registered, inverted, and binned to generate volumetric reconstructions at 15 nm^3^ voxel using established protocols (Baena et al, 2021). Sub-volumes containing nucleoli were cropped from the original volume and segmented using 3D Slicer (https://www.slicer.org). Thresholding was utilized to define the fibular and granular material of each nucleolus. The resulting segments were smoothed, and extraneous features were removed using the scissors and islands functions of Slicer.

### Transmission electron tomography and analysis

Serial sections of ROIs identified by LM were cut *en face* on an ultramicrotome at a thickness of 250 nm and collected on formvar-coated copper slot grids that had been glow discharged. A 120 kV Talos L120C TEM (Thermo Fisher Scientific) was used to collect tilt series of nucleoli using a model 2020 advanced tomography holder (Fischione). Target areas were exposed to the electron beam for 10 min before tilt series collection to induce maximal resin section collapse. Images were recorded using a Ceta 16 M CCD camera (Thermo Fisher Scientific). Tilt series were recorded using SerialEM (Mastronarde 2005) software at a magnification corresponding to a pixel size of 0.966 nm with 1-degree tilt increments over an angular range of ±60°. Tomogram reconstructions were done in Etomo in the IMOD software package, version 4.11 (Mastronarde and Held, 2017), using patch tracking and weighted back-projection with the simultaneous iterative reconstruction-like filter. Tomogram movies were generated in Quicktime. Measurements of the fibrillar centers (i.e. cups) were done in 3Dmod by creating model contours for the major and minor diameters. At least two different tomographic slices were used for each tomogram measured with eight major and minor measurements of fibrillar centers made for each sample type (untreated control, treated control, and treated fluorescently positive).

### Multiome analysis

The nuclei isolation was performed according to 10x Genomics guidelines. In brief, 500,000 trypsinized mESC were washed with PBS + 0.04% BSA and then lysed in 100 μL Lysis Buffer (10 mM Tris-HCl pH 7.4, 10 mM NaCl, 3 mM MgCl_2_, 0.01% Tween-20, 0.01% IGEPAL CA-630, 0.001% Digitonin, 1% BSA, 1 mM DTT, 1 U/μl RNase inhibitor) for 5 min on ice. Immediately after, lysed cells were washed three times with 1 ml Wash Buffer (10 mM Tris-HCl pH 7.4, 10 mM NaCl, 3 mM MgCl_2_, 1% BSA, 0.1% Tween-20, 1 mM DTT, 1 U/μl RNase inhibitor) and resuspended in 100 μL Diluted Nuclei Buffer (1X Nuclei Buffer, 1 mM DTT, 1 U/μl RNase inhibitor). Nuclei concentration was determined using LUNA Automated Cell Counter. Single nucleus multiome libraries were prepared according to the 10x Genomics Multiome User Guide (CG000338). In short, nuclei were transposed and then partitioned with barcoded gel beads on a Chromium X controller. Transposed genomic DNA fragments and reverse-transcribed mRNA were single-cell barcoded, and libraries for ATAC and RNA-Seq were separated and processed in parallel. Sequencing libraries were prepared and quantified on an Agilent BioAnalyzer before being sequenced on an Illumina NovaSeq X Plus.

### Processing and analysis of snRNA-seq/ATAC-seq multiome data

Base calling was performed using RTA 3.4.4, demultiplexing was performed using cellranger-arc v2.0.2 (Bcl2fastq 2.20.0), and alignment was performed using cellranger-arc v2.0.2 (BWA 0.7.17-r1188). Sequenced reads were aligned to a 10x Genomics provided mouse reference sequence (refdata-cellranger-arc-mm10-2020-A-2.0.0). ATAC fragment files, per barcode metric files, and gene expression molecule info files for four samples were aggregated using cellranger-arc v2.0.2.

The filtered multiome matrices were processed using R packages Seurat (Stuart et al, 2021) (v5.1.0) or Monocle (Trapnell et al, 2014) for the RNA count data and Signac (Hao et al, 2024) (v1.14.0) for the chromatin assay. The gene expression count data was normalized using SCTransform (Hafemeister and Satija 2019), and dimensional reduction performed using the top variable features for principal component analysis (PCA). Uniform Manifold Approximation and Projection (UMAP) visualization was constructed using the first 50 PCs.

Peak calling was performed using the CallPeaks function of Signac in conjunction with Model-based Analysis of ChIP-seq macs2 v2.1.0 (Zhang et al, 2008). Latent semantic indexing (LSI) was performed on the ATAC fragment data using term frequency inverse document frequency (TF-IDF) for normalization and singular value decomposition (SVD) for dimensional reduction. UMAP visualization was constructed using LSI components 2 through 40. Peaks were annotated using the UCSC mouse mm10 reference genome and labelled using the ClosestFeature function of Signac (Fig. 2J). We used this function to identify the nearest genomic feature (usually a gene, exon or TSS) for a given set of ATACseq peaks. It links physical accessibility to the closest gene, and likely target, by providing the distance between the center of the ATACseq peak and the nearest genomic feature. The primary genomic features associated with a gene are open chromatin peaks (specifically, peak-based gene activity scores), which include the gene body and its promoter regions. Dataset EV4 highlights the gene features (‘closest region’) and their nature (type) associated to filtered ATACseq peaks from cells from clusters 2 and 4 (‘region’) as well as the distance to the feature (‘distance’).

Motif enrichment was performed using the AddMotifs function from Signac, with the UCSC mouse mm10 reference used for the genome and the JASPAR 2020 database (Fornes et al, 2020) used as the position frequency matrix (PFM). We then used the FindMotifs function in Signac to look for motif enrichment between differentially accessible peaks and used the RunChromVAR function of Signac to return a Seurat object with a motif activity assay.

Trajectory analysis was performed using the R package monocle3 (Qiu et al, 2017) (v1.3.7) by first converting the filtered multiome matrices into a cell dataset (CDS) which was then normalized and the pre-processed using the preprocess_cds and align_cds functions of monocle3. The learn_graph function from monocle3 was used to construct a trajectory graph, and root nodes from the Day 0 sample were used to order cells in pseudotime using the order_cells function.

To look for MERVL sequence activity, sequenced reads were aligned to a custom mouse reference that contained all annotated MERVL-int sequences. ATAC fragment files, per barcode metric files, and gene expression molecule info files for four samples were aggregated using cellranger-arc v2.0.2. RNA count data was processed as before, but ATAC data was annotated using an mm10.MERVL.bed file.

### Statistical analysis

For all statistical analyses in this study, Student’s *t* test was used for data with two groups. Number of independent cellular and molecular biology experiments and replicates are carefully described in Figure legends. Next-generation sequencing experiments (RNAseq and Cut&Run) and high-throughput microscopy analysis were performed in at least two independent humans or mouse ESC lines which is sufficient to determine reproducible results based on extensive experience and is widely accepted. No blinding was used in the experiments from this work. All results were reliably reproduced in multiple independent experiments as indicated in the figure legends. Randomization was not necessary for our study as it does not involve a case-control cohort experiment. No data were excluded from the analyses.

## Supplementary information

Appendix

Table EV1

Table EV2

Table EV3

Peer Review File

Dataset EV1

Dataset EV2

Dataset EV3

Dataset EV4

Dataset EV5

Dataset EV6

Dataset EV7

Dataset EV8

Dataset EV9

Dataset EV10

Movie EV1

Movie EV2

Source data Fig. 1

Source data Fig. 3

Source data Fig. 4

Source data Fig. 5

Source data Fig. 6

Figure EV1 Source Data

Figure EV2 Source Data

Figure EV3 Source Data

Figure EV4 Source Data

Figure EV5 Source Data

Expanded View Figures

## Data Availability

The datasets generated in this study are available in the following databases: ATAC-seq data: Gene Expression Omnibus GSE289302. RNA-seq data: Gene Expression Omnibus GSE289305. Cut&Run data: Gene Expression Omnibus GSE289306. Single Cell multiome data: Gene Expression Omnibus GSE290155. FIB-SEM data: EMPIAR-13260. (https://www.ebi.ac.uk/empiar/EMPIAR-13260/). The datasets obtained from publicly available sources include: RNA-seq data: Gene Expression Omnibus GSE85627. Gene Expression Omnibus GSE135180. ChIP-seq data: Gene Expression Omnibus GSE52457. Single-cell RNA-seq data: Gene Expression Omnibus GSE45719. Additional data and/or reagents that support the findings of this study are available from the corresponding author upon reasonable request. The source data of this paper are collected in the following database record: biostudies:S-SCDT-10_1038-S44318-026-00853-6.
