# Supplementary material for: Alterations in chromatin organization promote totipotent-like features in a DPPA2/DUX-dependent manner
Source: EMBO J. 2026 Jul 9;45(16):5657–88. doi: 10.1038/s44318-026-00853-6 (PMC13484482; doi:10.1038/s44318-026-00853-6)
Supplement: Supplementary file 1 [file 44318_2026_853_MOESM1_ESM.pdf]

Appendix for "Alterations in chromatin organization promote totipotent-like features in a DPPA2/DUX-dependent manner"

Table of contents:

Appendix Figure S1: Differentiation imposes a barrier for 2CLC conversion upon CTCF/WAPL depletion. page 2

Appendix Figure S2: WAPL/CTCF depletion leads to increased chromatin accessibility at DUX-bound sites. page 4

Appendix Figure S3: WAPL/CTCF depletion leads to transcriptional and chromatin accessibility changes over time. page 6

Appendix Figure S4: Gene modules analyses following WAPL/CTCF depletion. page 8

Appendix Figure S5: CTCF depletion in hESC leads to cell death. page 10

Appendix Figure S6: Nucleolar integrity is compromised in CTCF-depleted mESC. page 12

Appendix Figure S7: DUX-dependent 2CLC conversion in WAPL-depleted mESC. page 14

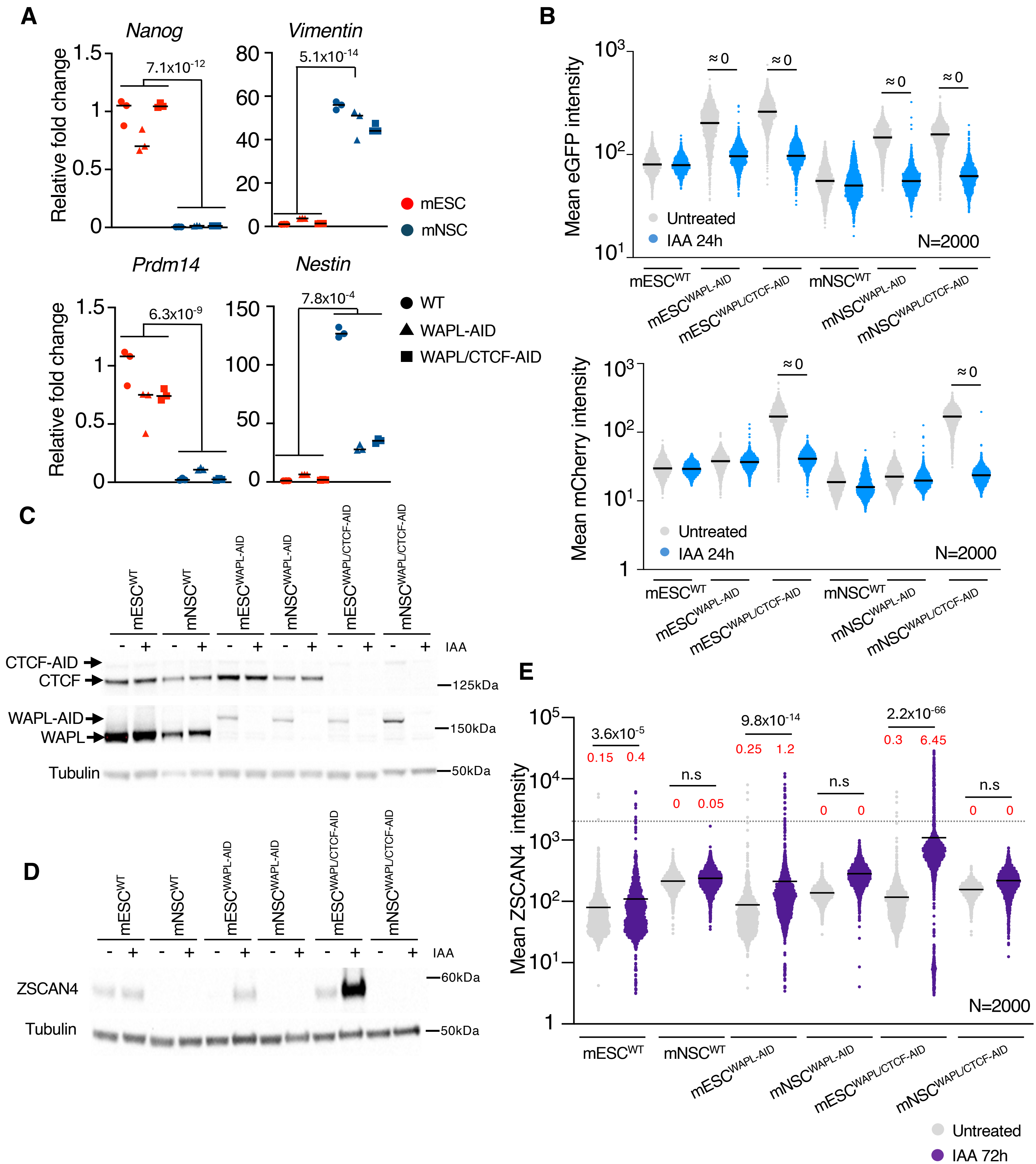

Appendix Figure S1

Appendix Figure S1. Differentiation imposes a barrier for 2CLC conversion upon CTCF/WAPL depletion.

(**A**) Plots showing the relative fold change expression of pluripotent genes (*Nanog* and *Prdm14*) and neuronal associated markers (*Nestin* and *Vimentin*) in mESC and mNSC with the indicated genotypes. p values are shown from one-tailed unpaired t-tests. Center line indicates the mean. Reactions were performed by triplicate in two independent experiments, but one representative is shown.

(**B**) High-throughput imaging quantification of eGFP (upper panel) and mCherry (lower panel) mean nuclear intensity in $mESC^{WT}$, $mESC^{WAPL\text{-}AID}$ and $mESC^{WAPL/CTCF\text{-}AID}$ as well as in $mNSC^{WT}$, $mNSC^{WAPL\text{-}AID}$ and $mNSC^{WAPL/CTCF\text{-}AID}$ that were untreated or treated with IAA for 24 hours. Center lines indicate mean values. N=2000; p values are shown from one-tailed unpaired *t*-tests. Two independent experiments were performed but only one representative is shown.

(**C,D**) Western blot analyses of WAPL, CTCF (**C**) and ZSCAN4 (**D**) performed in lysates from the same cell samples as in (**B**) that were untreated or treated with IAA for 48 hours. $mESC^{WT}$ and $mNSC^{WT}$ were included to show the increased size of the tagged proteins. Tubulin levels are shown as a loading control.

(**E**) High-throughput imaging quantification of ZSCAN4 mean nuclear intensity in cells that were untreated or treated with IAA for 72 hours using the same cell samples as in (**B**). Center lines indicate mean values. N=2000; p values are shown from one-tailed unpaired *t*-tests. Numbers in red show the percentage of $ZSCAN4^{+}$ cells above the arbitrary threshold. Two independent experiments were performed but only one representative is shown. n.s.= non-significant.

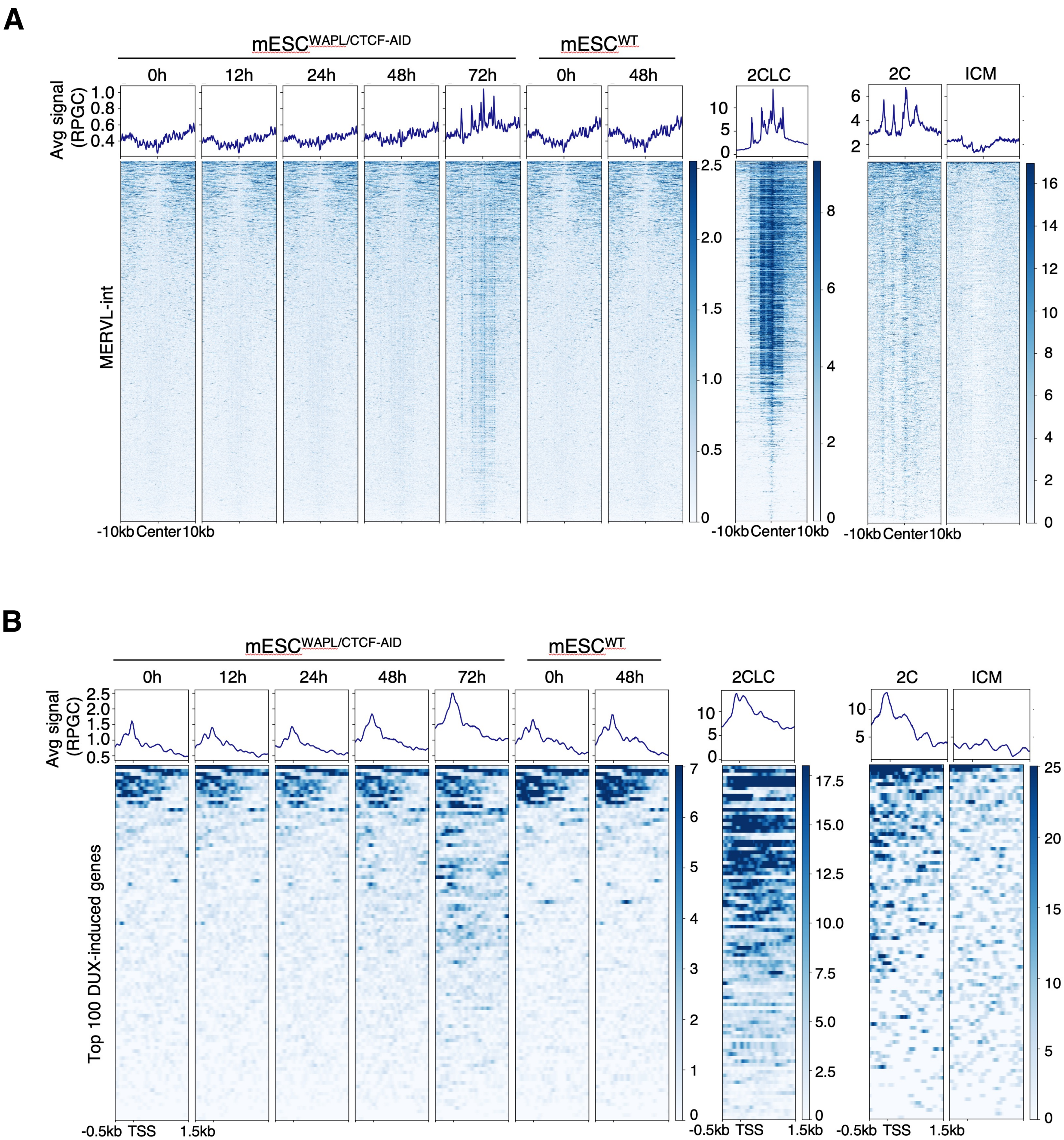

Appendix Figure S2

Appendix Figure S2. WAPL/CTCF depletion leads to increased chromatin accessibility at DUX-bound sites.

(**A,B**) Read density plots (RPGC) and corresponding heatmaps showing ATAC-seq read enrichment at the set of MERVL-int elements (**A**) or at the top 100 upregulated genes upon DUX expression (**B**). Samples include $mESC^{WT}$ and $mESC^{WAPL/CTCF\text{-}FKBP}$ that were untreated or treated with IAA for the indicated times. 2CLC are DUX-induced cells sorted based on the levels of RFP derived from the LTR-RFP reporter. ATACseq data from 2C (2-cell stage embryos) and ICM (inner cell mass) samples was obtained from Wu et al, 2016.

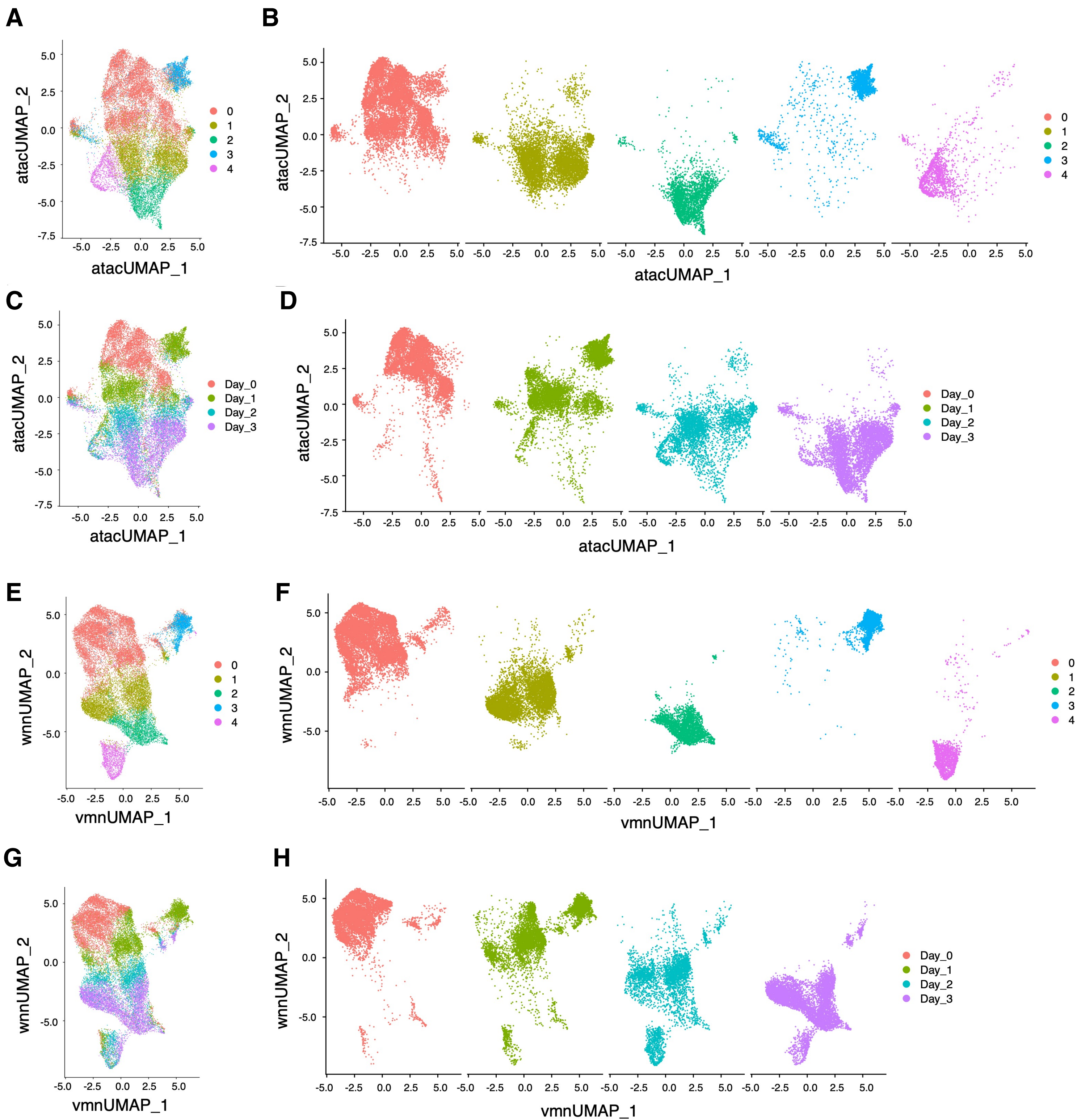

Appendix Figure S3

Appendix Figure S3. WAPL/CTCF depletion leads to transcriptional and chromatin accessibility changes over time.

(**A-D**) UMAPs of combined single-cell ATAC-seq data from untreated or IAA-treated mESC$^{WAPL/CTCF-FKBP}$ for 24, 48 and 72 hours showing representative clusters identified (**A,B**) or days of treatment (**C,D**).

(**E-H**) UMAPs of combined single-cell RNA-seq/ATAC-seq data using a weighted nearest neighbor (wnn) analysis from mESC$^{CTCF/WAPL-FKBP}$ that were untreated or treated with IAA for 24, 48 and 72 hours showing representative clusters identified (**E,F**) or days of treatment (**G,H**).

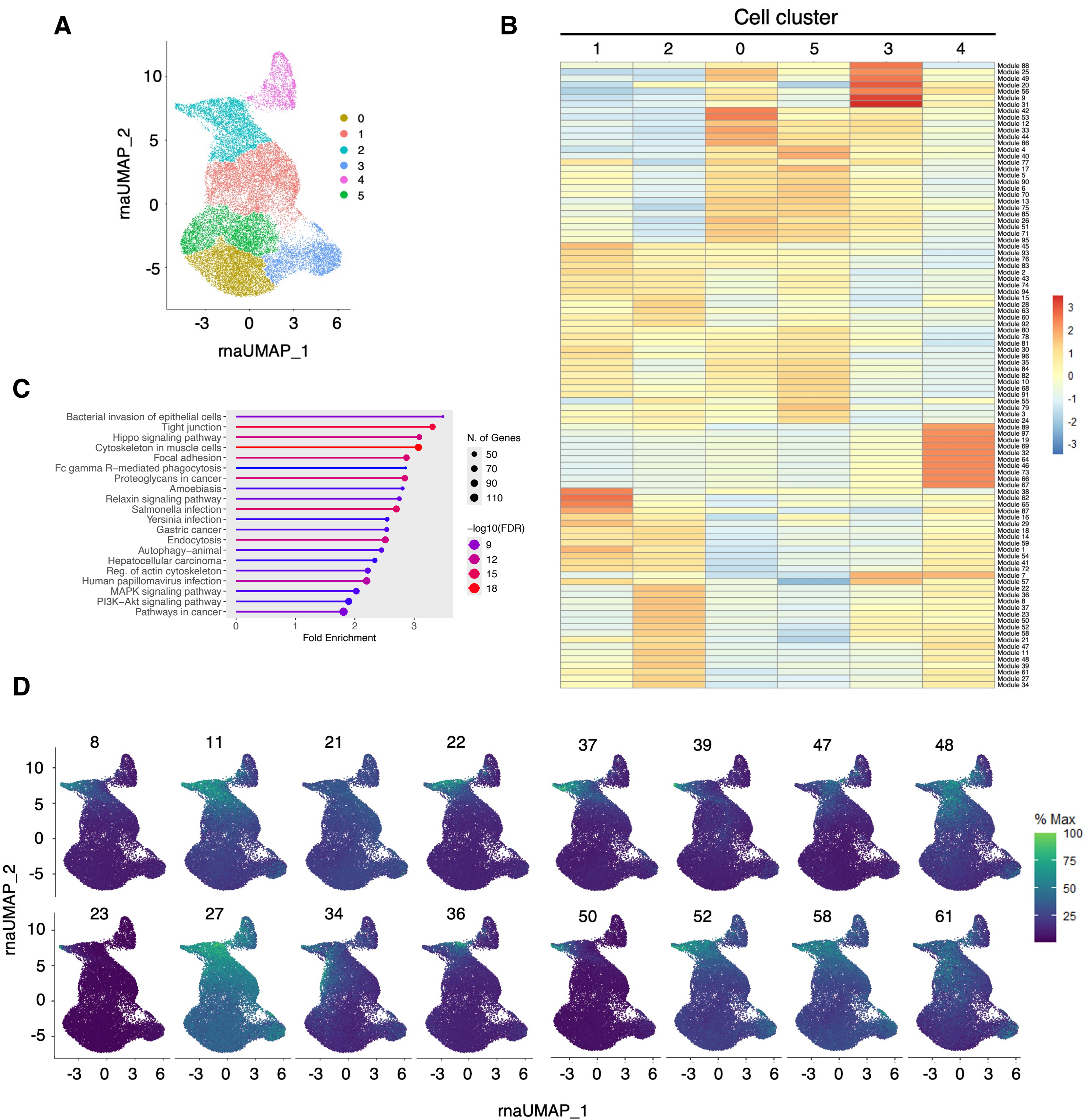

Appendix Figure S4

Appendix Figure S4. Gene module analysis following WAPL/CTCF depletion.

(A) UMAP of combined single-cell transcriptomes of $mESC^{CTCF/WAPL\text{-}FKBP}$ that were untreated or treated with IAA for 24, 48 or 72 hours with representative clusters identified using Monocle.

(B) Heatmap showing the gene modules differentially expressed in the clusters identified by Monocle.

(C) Gene ontology analysis on the gene modules differentially enriched in cell cluster 2.

(D) UMAPs visualization highlighting expression of the gene modules enriched in cell cluster 2.

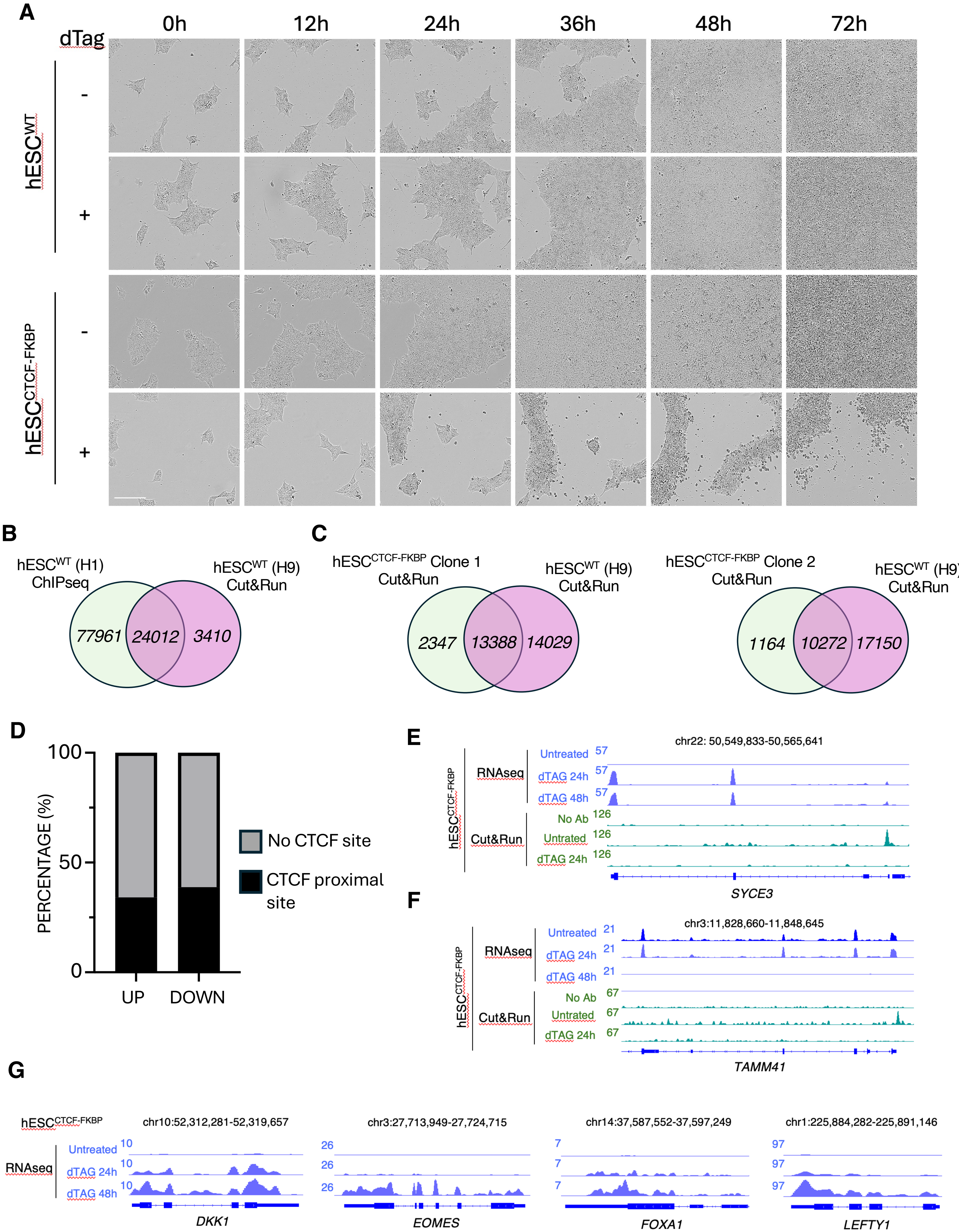

Appendix Figure S5

Appendix Figure S5. CTCF depletion in hESC leads to cell death.

(**A**) Bright field images obtained from a time-lapse experiment following $hESC^{WT}$ and $hESC^{CTCF\text{-}FKBP}$ that were untreated or treated with dTAG over time.

(**B,C**) Venn diagrams showing the overlap between CTCF bound sites from our Cut&Run experiment in $hESC^{WT}$ and CTCF bound sites obtained from a ChIPseq published dataset (Dixon et al, 2015) (**B**) or two representative $hESC^{CTCF\text{-}FKBP}$ clones (**C**).

(**D**) Plot showing the percentage of genes up or downregulated upon CTCF depletion containing a CTCF bound site within 50Kb of range.

(**E-G**) Genome browser tracks containing RNAseq and Cut&Run datasets from $hESC^{CTCF\text{-}FKBP}$ that were untreated or treated with dTAG containing examples of genes showing increased (**E**) or decreased expression (**F**) upon CTCF depletion. In (**G**) there are few examples of upregulated genes involved in differentiation.

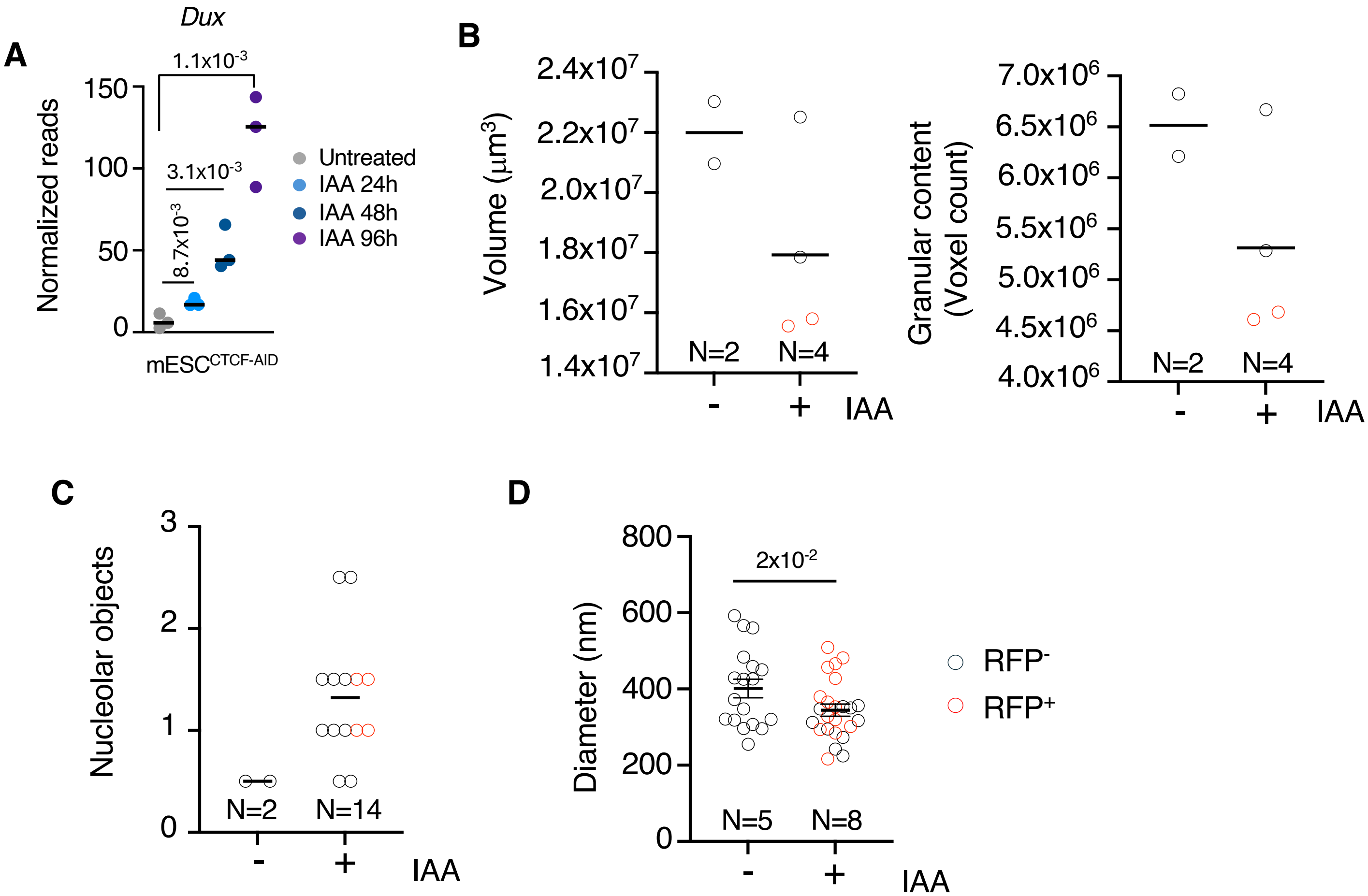

Appendix Figure s6

Appendix Figure S6. Nucleolar integrity is compromised in CTCF-depleted mESC.

(**A**) Plots showing normalized read counts obtained from RNAseq datasets corresponding to untreated or IAA-treated for the indicated times mESC$^{CTCF\text{-}AID}$. Data was obtained from Nora et al, 2017. p values are shown from one-tailed unpaired *t*-tests.

(**B,C**) Plots showing the nucleolar volume (**B,** left panel) and granular component (**B,** right panel), and nucleolar entities (**C**) obtained from FIB-SEM-derived 3D reconstructions from representative cells.

(**D**) Plot showing the maximum diameter from fibrillar centers measured in the indicated number of cells derived from transmission electron tomography images. p value is shown from one-tailed unpaired *t*-tests.

Data information: In **B-D**, measures from RFP+ CTCF-depleted cells identified by the MERVL-RFP reporter are shown in red.

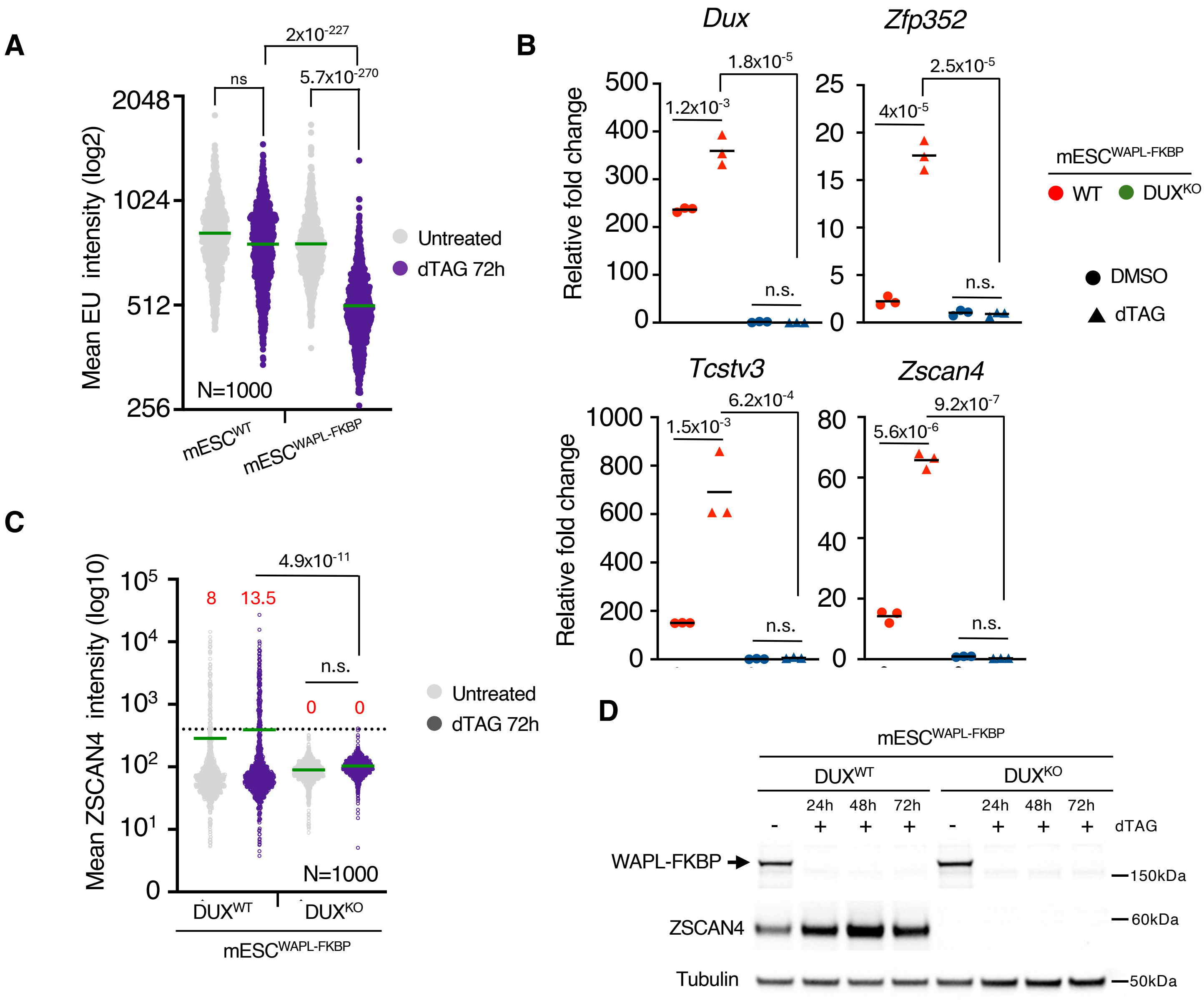

Appendix Figure S7

Appendix Figure S7. DUX-dependent 2CLC conversion in WAPL-depleted mESC.

(**A**) High-throughput imaging quantification of EU mean nuclear intensity in $mESC^{WT}$ and $mESC^{WAPL-FKBP}$ that were untreated or with dTAG for 72 hours. Center lines indicate mean values. p values are shown from one-tailed unpaired *t*-tests. n.s.= non-significant. Two independent experiments were performed but only one representative is shown.

(**B**) Plots showing the relative fold change expression of the indicated genes in wild-type and DUX-deficient $mESC^{WAPL-FKBP}$. p values are shown from two-tailed unpaired t-tests. Center line indicates the mean. Reactions were performed by triplicate in two independent experiments but one is shown.

(**C**) High-throughput imaging quantification of ZSCAN4 mean nuclear intensity in wild-type and DUX-deficient $mESC^{WAPL-FKBP}$ that were untreated or treated with dTAG for 72 hours. Center lines indicate mean values. p-values are shown from one-tailed unpaired *t*-tests. Numbers in red show the percentage of $ZSCAN4^{+}$ cells above the arbitrary threshold. Two independent experiments were performed but only one representative is shown.

(**D)** Western blot analyses of WAPL and ZSCAN4 performed in lysates from wild-type and DUX-deficient $mESC^{WAPL-FKBP}$ that were untreated or treated with dTAG for 24, 48 and 72 hours. Tubulin levels are shown as a loading control.
