# Supplementary material for: Alterations in chromatin organization promote totipotent-like features in a DPPA2/DUX-dependent manner
Source: EMBO J. 2026 Jul 9;45(16):5657–88. doi: 10.1038/s44318-026-00853-6 (PMC13484482; doi:10.1038/s44318-026-00853-6)
Supplement: Supplementary file 2 [file 44318_2026_853_MOESM28_ESM.pdf]

Expanded View Figures

Figure EV1. Supporting Fig. 1.

2CLC conversion is induced by the loss of WAPL or WAPL/CTCF. (**A**) Schematic representation of the targeting strategy used to generate the cell lines ($mESC^{WAPL\text{-}AID}$ and $mESC^{WAPL/CTCF\text{-}AID}$) used in this study. (**B**, **C**) High-throughput imaging quantification of eGFP (**C**) and mCherry (**D**) mean nuclear intensity in $mESC^{WT}$, $mESC^{WAPL\text{-}AID}$ and $mESC^{WAPL/CTCF\text{-}AID}$ that were untreated or treated with IAA 24 or 48 h. Center lines indicate mean values. *P* values are shown from one-tailed unpaired *t* tests. Two independent experiments were performed but only one representative is shown. (**D**) Western blot analysis of WAPL and CTCF performed in lysates from different $mESC^{WT}$, $mESC^{WAPL\text{-}AID}$ and $mESC^{WAPL/CTCF\text{-}AID}$ clones that were untreated or treated with IAA for 24 h. $mESC^{WT}$ were included to show the increased size of the tagged WAPL and CTCF compared to their endogenous versions. TUBULIN levels are shown as a loading control. (**E**) Images cropped out from representative images showing ZSCAN4 staining from the experiment shown in Fig. 1B. Scale bar: 50 µM. (**F**) Schematic representation of the targeting strategy used to generate $mESC^{WAPL\text{-}FKBP}$. (**G**) Western blot analysis of WAPL performed in lysates from different $mESC^{WAPL\text{-}FKBP}$ cell clones that were untreated or treated with dTAG for 24 h. $mESC^{WT}$ were included to show the increased size of the tagged WAPL protein. TUBULIN levels are shown as a loading control. (**H**) Western blot analysis of WAPL, CTCF and ZSCAN4 performed in lysates from a representative $mESC^{WAPL\text{-}FKBP}$ or $mESC^{WAPL\text{-}FKBP/CTCF\text{-}AID}$ clonal line untreated or treated with dTAG for the indicated times. TUBULIN levels are shown as a loading control. (**I**) Heatmaps generated from RNA-seq data from wild-type and DUX-expressing (right) or $mESC^{WAPL\text{-}AID}$ that were untreated and treated with IAA at different time points (left). DUX-expressing mESC were sorted in GFP+ or GFP- based on the activation of the LTR-GFP reporter. Heatmaps show the top 40 most upregulated genes upon DUX expression based on Cutoff Log2 > 1, *P* value < 0.05. (**J**) Plots showing normalized MERVL read counts obtained from RNA-seq datasets corresponding to untreated or IAA-treated $mESC^{WAPL\text{-}AID}$ and $mESC^{WAPL/CTCF\text{-}AID}$ samples. Data was obtained from Liu et al, 2021, 2025. *P* values are shown from one-tailed unpaired *t* tests. Data information: For (**E**, **G**, **H**), two independent experiments were performed but one representative experiment is shown. Source data are available online for this figure.

 © The Author(s)

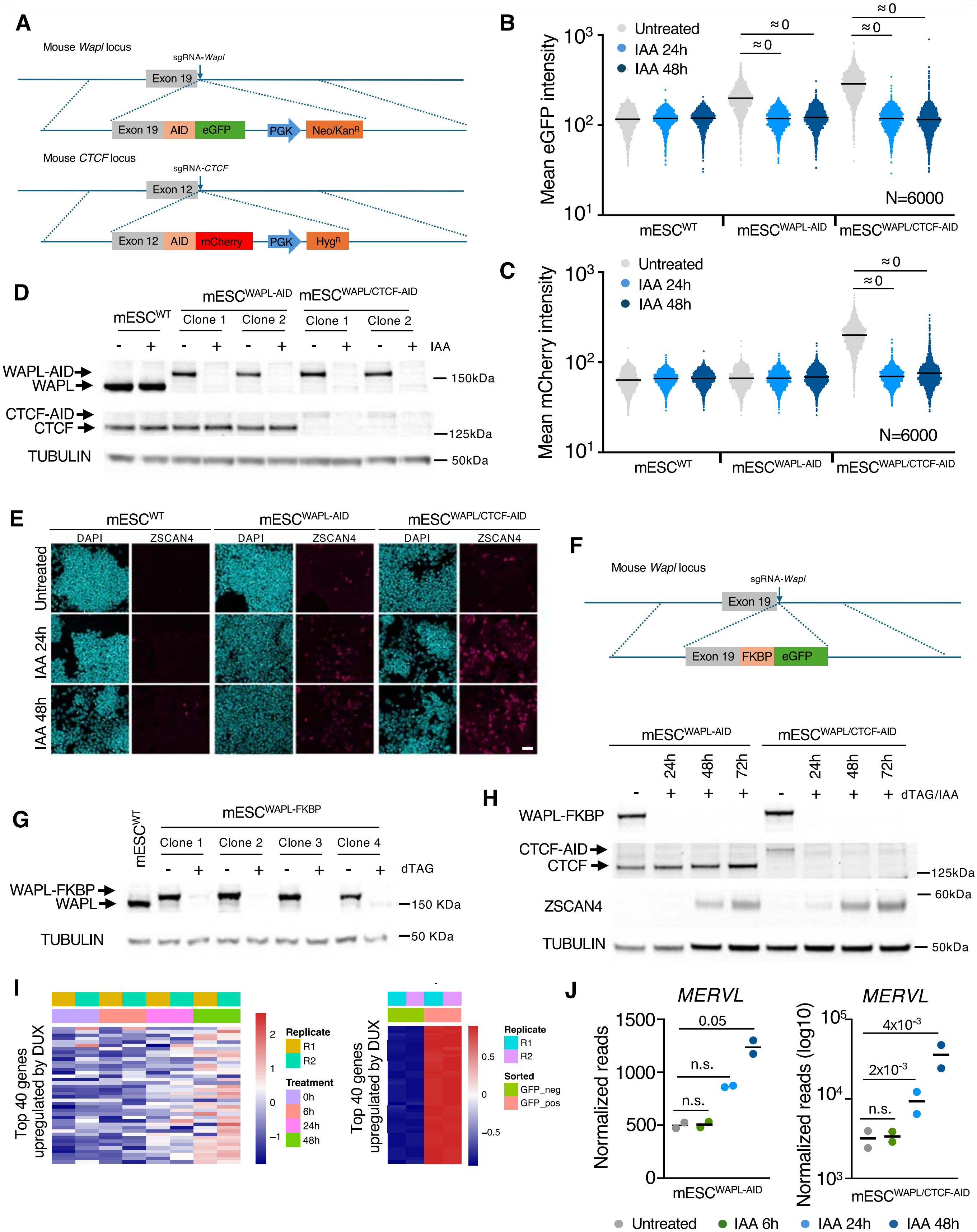
A
Mouse Wapl locus
sgRNA-Wapl
Exon 19
Exon 19 AID eGFP PGK Neo/KanR
Mouse CTCF locus
sgRNA-CTCF
Exon 12
Exon 12 AID mCherry PGK HygR
B
Mean eGFP intensity
Untreated
IAA 24h
IAA 48h
N=6000
mESCWT
mESCWAPL-AID
mESCWAPL/CTCF-AID
C
Mean mCherry intensity
D
WAPL-AID
WAPL
CTCF-AID
CTCF
TUBULIN
Clone 1
Clone 2
IAA
150kDa
125kDa
50kDa
E
DAPI
ZSCAN4
Untreated
IAA 24h
IAA 48h
F
Mouse Wapl locus
Exon 19 FKBP eGFP
G
mESCWAPL-FKBP
WAPL-FKBP
dTAG
150 KDa
50 KDa
H
dTAG/IAA
ZSCAN4
60kDa
I
Top 40 genes upregulated by DUX
Replicate
R1
R2
Treatment
0h
6h
24h
48h
Sorted
GFP_neg
GFP_pos
J
MERVL
Normalized reads
Normalized reads (log10)
IAA 6h
n.s.
0.05
4x10-3
2x10-3

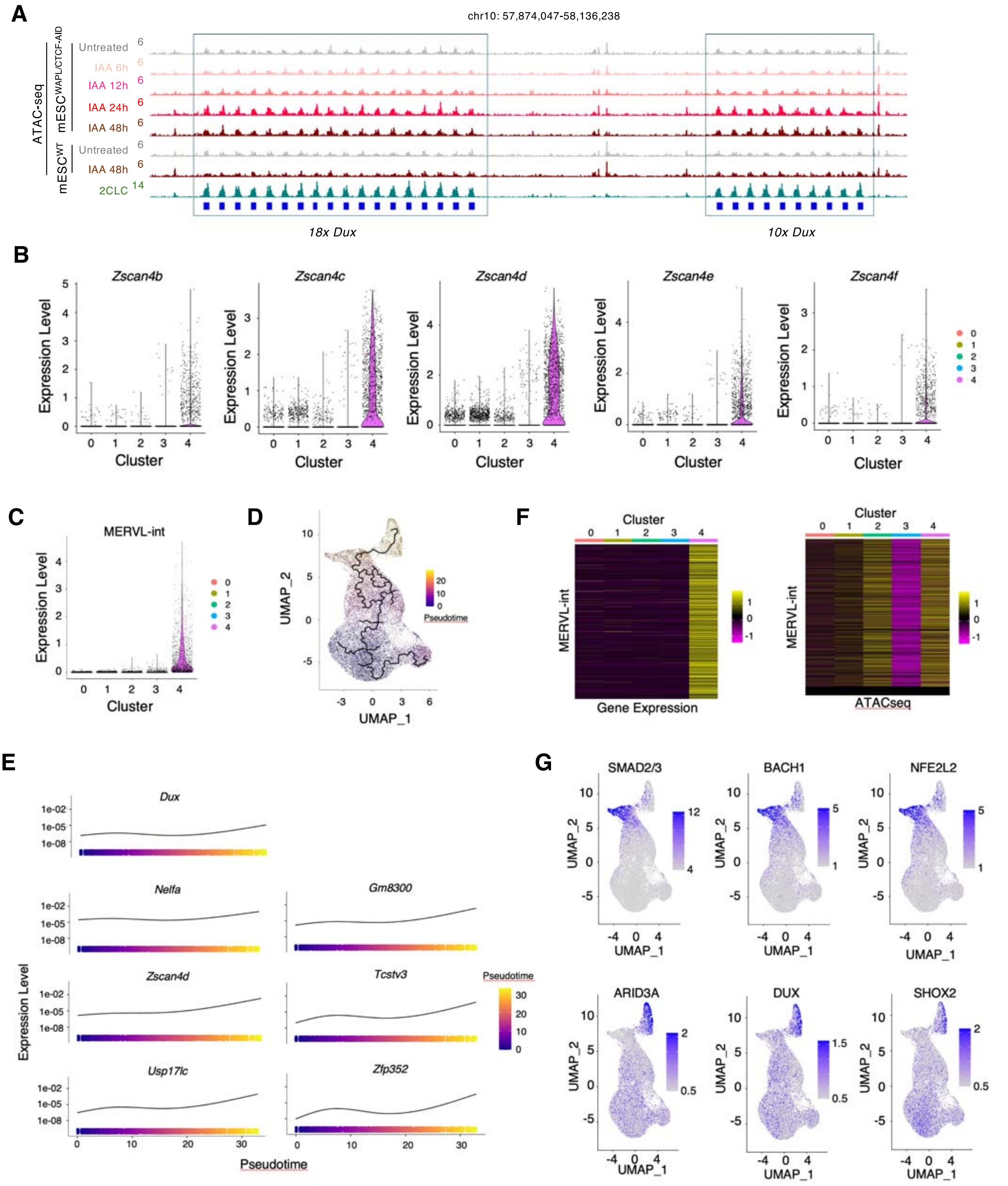
A
chr10: 57,874,047-58,136,238
ATAC-seq
mESC WAPL/CTCF-AID
Untreated
IAA 6h
IAA 12h
IAA 24h
IAA 48h
mESC WT
Untreated
IAA 48h
2CLC
18x Dux
10x Dux
B
Zscan4b
Zscan4c
Zscan4d
Zscan4e
Zscan4f
Expression Level
Cluster
C
MERVL-int
Expression Level
Cluster
D
UMAP_2
UMAP_1
Pseudotime
F
Cluster
MERVL-int
Gene Expression
ATACseq
E
Dux
Nelfa
Gm8300
Zscan4d
Tcstv3
Usp17lc
Zfp352
Expression Level
Pseudotime
G
SMAD2/3
BACH1
NFE2L2
ARID3A
DUX
SHOX2
UMAP_2
UMAP_1

© The Author(s)

Figure EV2. Supporting Fig. 2.

WAPL/CTCF depletion leads to transcriptional and chromatin accessibility changes over time. (**A**) Genome browser tracks showing ATAC-seq reads at the genomic region containing the *Dux* cluster of genes generated from $mESC^{WT}$ and $mESC^{CTCF/WAPL\text{-}AID}$ lines that were untreated and IAA-treated for the indicated times. Boxed are the two main regions containing 18 and 10 *Dux* gene copies. The 2CLC track shows ATAC-seq data generated from DUX-induced cells sorted based on positive LTR-RFP reporter expression. Tracks shown were derived from one $mESC^{WT}$ and one $mESC^{CTCF/WAPL\text{-}AID}$ clonal cell line. (**B**, **C**) Plots showing the expression level of the indicated genes (**B**) or MERVL elements (**C**) in individual cells from corresponding identified clusters. (Cluster 0, $n = 10123$; Cluster 1, $n = 6203$; Cluster 2, $n = 2697$; Cluster 3, $n = 1960$; Cluster 4, $n = 1444$). (**D**) UMAP visualization showing the inferred transcriptional trajectory (black line) over a colored pseudotime scale. (**E**) Plots showing the expression level of the indicated genes over a colored pseudotime scale. (**F**) Heatmaps showing the level of expression (right panel) and chromatin accessibility (left panel) in all annotated MERVL-int elements in all cell clusters. (**G**) UMAP visualization showing the transcription factor motif enrichment (top 3 motifs) characterizing cells from cluster 2 versus those from cluster 4 (upper panels) and vice versa (lower panels). Source data are available online for this figure.

© The Author(s)

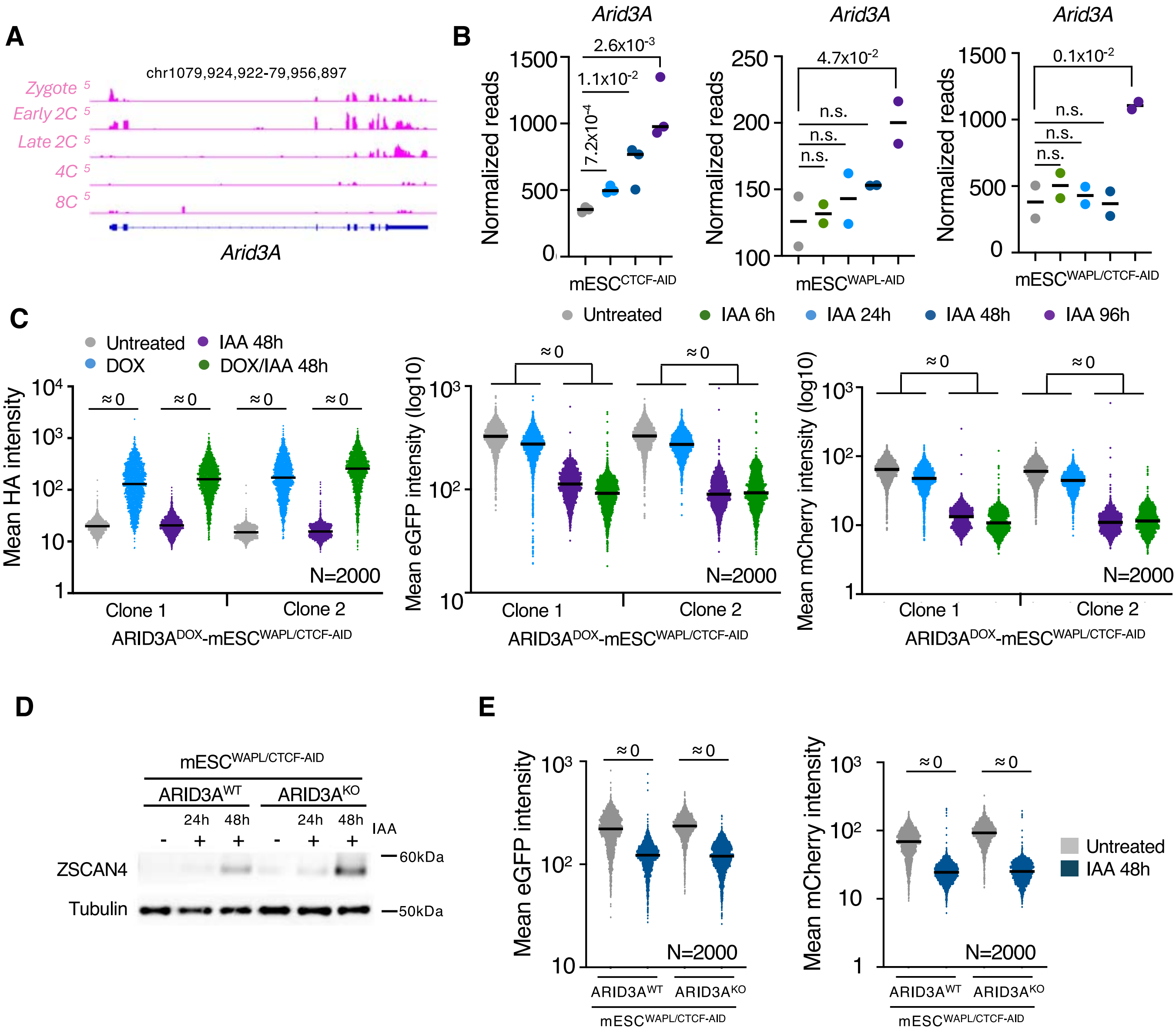

Figure EV3. Supporting Fig. 3.

ARID3A levels determine 2CLC conversion efficiency. (**A**) Genome browser tracks showing RNA-seq RPKM read count at *Arid3A* in samples obtained from Deng et al, 2014. (**B**) Plots showing normalized read counts obtained from RNA-seq datasets corresponding to untreated or IAA-treated for the indicated times $mESC^{CTCF\text{-}AID}$, $mESC^{WAPL\text{-}AID}$ and $mESC^{WAPL/CTCF\text{-}AID}$ samples. Data was obtained from Nora et al, 2017; Liu et al, 2021, 2025. *P* values are shown from one-tailed unpaired *t* tests. (**C**) High-throughput imaging quantification of HA (left panel) eGFP (center panel) and mCherry (right panel) mean nuclear intensity in $ARID3A^{DOX}$-$mESC^{WAPL/CTCF\text{-}AID}$ that were untreated or treated with DOX and/or IAA for 48 h. Center lines indicate mean values. *P* values are shown from one-tailed unpaired *t* tests. (**D**) Western blot analyses of ZSCAN4 performed in lysates from wild-type or ARID3A-deficient $mESC^{WAPL/CTCF\text{-}AID}$ untreated or treated with IAA for 48 h. TUBULIN levels are shown as a loading control. (**E**) High-throughput imaging quantification of eGFP (left panel) and mCherry (right panel) mean nuclear intensity in wild-type or ARID3A-deficient $mESC^{WAPL/CTCF\text{-}AID}$ that were untreated or treated with IAA for 48 h. Center lines indicate mean values. *P* values are shown from one-tailed unpaired *t* tests. Data information: For (**C**–**E**), two independent experiments were performed but only one representative is shown. Source data are available online for this figure.

 © The Author(s)

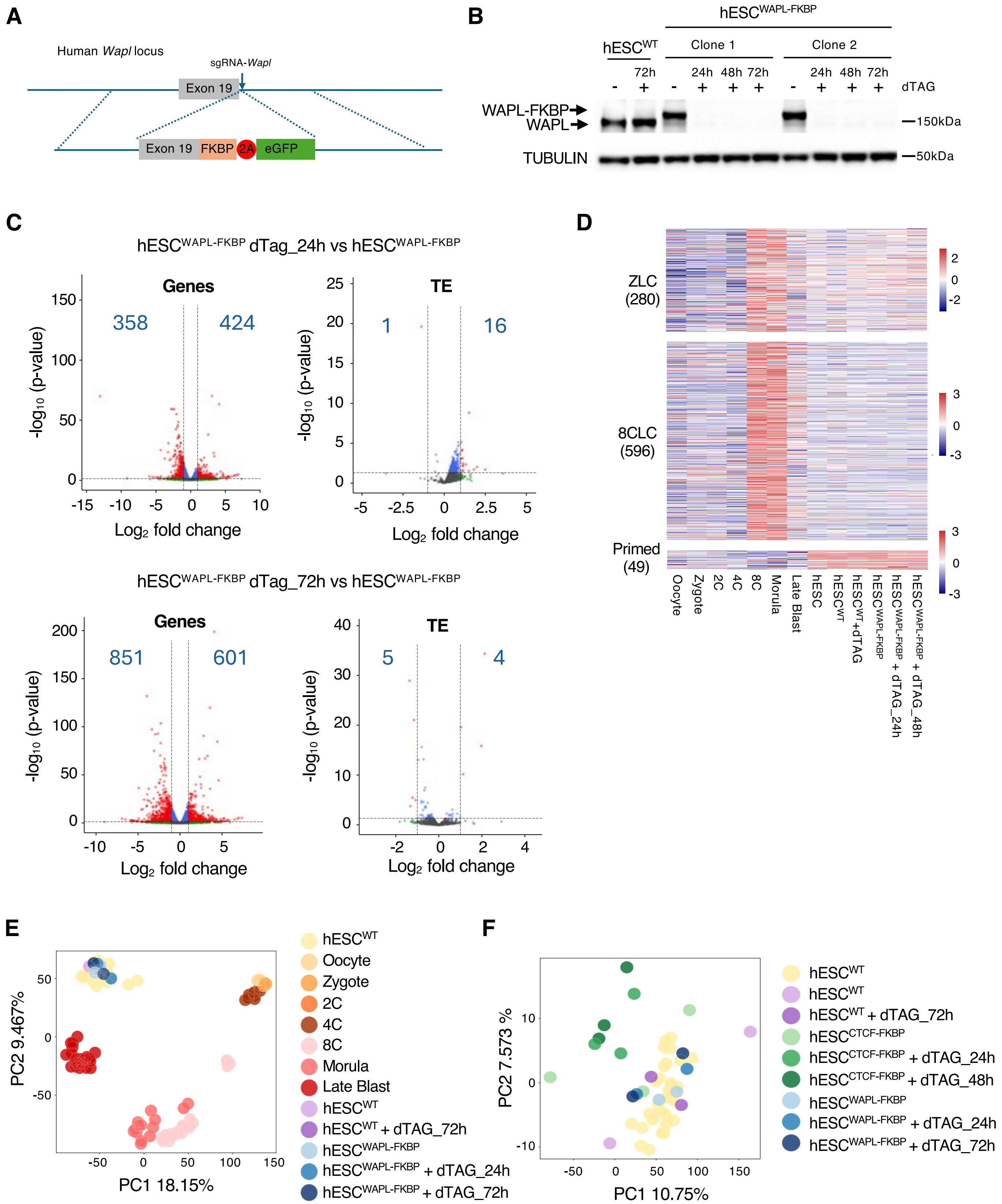
A
Human Wapl locus
sgRNA-Wapl
Exon 19
Exon 19 FKBP 2A eGFP
B
hESC^WAPL-FKBP
hESC^WT
Clone 1
Clone 2
72h
24h 48h 72h
24h 48h 72h
- + - + + + - + + +
dTAG
WAPL-FKBP
WAPL
150kDa
TUBULIN
50kDa
C
hESC^WAPL-FKBP dTag_24h vs hESC^WAPL-FKBP
Genes
358
424
-log10 (p-value)
Log2 fold change
TE
1
16
hESC^WAPL-FKBP dTag_72h vs hESC^WAPL-FKBP
Genes
851
601
TE
5
4
D
ZLC (280)
8CLC (596)
Primed (49)
Oocyte
Zygote
2C
4C
8C
Morula
Late Blast
hESC
hESC^WT
hESC^WT+dTAG
hESC^WAPL-FKBP
hESC^WAPL-FKBP + dTAG_24h
hESC^WAPL-FKBP + dTAG_48h
E
PC2 9.467%
PC1 18.15%
hESC^WT
Oocyte
Zygote
2C
4C
8C
Morula
Late Blast
hESC^WT
hESC^WT + dTAG_72h
hESC^WAPL-FKBP
hESC^WAPL-FKBP + dTAG_24h
hESC^WAPL-FKBP + dTAG_72h
F
PC2 7.573 %
PC1 10.75%
hESC^WT
hESC^WT
hESC^WT + dTAG_72h
hESC^CTCF-FKBP
hESC^CTCF-FKBP + dTAG_24h
hESC^CTCF-FKBP + dTAG_48h
hESC^WAPL-FKBP
hESC^WAPL-FKBP + dTAG_24h
hESC^WAPL-FKBP + dTAG_72h

© The Author(s)

Figure EV4. Supporting Fig. 4.

Loss of WAPL in hESC does not trigger expression of earlier transcriptional programs. (**A**) Schematic representation of the targeting strategy for tagging the human WAPL allele with an FKBP-2A-eGFP cassette. (**B**) Western blot analysis of WAPL performed in lysates from different $hESC^{WAPL\text{-}FKBP}$ cell clones that were untreated or treated with dTAG for 24, 48 and 72 h. $hESC^{WT}$ were included to show the increased size of the tagged WAPL protein. TUBULIN levels are shown as a loading control. Two independent experiments were performed but only one representative is shown. (**C**) Volcano plots showing genes (left panels) and TE (right panels) that are differentially expressed in $hESC^{WAPL\text{-}FKBP}$ that were untreated or treated with dTAG for 24 (upper panels) or 72 h (lower panels). Three independent cell clones were used in this analysis. Indicated are the number of differentially expressed genes and TE families. A Wald test was used to determine statistical significance. Thresholds are Log2 fold change>1 and *P* value < 0.01. (**D**) Heatmaps generated from the RNA-seq data shown in (**C**), including $hESC^{WT}$ that were untreated or treated with dTAG for 48 h as well as published RNA-seq datasets from single cells derived from different stages of human embryonic development. Heatmaps show subsets of genes enriched in ZLC (280 genes), 8CLC (596 genes) and in primed pluripotent cells (49 genes). (**E**) PCA plot including the same samples as in (**D**). (**F**) PCA plot including all $hESC^{WT}$, $hESC^{WAPL\text{-}FKBP}$ $hESC^{CTCF\text{-}FKBP}$ cell lines that were untreated and treated with dTAG for the indicated times. Source data are available online for this figure.

 © The Author(s)

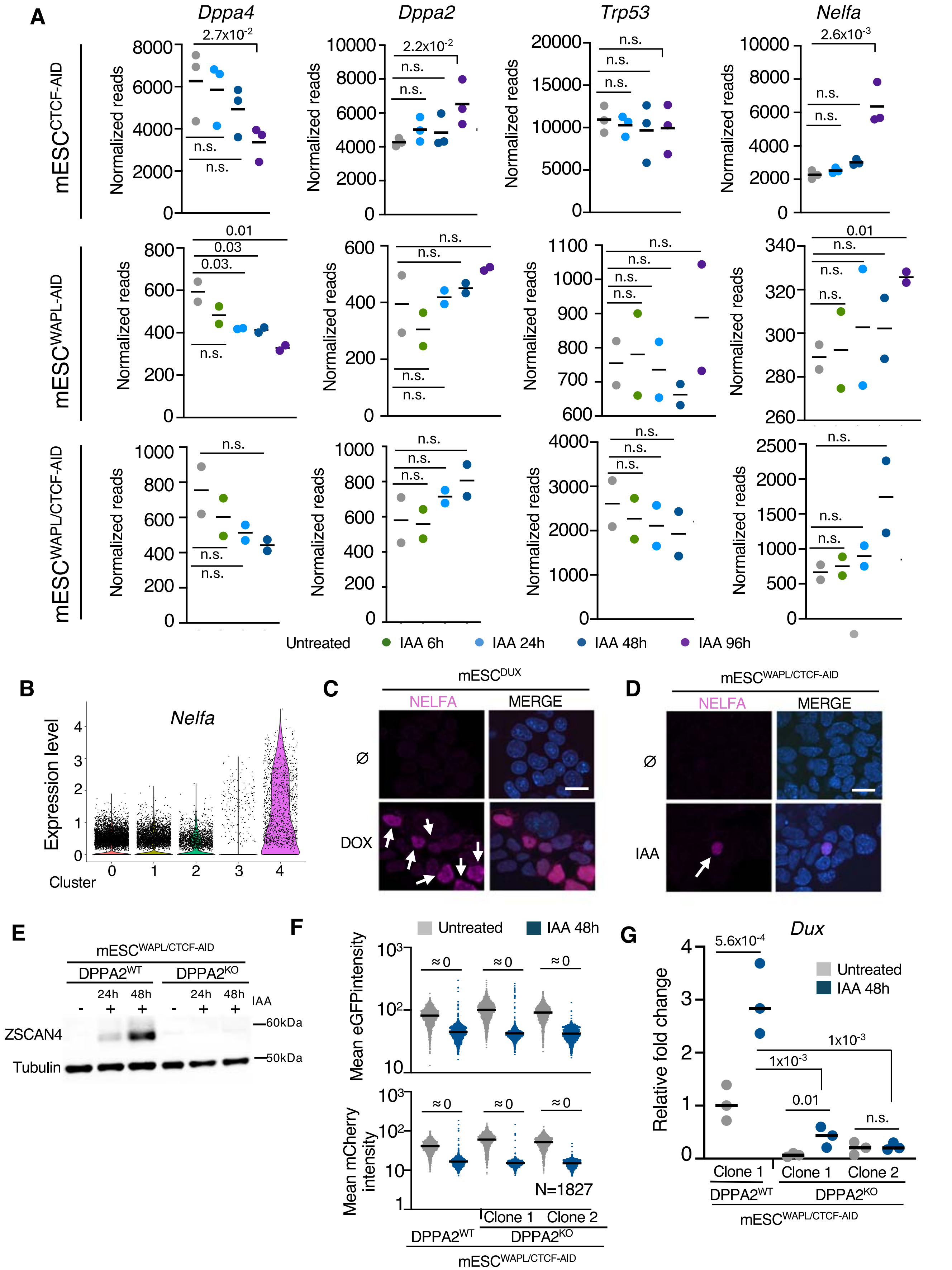
A
Dppa4
Dppa2
Trp53
Nelfa
mESC^CTCF-AID
mESC^WAPL-AID
mESC^WAPL/CTCF-AID
Normalized reads
Untreated
IAA 6h
IAA 24h
IAA 48h
IAA 96h
B
Nelfa
Expression level
Cluster
C
mESC^DUX
NELFA
MERGE
Ø
DOX
D
mESC^WAPL/CTCF-AID
NELFA
MERGE
Ø
IAA
E
mESC^WAPL/CTCF-AID
DPPA2^WT
DPPA2^KO
24h 48h 24h 48h
IAA
ZSCAN4
Tubulin
60kDa
50kDa
F
Untreated
IAA 48h
Mean eGFPintensity
Mean mCherry intensity
N=1827
Clone 1 Clone 2
DPPA2^WT
DPPA2^KO
mESC^WAPL/CTCF-AID
G
Dux
Relative fold change
Untreated
IAA 48h
5.6x10^-4
1x10^-3
1x10^-3
0.01
n.s.
Clone 1
Clone 1
Clone 2
DPPA2^WT
DPPA2^KO
mESC^WAPL/CTCF-AID

Figure EV5. Supporting Fig. 6.

DPPA2 is essential for 2CLC conversion upon CTCF/WAPL depletion. (**A**) Plots showing normalized read counts obtained from RNA-seq datasets corresponding to untreated or IAA-treated for the indicated times $mESC^{CTCF\text{-}AID}$, $mESC^{WAPL\text{-}AID}$ and $mESC^{WAPL/CTCF\text{-}AID}$ samples. Data was obtained from Nora et al, 2017; Liu et al, 2021 and Liu et al, 2025. (**B**) UMAP visualization showing expression levels of *Nelfa* in samples from Fig. 2C. (**C**, **D**) Representative confocal microscopy images showing representative NELFA staining in $mESC^{DUX}$ that were either untreated or DOX-treated for 16 h (**C**) and $mESC^{WAPL/CTCF\text{-}AID}$ that were either untreated or IAA treated for 48 h (**D**). Arrows indicate NELFA positive cells. DAPI was used to visualize nuclei. Scale bar: 10 µm. (**E**) Western blot analysis of ZSCAN4 performed in lysates from untreated or IAA-treated wild-type and DPPA2-deficient $mESC^{CTCF\text{-}AID}$ clones. TUBULIN levels are shown as a loading control. (**F**) High-throughput imaging quantification of eGFP (upper panel) and mCherry (lower panel) mean nuclear intensity in wild-type and *Dppa2*-deficient $mESC^{WAPL/CTCF\text{-}AID}$ that were untreated or treated with IAA for 48 h. Center lines indicate mean values. Two independent experiments were performed but only one representative is shown. For (**A**, **C**, **E**, **F**), *P* values are shown from one-tailed unpaired *t* tests. (**G**) Plot generated with real time-PCR analysis data showing the relative fold change expression of *Dux* in wild-type and *Dppa2*-deficient $mESC^{WAPL/CTCF\text{-}AID}$ that were untreated or treated with IAA for 48 h. *Gapdh* expression was used to normalize. Center lines indicate mean values. Two independent experiments were performed but only one representative experiment is shown. Source data are available online for this figure.

 © The Author(s)
