# Supplementary material for: Alterations in chromatin organization promote totipotent-like features in a DPPA2/DUX-dependent manner
Source: EMBO J. 2026 Jul 9;45(16):5657–88. doi: 10.1038/s44318-026-00853-6 (PMC13484482; doi:10.1038/s44318-026-00853-6)
Supplement: Supplementary file 3 [file 44318_2026_853_MOESM5_ESM.pdf]

THE EMBO JOURNAL

Alterations in chromatin organization promote totipotent-like features in a DPPA2/DUX-dependent manner

Grace Carey, Maria Vega-Sendino, Desiree Tillo, Sarah Hoelscher, Amir Khan, Ian Taukulis, Teresa Olbrich, Bechara Saykali, Andy Tran, Bhavya Soni, Anna Lee Fong, Melissa Mikolaj, Adam Harned, Kedar Narayan, Michael Kruhlak, Michael Kelly, Ningqing Liu, Elzo de Wit, and Sergio Ruiz

Corresponding author(s): Sergio Ruiz (sergio.ruizmacias@nih.gov)

Review Timeline:

Submission Date:	23rd Apr 25
Editorial Decision:	6th Jun 25
Appeal Received:	23rd Jun 25
Editorial Decision:	30th Jul 25
Revision Received:	26th Nov 25
Editorial Decision:	11th Feb 26
Revision Received:	17th Mar 26
Editorial Decision:	24th Apr 26
Revision Received:	29th Apr 26
Accepted:	10th Jun 26

Editor: Cornelius Schneider

Transaction Report:

(Note: With the exception of the correction of typographical or spelling errors that could be a source of ambiguity, letters and reports are not edited. Depending on transfer agreements, referee reports obtained elsewhere may or may not be included in this compilation. Referee reports are anonymous unless the Referee chooses to sign their reports.)

1st Editorial Decision **6th Jun 2025**

Dear Dr. Ruiz,

Thank you for submitting your manuscript for consideration by the EMBO Journal.

Your manuscript was sent to three referees and we have now received the comments of two of them. As the third reviewer has not yet returned his/her report and the other two are in fair agreement, I am taking a decision on your manuscript now, in order to save you from unnecessary delay. As you will see from the enclosed reports, neither referee supports publication of the manuscript in the EMBO Journal. I would prefer not to repeat their individual points of criticism in this letter, but both clearly point to a number of serious conceptual and technical shortcomings of your study, that, in our assessment, would preclude publication here.

Given these negative opinions and the fact that the EMBO Journal can only afford to accept papers which receive enthusiastic support from a majority of referees, we see little choice other to return the manuscript to you with the message that we cannot offer to publish it. I am sorry to have to disappoint you.

Yours sincerely,

Cornelius Schneider, PhD
Editor
The EMBO Journal
c.schneider@embojournal.org

Referee #1:

The manuscript submitted by Carey et al., 2025 addresses the possible contribution of chromatin structural elements to the conversion of mESCs to 2-cell like cells (2CLC). The article is an expansion and continuation of previous work from the lab (Olbrich et al., 2021), where the authors demonstrated the role played by CTCF as a barrier to 2CLC conversion through depletion of CTCF via the same system, CTCF-AID, used in the present study. Olbrich et al. also explored the role of Dux in this process and defined the Zscan4 cluster as a marker of 2CLC reprogramming. Here, Carey et al. reprise the CTCF-AID system and expand it by adding a DUXKO to confirm the role of Dux as a mediator of reprogramming. To strengthen the connection to chromatin remodeling, the authors add a WAPL-AID and double WAPL/CTCF-AID degron system and re-create some of the degron lines with an FKBP system rather than AID. Finally, they add a hESC model to investigate the 2CLC conversion in a different mammalian species.

Overall, the study tries to establish links between different aspects of genome organization and the process of conversion to a totipotency-like state. However, there are some major concerns, and the writing often lacks clarity, leaving the reader confused about some of the reasonings behind the experimental approaches and the data provided to support their claims.

Particularly troubling is a mismatch between text and information given in Extended data Figure 1a, where the text states that "the expression of these factors remains either unchanged or down-regulated up to 96 hours following CTCF depletion, suggesting they do not play a direct role in CTCF-mediated 2CLC conversion (Extended Data Figure 1a)", whereas the figure presents data from a WAPL-AID cell line, where no CTCF depletion was performed (source as stated by the authors: "RNAseq datasets26 corresponding to samples derived from mESCWAPL-AID"; the reference article only has WAPL-AID RNAseq).

There is also somewhat of a discrepancy between the content of the article and the prominent place given to Dux in its title. Upregulation of Dux is never strictly quantified, as neither Figure 1c not Extended Data 3b provide statistical analyses comparing Dux expression between IAA treated and non-treated cells for CTCF and WAPL degradation. This is perhaps because the upregulation is modest, especially in the CTCF depletion condition. Furthermore, no other attempt is made afterwards to understand how Dux upregulation may happen, with the authors presumably disregarding the hypothesis that it might be due to effects in cis rather than trans, and no mention of Dux afterwards.

More generally, the authors fail to provide statistical analysis for pairwise comparisons that are plotted in the figures. For example in Figure 1b only two comparisons are shown, whereas treated vs untreated should be provided in each case. This is important because the claim that "reprogramming is completely abolished in DUXKO mESCCTCF-AID, as evidenced by the lack of expression of 2C-associated markers" requires a strictly quantified comparison of treated and untreated cells. The same remark is applicable to several other panels in other figures as well.

The figures are constructed also in a way that is often confusing, as the same cell lines are sometimes labelled either "WT" or

through their full name in different panels of a same figure. For instance in Figure 1, mESCCTCF-AID are labelled as such in panels a, b, d, m, and n but are labelled as WT in panel c (as deducted by the figure legend and the text). Furthermore panels d, m and n also contain data from really WT mESCs, at which point most readers will be definitely lost. This is true of other figures as well.

The discussion of the data is also at times lacking in the text, as parts of figure panels are first ignored and then discussed later in the paper. This is the case of the double CTCF and WAPL degron cell line (Figure 2). Descriptions of markers or experiments are not properly provided, for example RFP intensity as a measure of MERVL activity, which will not be understood by someone outside the field. Another example is the Zscan4 gene cluster, where the appropriate description of the cluster itself is given for Figure 2b, but not for Figure 1c, where it is presented as a single gene marker rather than explained to be a combination of 6 genes.

Finally, some of the statements on chromatin regulation are overly simplistic. The most glaring example is in the discussion: "Importantly, while the loss of CTCF in mESC leads to TAD disruption, WAPL depletion results in altered cohesin turnover, which is important for maintaining promoter-enhancer loops." "While" is not correct, since CTCF is also an important mediator of enhancer-promoter loops (reviewed in Bower and Kvon, 2025).

Referee #2:

In the current study, the authors investigate the effects of deleting chromatin structure-related proteins, such as CTCF and WAPL, on the transition between pluripotency and totipotency using both mouse and human embryonic stem cell (ESCs) models. They show that in mESCs, loss of CTCF or WAPL promotes 2-cell-like cell (2CLC) reprogramming in a DUX-dependent manner, a phenomenon not observed in hESCs. The manuscript is well-structured, and the reseults are solid and comvincing. However, the study is primarily descriptive and lacks detailed investigation into the underlying molecular mechanisms. Elucidating these mechanisms would significantly strengthen the impact and scientific value of the work.

Major comments
My main concern with the manuscript is its limited novelty. The role of CTCF as a barrier to 2C-like reprogramming has been previously reported. Although the authors show that CTCF/WAPL depletion induces 2C-like cells in a DUX-dependent manner, DUX is a well-known and commonly cited factor in 2C induction, and thus does not offer sufficient mechanistic novelty. Moreover, the manuscript contains extensive descriptive characterization of phenotypes but lacks deeper mechanistic investigation. To improve the impact of the study, additional mechanistic insights should be explored, particularly through single-cell analyses. For example, integrative analysis of scRNA-seq and scATAC-seq data could be used to reveal transcriptional regulatory differences and distinct signaling pathways between successfully and unsuccessfully reprogrammed cells.

Minor comments
- What is the percentage of Zscan4$^+$/RFP$^+$ cells observed by FACS following CTCF/WAPL depletion?
- How does the chromatin state of 2CLCs in this study compare to that of bona fide 2-cell stage embryos?

** As a service to authors, EMBO Press provides authors with the possibility to transfer a manuscript that one journal cannot offer to publish to another EMBO publication or the open access journal Life Science Alliance launched in partnership between EMBO Press, Rockefeller University Press and Cold Spring Harbor Laboratory Press. The full manuscript and if applicable, reviewers' reports, are automatically sent to the receiving journal to allow for fast handling and a prompt decision on your manuscript. For more details of this service, and to transfer your manuscript please click on Link Not Available. **

Please do not share this URL as it will give anyone who clicks it access to your account.

Dear Cornelius, thank you for your email and the review of our manuscript. I must say that I feel shocked by these reviews and the decision to reject our work based on them. By reading the reviews very carefully, I do not think these revealed any major scientific, conceptual, or technical issues with our data. I understand they raised concerns, this is normal in a review, but neither of the reviewers explicitly recommended the rejection. Allow me to make a couple of points about it:

a) I am concerned about the review from reviewer 1 as I do not find a clear scientific concern. Most of the concerns from this reviewer refer to a figure that was mistakenly mislabeled (our fault), the lack of some statistical tests (which we did not include because we did not consider them essential to our points but we can easily add them), the confusion the reviewer has with the way some cell lines were labeled or the lack of a better description of the markers used in our manuscript for someone that is not from the field. He/she claims that "*no other attempt is made afterward to understand how Dux upregulation may happen, with the authors presumably disregarding the hypothesis that it might be due to effects in cis rather than trans, and no mention of Dux afterward*". I need to insist on this point: our data clearly show how nucleolar integrity, which is required to keep DUX loci silenced in the perinucleolar heterochromatin, is the mechanism driving 2CLC conversion (Figure 1). These data provide the mechanistic insight into the 2CLC conversion described in our work and I do not understand why the reviewer just disregarded it.

b) Reviewer 2 says that "The manuscript is well-structured, and the results are solid and convincing". However, this reviewer also argues that it "lacks detailed investigation into the underlying molecular mechanisms". I again disagree with this argument as we show how nucleolar integrity is the mechanism driving 2CLC conversion (Figure 1). I do not understand why this reviewer also ignored our proposed mechanism. I agree with the reviewer that "*DUX is a well-known and commonly cited factor in 2C induction*" but this is not our point. It was unknown its requirement to mediate totipotent-like conversion following alterations in chromatin architecture. This message is novel, and our findings will be definitively appreciated by the field. He/she suggests performing integrative analyses from scRNA and ATACseq data to reveal signaling pathways between successfully and unsuccessfully reprogrammed cells. We definitively can expand our analyses and provide new data we have in this regard.

In summary, because I feel that most of these concerns only require clarification, that our proposed mechanism was disregarded by both reviewers and that I am absolutely convinced we can address the remaining concerns, I urge you to reconsider your decision and wait for the report from the third reviewer. Give us a chance to convince you and the reviewers that our work deserves further consideration. I would not ask if I did not think we could completely satisfy these reviewers. If necessary, I can also provide you with a more specific experimental plan to address all the concerns from reviewers.

I am looking forward to hearing back from you,

Best wishes,

sergio

Dear Dr. Ruiz,

Thank you for submitting your manuscript for consideration by the EMBO Journal. It has been seen by two referees whose comments are shown below.

Thank you also for our productive discussions and for agreeing to perform experiments to provide additional insight. I would therefore like to invite you to submit a revised version of the manuscript, addressing the comments of all three reviewers as discussed in our meeting. I should add that it is EMBO Journal policy to allow only a single round of revision, and acceptance of your manuscript will therefore depend on the completeness of your responses in this revised version.

When preparing your letter of response to the referees' comments, please bear in mind that this will form part of the Review Process File, and will therefore be available online to the community. For more details on our Transparent Editorial Process, please visit our website: https://www.embopress.org/page/journal/14602075/authorguide#transparentprocess

We generally allow three months as standard revision time. As a matter of policy, competing manuscripts published during this period will not negatively impact on our assessment of the conceptual advance presented by your study. However, we request that you contact the editor as soon as possible upon publication of any related work, to discuss how to proceed. Should you foresee a problem in meeting this three-month deadline, please let us know in advance and we may be able to grant an extension.

Thank you for the opportunity to consider your work for publication. I look forward to your revision.

Yours sincerely,

Cornelius Schneider, PhD
Editor
The EMBO Journal
c.schneider@embojournal.org

Instructions for preparing your revised manuscript:

Please make sure you upload a letter of response to the referees' comments together with the revised manuscript.

Please also check that the title and abstract of the manuscript are brief, yet explicit, even to non-specialists.

When assembling figures, please refer to our figure preparation guideline in order to ensure proper formatting and readability in print as well as on screen:
https://bit.ly/EMBOPressFigurePreparationGuideline
See also guidelines for figure legends: https://www.embopress.org/page/journal/14602075/authorguide#figureformat

At EMBO Press we ask authors to provide source data for the main manuscript figures. You will receive a separate email with instructions for providing source data with your revised manuscript, including how to upload and organize the files.

IMPORTANT: When you send the revision we will require
- a point-by-point response to the referees' comments, with a detailed description of the changes made (as a word file).
- a word file of the manuscript text.
- individual production quality figure files (one file per figure)
- a complete author checklist, which you can download from our author guidelines (https://www.embopress.org/page/journal/14602075/authorguide).
- Expanded View files (replacing Supplementary Information)
Please see out instructions to authors
https://www.embopress.org/page/journal/14602075/authorguide#expandedview
- a Reagents and Tools Table as part of the Methods section, which can be downloaded from our author guidelines (https://www.embopress.org/page/journal/14602075/authorguide#structuredmethods)

Please remember: Digital image enhancement is acceptable practice, as long as it accurately represents the original data and conforms to community standards. If a figure has been subjected to significant electronic manipulation, this must be noted in the

figure legend or in the 'Materials and Methods' section. The editors reserve the right to request original versions of figures and the original images that were used to assemble the figure.

Further information is available in our Guide For Authors: https://www.embopress.org/page/journal/14602075/authorguide

We realize that it is difficult to revise to a specific deadline. In the interest of protecting the conceptual advance provided by the work, we recommend a revision within 3 months (28th Oct 2025). Please discuss the revision progress ahead of this time with the editor if you require more time to complete the revisions. Use the link below to submit your revision:

https://emboj.msubmit.net/cgi-bin/main.plex

POINT-BY-POINT RESPONSE TO THE REVIEWERS' COMMENTS

*We first would like to thank the reviewers for their comments, as we believe they have truly helped us to improve the first version of our manuscript (EMBOJ-2025-121165R-Q, **"Alterations in chromatin organization promote totipotent-like features in a DPPA2/DUX-dependent manner")**. In this revised version, we focused our efforts on consolidating our observations, clarifying the different concerns raised by the reviewers and providing substantial new data to expand our understanding of the molecular mechanisms driving 2CLC conversion following alterations in chromatin organization. In brief:*

1) *By mining our multiome analysis, we identified ARID3A as a transcription factor that limits the extent of 2CLC conversion following CTCF/WAPL-depletion (new Figure 3 and EV3).*

2) *We determined the essential role of the transcription factor DPPA2 as an upstream regulator of DUX in promoting 2CLC conversion following CTCF/WAPL-depletion (new Figure 6 and EV5).*

While we acknowledge that both reviewers raised concerns about our work, we hope that the addition of new data and analyses will satisfy these concerns. Please find below a point-by-point response to all the concerns and suggestions made by the reviewers.

Reviewer #1

Remarks to the Author:

The manuscript submitted by Carey et al., 2025 addresses the possible contribution of chromatin structural elements to the conversion of mESCs to 2-cell like cells (2CLC). The article is an expansion and continuation of previous work from the lab (Olbrich et al., 2021), where the authors demonstrated the role played by CTCF as a barrier to 2CLC conversion through depletion of CTCF via the same system, CTCF-AID, used in the present study. Olbrich et al. also explored the role of Dux in this process and defined the Zscan4 cluster as a marker of 2CLC reprogramming. Here, Carey et al. reprise the CTCF-AID system and expand it by adding a DUXKO to confirm the role of Dux as a mediator of reprogramming. To strengthen the connection to chromatin remodeling, the authors add a WAPL-AID and double WAPL/CTCF-AID degron system and re-create some of the degron lines with an FKBP system rather than AID. Finally, they add a hESC model to investigate the 2CLC conversion in a different mammalian species.

Overall, the study tries to establish links between different aspects of genome organization and the process of conversion to a totipotency-like state. However, there are some major concerns, and the writing often lacks clarity, leaving the reader confused about some of the reasonings behind the experimental approaches and the data provided to support their claims.

We understand the concern from the reviewer about the organization and the lack of clarity in some aspects of our manuscript. Thus, in this revised version, we reorganized the figures and rewrote the manuscript, hoping to provide better clarity and understanding for our experimental reasoning. We also added new data to further support our claims and provided new insights into the molecular mechanism driving totipotent-like conversion in our genetic cell models.

Particularly troubling is a mismatch between text and information given in Extended data Figure 1a, where the text states that "the expression of these factors remains either unchanged or down-regulated up to 96 hours following CTCF depletion, suggesting they do not play a direct role in CTCF-mediated 2CLC conversion (Extended Data Figure 1a)", whereas the figure presents data from a WAPL-AID cell line, where no CTCF depletion was performed (source as stated by the authors: "RNAseq datasets26 corresponding to samples derived from mESCWAPL-AID"; the reference article only has WAPL-AID RNAseq).

The reviewer is completely right on this point, and we sincerely apologize for the mistake. We had a mix-up with the data obtained from both CTCF and WAPL-depleted cell lines when we were preparing the figure. In this revised version, we fixed this problem and provided the right plots for all genetic conditions (Figure EV5A).

There is also somewhat of a discrepancy between the content of the article and the prominent place given to Dux in its title. Upregulation of Dux is never strictly quantified, as neither Figure 1c not Extended Data 3b provide statistical analyses comparing Dux expression between IAA treated and non-treated cells for CTCF and WAPL degradation. This is perhaps because the upregulation is modest, especially in the CTCF depletion condition. Furthermore, no other attempt is made afterwards to understand how Dux upregulation may happen, with the authors presumably disregarding the hypothesis that it might be due to effects in cis rather than trans, and no mention of Dux afterwards.

We understand the concern from the reviewer, but, in our opinion, Dux must have a prominent place in our manuscript. Indeed, we found that loss of DUX completely abolishes 2CLC reprogramming following CTCF or WAPL depletion, and this is one of the main claims from our work. To make this clearer to the reader, we reorganized the data and rewrote the manuscript to finish it with the data on the role of DUX in our genetic systems and the molecular mechanism underlying its requirement.
Regarding the expression levels of DUX, we now provide statistical analyses and added additional data to show that DUX expression increases following CTCF and/or WAPL depletion (Figure 5C, Figure EV5D, Appendix Figure S6A and Appendix Figure S7B). We agree with the reviewer on the fact that DUX upregulation is modest. This is something known in the field. For example, the original paper describing 2CLC (Macfarlan et al, Nature (2012) 487: 57-63) showed that DUX was upregulated 2.5-fold (microarray data) and 5.6-fold (RNAseq data) times in endogenous 2CLC compared to pluripotent ESC. However, in the same endogenous 2CLC,

ZSCAN4 was upregulated 130 times (RNAseq data). Independent of the extent of DUX upregulation, DUX is absolutely required for 2CLC conversion in our cell systems.
Finally, regarding the molecular mechanism underlying DUX upregulation, we proposed in our previous version that detachment of the Dux locus from perinuclear heterochromatin, because of altered nucleolar function, resulted in DUX expression and 2CLC conversion. Our proposed mechanism is supported by observations made by others on the role of the nucleolus controlling DUX expression (Yu et al, 2021; Xie et al, 2022) and our data showing altered nucleoli integrity (Figure 5D-N, Appendix Figure S6B-D). However, our data also suggested that Dux release from the PNH was likely not sufficient to trigger its expression. In fact, 2CLC conversion is not fully penetrant upon CTCF and/or WAPL depletion and additional factors might determine its reactivation. In this revised version, we now provide evidence that the transcription factor DPPA2 acts upstream of Dux and activates its expression. DPPA2, which has been shown to bind Dux promoter (Eckersley-Maslin et al, 2019), is expressed in wild-type pluripotent mESC but seems unable to access the Dux locus. We propose that following CTCF/WAPL depletion, alterations in nucleolar integrity might facilitate the release of Dux, enabling DPPA2 binding accessibility to the Dux promoter, activating its expression and promoting 2CLC conversion. In agreement, DPPA2-deficient cells do not express Dux or undergo 2CLC conversion upon CTCF/WAPL depletion (Figure 6).

More generally, the authors fail to provide statistical analysis for pairwise comparisons that are plotted in the figures. For example in Figure 1b only two comparisons are shown, whereas treated vs untreated should be provided in each case. This is important because the claim that "reprogramming is completely abolished in DUXKO mESCCTCF-AID, as evidenced by the lack of expression of 2C-associated markers" requires a strictly quantified comparison of treated and untreated cells. The same remark is applicable to several other panels in other figures as well.

We agree with the reviewer on this point, and we now provided statistical analyses between untreated and treated conditions in all panels.

The figures are constructed also in a way that is often confusing, as the same cell lines are sometimes labelled either "WT" or through their full name in different panels of a same figure. For instance in Figure 1, mESCCTCF-AID are labelled as such in panels a, b, d, m, and n but are labelled as WT in panel c (as deducted by the figure legend and the text). Furthermore panels d, m and n also contain data from really WT mESCs, at which point most readers will be definitely lost. This is true of other figures as well.

We understand the concern from the reviewer. We used multiple cell lines with different genetic modifications, and the naming might be difficult to follow. In this revised version, we simplified and homogenized the naming of all cell lines across all figures.

The discussion of the data is also at times lacking in the text, as parts of figure panels are first ignored and then discussed later in the paper. This is the case of the double CTCF and WAPL degron cell line (Figure 2). Descriptions of markers or experiments are not properly provided, for example RFP intensity as a measure of MERVL activity, which will not be understood by

someone outside the field. Another example is the Zscan4 gene cluster, where the appropriate description of the cluster itself is given for Figure 2b, but not for Figure 1c, where it is presented as a single gene marker rather than explained to be a combination of 6 genes.

We apologize for the lack of clarity. In this revised version, we reorganized the figures and rewrote the manuscript, hoping to provide a better clarity for readers outside of this field. We also hope that these changes help to understand all the markers and cell systems used in our manuscript.

Finally, some of the statements on chromatin regulation are overly simplistic. The most glaring example is in the discussion: "Importantly, while the loss of CTCF in mESC leads to TAD disruption, WAPL depletion results in altered cohesin turnover, which is important for maintaining promoter-enhancer loops." "While" is not correct, since CTCF is also an important mediator of enhancer-promoter loops (reviewed in Bower and Kvon, 2025).

The reviewer is right on this point and thus, we rewrote this sentence to be accurate.

Reviewer #2

Remarks to the Author:

In the current study, the authors investigate the effects of deleting chromatin structure-related proteins, such as CTCF and WAPL, on the transition between pluripotency and totipotency using both mouse and human embryonic stem cell (ESCs) models. They show that in mESCs, loss of CTCF or WAPL promotes 2-cell-like cell (2CLC) reprogramming in a DUX-dependent manner, a phenomenon not observed in hESCs. The manuscript is well-structured, and the results are solid and convincing. However, the study is primarily descriptive and lacks detailed investigation into the underlying molecular mechanisms. Elucidating these mechanisms would significantly strengthen the impact and scientific value of the work.

We appreciate the positive comments from the reviewer about our work. We also understand that our first version of the manuscript was primarily descriptive and warranted further investigation into the molecular mechanism underlying our observations. Thus, in this revised version, we expanded on the molecular mechanism proposed in our previous version: detachment of the Dux locus from perinuclear heterochromatin, because of altered nucleolar function, results in DUX expression and 2CLC conversion. Our proposed mechanism is supported by observations made by others on the role of the nucleolus controlling Dux expression (Yu et al, 2021; Xie et al, 2022) and our data showing altered nucleoli integrity (Figure 5D-N, Appendix Figure S6B-D). However, our data also suggested that Dux release from the PNH was likely not sufficient to trigger its expression. In fact, 2CLC conversion is not fully penetrant upon CTCF and/or WAPL depletion and additional factors might determine its reactivation. Here, we now provide evidence that the transcription factor DPPA2 acts upstream of Dux and activates its

expression. DPPA2, which has been shown to bind Dux promoter (Eckersley-Maslin et al, 2019), is expressed in wild-type pluripotent mouse ESC but seems unable to access the Dux locus. We propose that following CTCF/WAPL depletion, alterations in nucleolar integrity might facilitate the release of Dux, enabling DPPA2 binding accessibility to the Dux promoter, activating its expression and promoting 2CLC conversion. In agreement, DPPA2-deficient cells do not express Dux or undergo 2CLC conversion upon CTCF/WAPL depletion (Figure 6).
We believe that these observations significantly strengthen the impact of our work, provide new mechanistic insights and will be greatly appreciated by the field.

Major comments:

My main concern with the manuscript is its limited novelty. The role of CTCF as a barrier to 2C-like reprogramming has been previously reported. Although the authors show that CTCF/WAPL depletion induces 2C-like cells in a DUX-dependent manner, DUX is a well-known and commonly cited factor in 2C induction and thus does not offer sufficient mechanistic novelty. Moreover, the manuscript contains extensive descriptive characterization of phenotypes but lacks deeper mechanistic investigation. To improve the impact of the study, additional mechanistic insights should be explored, particularly through single-cell analyses. For example, integrative analysis of scRNA-seq and scATAC-seq data could be used to reveal transcriptional regulatory differences and distinct signaling pathways between successfully and unsuccessfully reprogrammed cells.

We agree with the reviewer on his/her statement: "DUX is a well-known and commonly cited factor in 2C induction". However, it was unknown its requirement to mediate totipotent-like conversion following alterations in chromatin structure. Indeed, our initial hypothesis was that this specific reprogramming was Dux-independent and rather due to alterations in enhancer/promoter dynamics because of alterations in the chromatin structure. Thus, our message is novel for the field, and our findings will be relevant to further understand the mechanisms underlying 2CLC conversion in mouse ESC. Nevertheless, we understand the concern from the reviewer and thus, we further investigated the molecular mechanism underlying Dux reactivation. We propose that following alterations in chromatin organization, defects in nucleolar integrity might facilitate the release of the Dux loci enabling the transcription factor DPPA2 binding accessibility to the Dux promoter, activating its expression and promoting 2CLC conversion (Fig. 5 and 6).
As suggested by the reviewer, we performed additional computational analysis on our single-cell data. Using Monocle, we identified modules of genes that change across time and are enriched in specific cell clusters (Appendix Fig. S4). For example, we determined that cells located in cluster 2, the cluster that is transcriptionally closer and located immediately prior to the 2CLC cluster in the pseudotime scale, are uniquely enriched in genes involved in cell adhesion or in the Hippo signaling pathway (Appendix Fig. S4). This suggests that genes controlling these processes and molecular pathways could influence successful 2CLC reprogramming. Future studies will determine whether this is the case.
Finally, by mining our single-cell data we identified ARID3A as a transcription factor that limits the extent of 2CLC conversion upon CTCF/WAPL-depletion (Figure 3). This finding is also novel

for the field and provides additional functional insights into the mechanism governing 2CLC reprogramming.
We think the addition of these new insights increase the relevance and scientific value of our work.
Minor comments:

- What is the percentage of Zscan4$^+$/RFP$^+$ cells observed by FACS following CTCF/WAPL depletion?

We were unable to analyze the percentage of RFP+ cells in CTCF/WAPL-depleted cells, as these cells contain eGFP and mCherry due to the gene targeting at the CTCF and WAPL alleles. In our lab, we only have LTR-based reporters expressing either GFP or Tomato, which will impose a conflict with the same fluorescent proteins expressed from endogenous CTCF and WAPL genes. Also, we did not specifically analyze the percentage of ZSCAN4+ cells by FACS as the data would be comparable to that obtained from high-throughput microscopy analyses, which were also performed at the single-cell level.

- How does the chromatin state of 2CLCs in this study compare to that of bona fide 2-cell stage embryos?

This is a great point made by the reviewer. We added additional density plots and histograms to our Appendix Figure 2 with ATACseq data from 2C stage embryos as well as the inner cell mass (ICM) samples. The ICM is the corresponding in vivo counterpart of mouse ESC and do not show ATACseq signal enrichment at MERVL elements or DUX-induced genes.

1st Revision - Editorial Decision 11th Feb 2026

Dear Dr. Ruiz,

Thank you for submitting your manuscript for consideration by the EMBO Journal. It has now been seen by three referees whose comments are shown below.

Thank you for re-submitting your revised manuscript (EMBOJ-2025-121165R1) to The EMBO Journal. The manuscript has now been evaluated by the two original referees, whose comments are provided below.

As you can see from the reports, both referees appreciate the revisions and agree that the manuscript has improved substantially. However, they also raise important remaining concerns that they feel would need to be addressed for publication in The EMBO Journal. We have now discussed these report within the team and decided that we will not require you to experimentally address point 1 and 2 of referee #2 but submit a revised manuscript addressing all other concerns raised by both referees.

When preparing your letter of response to the referees' comments, please bear in mind that this will form part of the Review Process File, and will therefore be available online to the community. For more details on our Transparent Editorial Process, please review our Editorial Policies: https://link.springer.com/partners/embo-press/editorial-policies

We generally allow three months as standard revision time. As a matter of policy, competing manuscripts published during this period will not negatively impact on our assessment of the conceptual advance presented by your study. However, we request that you contact the editor as soon as possible upon publication of any related work, to discuss how to proceed. Should you foresee a problem in meeting this three-month deadline, please let us know in advance and we may be able to grant an extension.

Thank you for the opportunity to consider your work for publication. I look forward to your revision.

Yours sincerely,

Cornelius Schneider, PhD
Editor
The EMBO Journal
c.schneider@embojournal.org

Instructions for preparing your revised manuscript:

Read our guidance for manuscript revisions and related editorial policies: https://link.springer.com/journal/44318/submission-guidelines#cms-Revised-submissions

Please make sure you upload a letter of response to the referees' comments together with the revised manuscript.

Please also check that the title and abstract of the manuscript are brief, yet explicit, even to non-specialists.

When assembling figures, please refer to our figure preparation guideline in order to ensure proper formatting and readability in print as well as on screen:
https://media.springernature.com/original/springer-cms/rest/v1/content/27825798/data/v1

At EMBO Press we ask authors to provide source data for the main manuscript figures. You will receive a separate email with instructions for providing source data with your revised manuscript, including how to upload and organize the files.

IMPORTANT: When you send the revision we will require
- a point-by-point response to the referees' comments, with a detailed description of the changes made (as a word file).
- a word file of the manuscript text.
- individual production quality figure files (one file per figure)
- a complete author checklist
- Expanded View files (replacing Supplementary Information)
- a Reagents and Tools Table as part of the Methods section

Please remember: Digital image enhancement is acceptable practice, as long as it accurately represents the original data and

conforms to community standards. If a figure has been subjected to significant electronic manipulation, this must be noted in the figure legend or in the 'Materials and Methods' section. The editors reserve the right to request original versions of figures and the original images that were used to assemble the figure.

We realize that it is difficult to revise to a specific deadline. In the interest of protecting the conceptual advance provided by the work, we recommend a revision within 3 months (12th May 2026). Please discuss the revision progress ahead of this time with the editor if you require more time to complete the revisions. Use the link below to submit your revision:

https://emboj.msubmit.net/cgi-bin/main.plex

Referee #1:

The authors have now presented a revised version of their manuscript, addressing some of the criticisms raised in original submission. The addition of the DPPA2 data is interesting and provides a mechanistic interpretation for DUX upregulation. However, some of the data still requires clarification.
Major concern: More evidence should be provided for the choice of Dppa2 over Nelfa as main regulator of Dux, since the two genes seem to have relatively similar levels of expression in untreated cells so could both potentially activate Dux (Fig EV5 A). The authors claim that: "Whereas Nelfa is only detected in cells from cluster 4, corresponding to 2CLC (Fig. 2F), DPPA2 is expressed in cells from all clusters and further enriched in cluster 4 (Fig. 6A,B)". Figure 2F is difficult to visualise but there seems to be Nelfa expressing cells in all clusters and timepoints, not just in cluster 4 as claimed. This difference seems quite clear when comparing to the MERVL-int panel in the same Fig. 2F. To strengthen their claim authors should provide cluster-based and time-point based expression for Nelfa, as done for Dppa2 in Fig. 6B from the scRNAseq data and comparison between the two genes. Importantly, the authors disregard the possibility that there could be some level of cooperation in the activation of Dux by these two genes, as evidence for the small but significant upregulation of Dux in Clone 1 DPPA2-KO (Fig. EV5 D).
Another major point: In the scATAC-seq, the authors talk about alterations in chromatin accessibility and examination of chromatin status of "proximal regions" of differentially expressed genes, stating that "alterations to chromatin accessibility in gene regulatory regions following CTCF/WAPL depletion could lead to activation of 2C-associated transcripts." However, the corresponding Fig. 2J is labelled as: "Heatmap showing the set of genes differentially expressed in cluster 4 (left) and the corresponding level of chromatin accessibility of the same genes in all clusters (right)." The two statements seem at odds, as the main text claims to be analysing "proximal regions" and "regulatory regions", which should not be the gene bodies themselves. Therefore, 1. The nature of these proximal regions should be clearly stated and their proximity to TSS should also be specified. 2. Other potential annotations should be provided: are some of these regions promoters? annotated enhancers? putative enhancers? Finally and importantly, how these regions are identified and associated to a gene needs to be included in the Methods section.
Finally, two minor points:
1. Figure 3D shows +Dox/IAA treatment vs IAA only as labelled in the figure, so Dox treatment vs no Dox. Shouldn't the associated volcano plot be switched? Addition of Dox should upregulate ARID3A, not downregulate it. Same goes for the TE panel.
2. Figure EV5 A, the scaling should be uniform between all panels and experiments, whereas it is currently a mixture of log10, log2 or non-transformed normalised reads without a clear logic to why some are transformed and some are not.

Referee #2:

Report for Author:
In the current study, the authors have investigated the effects of deleting chromatin structure-related proteins, including CTCF and WAPL, on the transition between mouse embryonic stem cells and two-cell like cells using both mouse and human embryonic stem cell (ESCs) models. They demonstrated that in mESCs, loss of CTCF or WAPL promotes 2-cell-like cell (2CLC) reprogramming in a DUX-dependent manner, a phenomenon not observed in hESCs. As suggested, the author performed the scRNA-seq and scATAC-seq to look into the mechanism behind. Mechanistic studies showed ARID3A could function as a barrier during the 2C transition. Furthermore, CTCF loss leads to DUX away from heterochromatin hub, thereby becoming more accessible and being activated by DPPA3. The manuscript is of interest, and the results are solid and convincing. However, a few issues and confusions should be addressed before it is suitable for publication.

Major comments
- Although the authors tried to investigate the mechanisms underlying 2C induction following CTCF/WAPL loss, the mechanistic framework appears insufficiently integrated. For example, the molecular role of ARID3A during the 2C transition is not clearly defined, and it remains unclear how ARID3A overexpression affects Dux activation. Greater emphasis should be placed on elucidating how ARID3A regulates the 2C transition, particularly in the context of Dux inactivation upon CTCF/WAPL depletion.

- The authors showed that CTCF depletion leads to the release of Dux loci from repressive PNH. I am wondering whether a similar relocalization occurs upon WAPL depletion. if not, what could be the reason? The underlying reasons for this discrepancy should be addressed, and the discordant effects of CTCF versus WAPL loss should be discussed in greater detail.
- The authors have proposed that the release of Dux loci from PNH could facilitate binding accessibility of Dux to transcriptional activators, thereby activating Dux transcription. However, this conclusion is primarily supported by transcriptome analyses from a Dppa2 knockout model. In my view, including chromatin profiling data, such as ChIP-seq or CUT&Tag analysis, to directly assess changes in DPPA2 occupancy at the Dux locus would provide more convincing mechanistic evidence.

Minor comments
- According to gene nomenclature guidelines, mouse gene names should be written in italics with the first letter capitalized, whereas human genes should be written in italics with all letters capitalized. Protein names for both mouse and human genes should be written with capital letters. Please ensure consistency throughout the manuscript.
- In Figure 1B, the authors presented quantification of Zscan4-positive cells based on imaging data. It would be helpful to include representative cell images to complement the quantitative analysis.
- In Figures 5E-L, several labels are missing within the Figures, which makes the images difficult to interpret. Please add appropriate labels for clarity.

POINT-BY-POINT RESPONSE TO THE REVIEWERS' COMMENTS

*We first would like to thank the reviewers for their final comments on our manuscript, as we believe they have truly helped us to refine our manuscript (EMBOJ-2025-121165R1, **"Alterations in chromatin organization promote totipotent-like features in a DPPA2/DUX-dependent manner")**. In this revised and final version, we clarified the remaining concerns raised by the reviewers and added new data to better understand the molecular mechanisms driving 2CLC conversion following alterations in chromatin organization.*

1) *To address Reviewer #1, we provided more evidence and reasoning over our choice to investigate further the role of DPPA2, and we clarified the nature of the proximal regions examined in Figure 2J as well as updated our Methods.*

2) *To address Reviewer #2, we performed ChIPseq analyses to determine DPPA2 binding to the DUX cluster following CTCF/WAPL depletion (new Figure 6G).*

Please find below a point-by-point response to all the concerns and suggestions made by the reviewers.

Reviewer #1

Remarks to the Author:

The authors have now presented a revised version of their manuscript, addressing some of the criticisms raised in original submission. The addition of the DPPA2 data is interesting and provides a mechanistic interpretation for DUX upregulation. However, some of the data still requires clarification.

Major concern: More evidence should be provided for the choice of Dppa2 over Nelfa as main regulator of Dux, since the two genes seem to have relatively similar levels of expression in untreated cells so could both potentially activate Dux (Fig EV5 A). The authors claim that: "Whereas Nelfa is only detected in cells from cluster 4, corresponding to 2CLC (Fig. 2F), DPPA2 is expressed in cells from all clusters and further enriched in cluster 4 (Fig. 6A,B)". Figure 2F is difficult to visualize but there seems to be Nelfa expressing cells in all clusters and timepoints, not just in cluster 4 as claimed. This difference seems quite clear when comparing to the MERVL-int panel in the same Fig. 2F. To strengthen their claim authors should provide cluster-based and time-point based expression for Nelfa, as done for Dppa2 in Fig. 6B from the scRNAseq data and comparison between the two genes. Importantly, the authors disregard the possibility that there could be some level of cooperation in the activation of Dux by these two genes, as evidence for the small but significant upregulation of Dux in Clone 1 DPPA2-KO (Fig. EV5 D).

We understand the concern from the reviewer and agreed that further clarification on our choice to investigate DPPA2 over NELFA should be provided. The reviewer is right about the similar RNA

expression levels for both Dppa2 and Nelfa in untreated cells shown in Fig. EV5A. Moreover, this is also consistent with a UMAP plot for Nelfa (new Fig. EV5B) similar to the one previously shown for Dppa2 (Fig. 6B). However, we performed additional immunostaining analyses for NELFA and, while DPPA2 protein expression is ubiquitous in untreated cells (Fig. 6C,D), NELFA protein expression is negligible in untreated ESC and can only be detected following CTCF/WAPL-depletion or DUX expression (new Fig. EV5C,D). Similar results for Nelfa expression in ESC were also previously reported (Hu et al, 2020). Because some Nelfa RNA expression can be detected in cells from some clusters different from the cluster containing 2CLC (cluster 4), these observations suggest that post-transcriptional regulation could be in place in ESC to limit the levels of NELFA protein. More importantly, recent work has suggested that NELFA could be a marker rather than a driver of the 2CLC state (Grow et al, 2021). For all the above reasons, we decided to focus on the role of DPPA2 as it is already expressed in untreated mESC$^{WAPL/CTCF\text{-}AID}$ and could influence 2CLC reprogramming at different steps during the process. We discussed all this in the revised version. We would also like to clarify that we did not disregard in our manuscript the possibility that NELFA could potentially have a cooperative role in activating DUX expression. The reviewer suggested that this could be the case based on the results from Figure EV5D. However, the slight upregulation of Dux expression in Clone 1 did not reach the levels observed in untreated ESC, suggesting that, if NELFA plays a role in activating Dux expression, it seems minor. Furthermore, as mentioned above, recent work suggested that NELFA could be a marker rather than a driver of the 2CLC state (Grow et al, 2021).

Another major point: In the scATAC-seq, the authors talk about alterations in chromatin accessibility and examination of chromatin status of "proximal regions" of differentially expressed genes, stating that "alterations to chromatin accessibility in gene regulatory regions following CTCF/WAPL depletion could lead to activation of 2C-associated transcripts." However, the corresponding Fig. 2J is labelled as: "Heatmap showing the set of genes differentially expressed in cluster 4 (left) and the corresponding level of chromatin accessibility of the same genes in all clusters (right)." The two statements seem at odds, as the main text claims to be analysing "proximal regions" and "regulatory regions", which should not be the gene bodies themselves. Therefore, 1. The nature of these proximal regions should be clearly stated and their proximity to TSS should also be specified. 2. Other potential annotations should be provided: are some of these regions promoters? annotated enhancers? putative enhancers? Finally and importantly, how these regions are identified and associated to a gene needs to be included in the Methods section.

The reviewer is completely right on this point, and we apologize for the implications of our wording. We removed the word “regulatory” because, as the reviewer pointed out, it is unknown whether the chromatin regions we show in Fig. 2J are regulatory. For this revised version, we rewrote the section to clarify how the analysis was performed and included the methodological details in the Methods. To generate the heatmaps, we first filtered the chromatin regions that were differentially accessible in cells from clusters 4 (2CLC) and 2 (cell cluster immediately prior to cluster 4 in the pseudotime scale) compared to the rest of the clusters and assigned those to the nearest genomic feature using the “ClosestFeature” function in Signac. Basically, the “ClosestFeature” function identifies the nearest genomic feature (usually a gene, exon or TSS) for

a given set of ATACseq peaks. It links physical accessibility to the closest gene, and likely target, by providing the distance between the center of the ATACseq peak and the nearest genomic feature. The primary genomic features associated with a gene are open chromatin peaks (specifically, peak-based gene activity scores), which include the gene body and its promoter regions. Thus, this function suggests potential target genes associated with open chromatin peaks. Table EV4 highlights the gene features ('closest region') and their nature (type) associated with filtered ATACseq peaks from cells from clusters 2 and 4 ('region'), as well as the distance to the feature ('distance').
Additionally, we overlapped the set of ATACseq peaks used in this analysis with all transcription start sites (TSS) as well as with a set of reported mouse ESC enhancers (Hnisz, D., et al. Cell. 2013 155:934-47; Whyte, W.A., et al, Cell. 2013 153:307-19). This analysis showed that most of the newly opened chromatin regions following CTCF/WAPL depletion in cells from clusters 2 and 4, do not majorly overlap with TSS (<5%) and have negligible overlap with known ESC enhancers (<1%) (Figure R1 below). This suggests that CTCF/WAPL depletion promotes changes in chromatin accessibility of potential new regulatory sequences that are associated with genes that show transcriptional changes. Future studies will need to determine the regulatory nature of this set of chromatin regions and their specific relevance to 2CLC conversion.

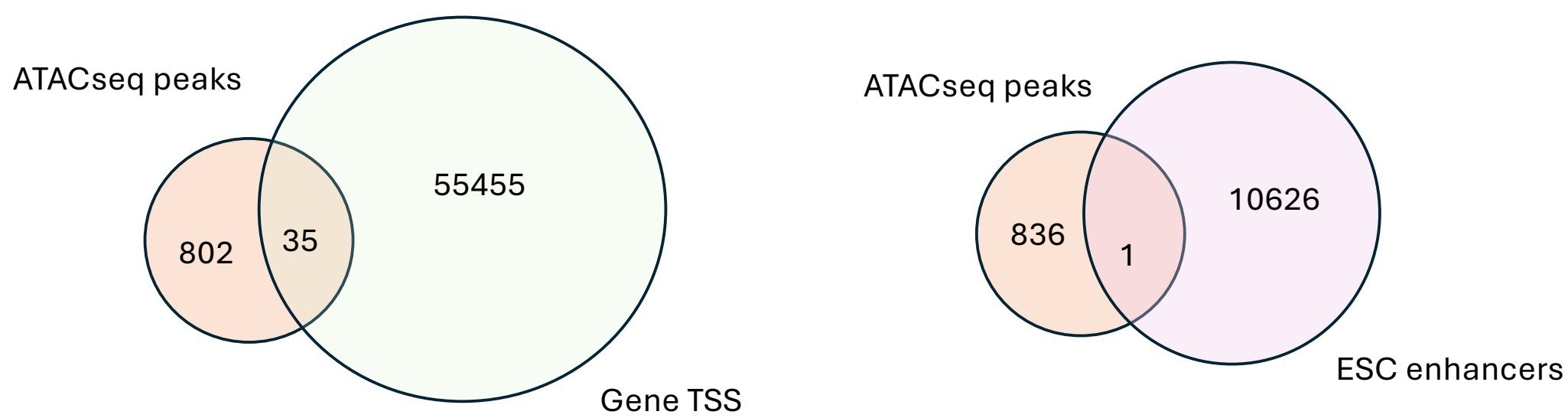

Figure R1: Venn diagrams showing the overlap between ATACseq peaks differentially accessible in clusters 2 and 4 and all gene TSS (right) and mouse ESC enhancers (left).

Finally, two minor points:

1. Figure 3D shows +Dox/IAA treatment vs IAA only as labelled in the figure, so Dox treatment vs no Dox. Shouldn't the associated volcano plot be switched? Addition of Dox should upregulate ARID3A, not downregulate it. Same goes for the TE panel.

The reviewer is completely right, and we apologize for the mistake. We have now corrected the panel from Figure 3D.

2. Figure EV5 A, the scaling should be uniform between all panels and experiments, whereas it is currently a mixture of log10, log2 or non-transformed normalized reads without a clear logic to why some are transformed and some are not.

The reviewer is completely right, and we uniformed all plots from Fig. EV5A.

Reviewer #2

Remarks to the Author:

In the current study, the authors have investigated the effects of deleting chromatin structure-related proteins, including CTCF and WAPL, on the transition between mouse embryonic stem cells and two-cell like cells using both mouse and human embryonic stem cell (ESCs) models. They demonstrated that in mESCs, loss of CTCF or WAPL promotes 2-cell-like cell (2CLC) reprogramming in a DUX-dependent manner, a phenomenon not observed in hESCs. As suggested, the author performed the scRNA-seq and scATAC-seq to look into the mechanism behind. Mechanistic studies showed ARID3A could function as a barrier during the 2C transition. Furthermore, CTCF loss leads to DUX away from heterochromatin hub, thereby becoming more accessible and being activated by DPPA3. The manuscript is of interest, and the results are solid and convincing. However, a few issues and confusions should be addressed before it is suitable for publication.

Major comments:

- Although the authors tried to investigate the mechanisms underlying 2C induction following CTCF/WAPL loss, the mechanistic framework appears insufficiently integrated. For example, the molecular role of ARID3A during the 2C transition is not clearly defined, and it remains unclear how ARID3A overexpression affects Dux activation. Greater emphasis should be placed on elucidating how ARID3A regulates the 2C transition, particularly in the context of Dux inactivation upon CTCF/WAPL depletion.

While we understand the interest of the reviewer in further investigating the molecular role of ARID3A in 2CLC transition, the initial request was to use our scRNAseq/ATACseq data to reveal "transcriptional regulatory differences and distinct signaling pathways between successfully and unsuccessfully reprogrammed cells". In our view, we addressed the initial concern. We used our scRNAseq/ATACseq dataset to identify ARID3A as a novel regulator of 2CLC conversion upon CTCF/WAPL depletion and provided strong evidence, as well as a new main Figure, to support this claim. Although we share the interest shown by the reviewer to understand mechanistically how ARID3A regulates the 2C transition, we find this request out of the scope of our manuscript. Our work is not about the role of ARID3A in 2CLC conversion and, addressing this point, will require substantial experimental work that we find unnecessary to support the main claims of our work. We plan to explore this in the future, but we find unrealistic to perform these experiments for this manuscript. We hope the reviewer understands our point.

- The authors showed that CTCF depletion leads to the release of Dux loci from repressive PNH. I am wondering whether a similar relocalization occurs upon WAPL depletion. if not, what could be the reason? The underlying reasons for this discrepancy should be addressed, and the discordant effects of CTCF versus WAPL loss should be discussed in greater detail.

We also understand the interest of the reviewer to address whether WAPL depletion leads to similar release of Dux loci from repressive PNH. However, we find this comment unfair at this point in the review process. This new request is based on data that was included in our first version of the manuscript. However, this was not a concern during the first round of revision. Our data suggest that indeed, WAPL depletion might lead to a similar release of the Dux loci from PNH. This is supported by our data showing the absence of 2CLC conversion in WAPL-depleted DUX-KO ESC lines. Addressing this point will also require substantial experimental work that we find unfair at this stage of the review process. We also hope the reviewer understands our point.

- The authors have proposed that the release of Dux loci from PNH could facilitate binding accessibility of Dux to transcriptional activators, thereby activating Dux transcription. However, this conclusion is primarily supported by transcriptome analyses from a Dppa2 knockout model. In my view, including chromatin profiling data, such as ChIP-seq or CUT&Tag analysis, to directly assess changes in DPPA2 occupancy at the Dux locus would provide more convincing mechanistic evidence.

This is a great point made by the reviewer. For this revised version, we performed a ChIPseq experiment to examine DPPA2 chromatin enrichment in untreated or IAA-treated mESC$^{WAPL/CTCF\text{-}AID}$. Interestingly, we found some DPPA2 pre-bound to all Dux gene copies of the cluster in untreated ESC besides the lack of Dux expression and the little chromatin accessibility observed at the gene cluster (new Figs. 6G and EV2A). Importantly, CTCF/WAPL depletion leads to increased DPPA2 occupancy and chromatin accessibility at all DUX gene copies (new Fig. 6G and EV2A). DPPA2 lacks any known enzymatic activity and has been suggested to be an epigenetic priming factor as its binding could be required for future activation upon specific signals or transcription factors (Eckersley-Maslin, 2020). Future studies will be needed to determine the precise role of DPPA2 enrichment at the Dux gene cluster. Nevertheless, our findings suggest a model where the loss of CTCF/WAPL leads to the release of the Dux loci from the repressive PNH environment, enabling further DPPA2 enrichment at the Dux gene copies and facilitating Dux expression in a DPPA2-dependent manner to promote 2CLC conversion.

Minor comments:

- According to gene nomenclature guidelines, mouse gene names should be written in italics with the first letter capitalized, whereas human genes should be written in italics with all letters capitalized. Protein names for both mouse and human genes should be written with capital letters. Please ensure consistency throughout the manuscript.

We reviewed the manuscript and ensured consistency when naming genes and proteins.

- In Figure 1B, the authors presented quantification of Zscan4-positive cells based on imaging data. It would be helpful to include representative cell images to complement the quantitative analysis.

We have now included representative images from the experiment shown in Figure 1B (new Fig. EV1E).

- In Figures 5E-L, several labels are missing within the Figures, which makes the images difficult to interpret. Please add appropriate labels for clarity.

We are not sure what labels the reviewer is referring to. We added information about the meaning of the numbers we included in our images. We are happy to add/modify these panels accordingly if further needed.

Dear Dr. Ruiz,

Thank you for submitting a revised version of your manuscript. The study has now been evaluated by the original referee #1, who considers that their previous concerns have been satisfactorily addressed and now recommends publication.
We were unfortunately unable to obtain feedback from referee #2 and have therefore decided to proceed based on the report from referee #1.
There remain a few mainly editorial points that will need to be addressed before I can proceed with formal acceptance of the manuscript:

- FUNDING INFO: missing in eJP: FNLCR Contract 75N91019D0024

- As we are switching from a free-text author contribution statement towards a more formal statement based on Contributor Role Taxonomy (CRediT) terms, please remove the present Author Contribution section and instead specify each author's contribution(s) directly in the Author Information page of our submission system during upload of the final manuscript. See https://casrai.org/credit/ for more information.

- DATASET EV LEGENDS: source file names, titles, legends and manuscript callouts all need to be updated to Dataset EV1-EV10 instead of EV1-EV4 and EV7-EV12, legends should be uploaded as a separate tab/sheet in each Excel file

- APPENDIX 1 FILE WITH ToC: figure labels should be corrected to Appendix Figure S1-S7 instead of Carey et al, Appendix Figure 1-7

- Please provide suggestions for a short 'blurb' text prefacing and summing up the conceptual aspect of the study in two sentences (max. 250 characters), followed by 3-5 one-sentence 'bullet points' with brief factual statements of key results of the paper; they will form the basis of an editor-written 'Synopsis' accompanying the online version of the article. Please also provide an altered synopsis image, making sure that the aspect ratio conforms to our website's format - it should be exactly 550 pixels wide and 300 pixels high.

- Figure Legends (main + EV):
1. Please indicate the statistical test used for data analysis in the legends of figures 2B, 3C, D; 4E

2. Please note that information related to n is missing in the legends of figures 2B, 3B, 6B, EV2 A, EV4 C

- "Character count" should be removed from title page

- Tables EV5-EV6 and EV13 should be renumbered to Table EV1-EV3 with legends included above tables in each Excel file (the legend is fine for Table EV13 in word doc)

- 2 movie files. The legends should be removed from the manuscript and zipped with each movie file.

- Sections need to be named and the order should be corrected: Title page - Abstract - Keywords - Introduction - Results - Discussion - Methods - Data Availability - Acknowledgements - Disclosure and Competing Interests Statement - References - Figure Legends - Table(s) - Expanded View Figure Legends.

Please let me know if you have any questions regarding any of these points. You can use the link below to upload the revised files.
Thank you again for giving us the chance to consider your manuscript for The EMBO Journal. I look forward to receiving the final version.
With best regards,
Cornelius Schneider

Cornelius Schneider, PhD
Editor | The EMBO Journal
c.schneider@embojournal.org

Please refer to our figure preparation guideline in order to ensure proper formatting and readability in print as well as on screen:

https://link.springer.com/journal/44318/submission-guidelines#cms-Figure-and-data-presentation

Use the link below to submit your revision:

https://emboj.msubmit.net/cgi-bin/main.plex

Referee #1:

The authors have nicely answered our previous concerns, we now recommend that the manuscript be accepted.

All editorial and formatting issues were resolved by the authors.

3rd Revision - Editorial Decision **10th Jun 2026**

Dear Dr. Ruiz,

I am pleased to inform you that your manuscript has been accepted for publication in the EMBO Journal.

Your manuscript will be processed for publication by EMBO Press. It will be copy edited and you will receive page proofs prior to publication. Please note that you will be contacted by Springer Nature Author Services to complete licensing and payment information.

You may qualify for financial assistance for your publication charges - either via a Springer Nature fully open access agreement or an EMBO initiative. Check your eligibility: https://link.springer.com/journal/44318/how-to-publish-with-us

Should you be planning a Press Release on your article, please notify the production team via this form in order to coordinate publication and release dates: https://forms.cloud.microsoft/e/eH4A8JVP6g

If you have any questions, please do not hesitate to contact the Editorial Office. Thank you for your contribution to The EMBO Journal.

Yours sincerely,

Cornelius Schneider, PhD
Editor
The EMBO Journal
c.schneider@embojournal.org

*****
Please note that it is The EMBO Journal policy for the transcript of the editorial process (containing referee reports and your response letters) to be published as an online supplement to each paper. If you should prefer removal of any referee-only figures included in the point-by-point response(s), e.g. because they may still be used for future publication or because they have been reproduced from published work by others, please do let us know immediately via response email.
More information is available here: https://link.springer.com/partners/embo-press/editorial-policies#Peer%20review

** Click here to be directed to your login page: https://emboj.msubmit.net

EMBO Press Author Checklist

Corresponding Author Name: SERGIO RUIZ MACIAS	
Journal Submitted to: THE EMBO JOURNAL	
Manuscript Number: EMBOJ-2025-121165R-Q	

USEFUL LINKS FOR COMPLETING THIS FORM
The EMBO Journal - Author Guidelines
EMBO Reports - Author Guidelines
Molecular Systems Biology - Author Guidelines
EMBO Molecular Medicine - Author Guidelines

Reporting Checklist for Life Science Articles (updated January
This checklist is adapted from Materials Design Analysis Reporting (MDAR) Checklist for Authors. MDAR establishes a minimum set of requirements in transparent reporting in the life sciences (see Statement of Task: 10.31222/osf.io/9sm4x). Please follow the journal's guidelines in preparing your
Please note that a copy of this checklist will be published alongside your article.

Abridged guidelines for figures
1. Data
The data shown in figures should satisfy the following conditions:
- the data were obtained and processed according to the field's best practice and are presented to reflect the results of the experiments in an accurate and unbiased manner.
- ideally, figure panels should include only measurements that are directly comparable to each other and obtained with the same assay.
- plots include clearly labeled error bars for independent experiments and sample sizes. Unless justified, error bars should not be shown for technical
- if n<5, the individual data points from each experiment should be plotted. Any statistical test employed should be justified.
- Source Data should be included to report the data underlying figures according to the guidelines set out in the authorship guidelines on Data

2. Captions
Each figure caption should contain the following information, for each panel where they are relevant:
- a specification of the experimental system investigated (eg cell line, species name).
- the assay(s) and method(s) used to carry out the reported observations and measurements.
- an explicit mention of the biological and chemical entity(ies) that are being measured.
- an explicit mention of the biological and chemical entity(ies) that are altered/varied/perturbed in a controlled manner.
- the exact sample size (n) for each experimental group/condition, given as a number, not a range;
- a description of the sample collection allowing the reader to understand whether the samples represent technical or biological replicates (including how many animals, litters, cultures, etc.).
- a statement of how many times the experiment shown was independently replicated in the laboratory.
- definitions of statistical methods and measures:
  - common tests, such as t-test (please specify whether paired vs. unpaired), simple χ2 tests, Wilcoxon and Mann-Whitney tests, can be unambiguously identified by name only, but more complex techniques should be described in the methods section;
  - are tests one-sided or two-sided?
  - are there adjustments for multiple comparisons?
  - exact statistical test results, e.g., P values = x but not P values < x;
  - definition of 'center values' as median or average;
  - definition of error bars as s.d. or s.e.m.

Please complete ALL of the questions below.
Select "Not Applicable" only when the requested information is not relevant for your study.

Materials

Newly Created Materials	Information included in the manuscript?	In which section is the information available? (Reagents and Tools Table, Materials and Methods, Figures, Data Availability Section)
New materials and reagents need to be available; do any restrictions apply?	Not Applicable	There are no restrictions to make available the new material and reagents generated in this work

Antibodies	Information included in the manuscript?	In which section is the information available? (Reagents and Tools Table, Materials and Methods, Figures, Data Availability Section)
For **antibodies** provide the following information:<br>- Commercial antibodies: RRID (if possible) or supplier name, catalogue number and or/clone number<br>- Non-commercial: RRID or citation	Yes	Reagents Tools Table

DNA and RNA sequences	Information included in the manuscript?	In which section is the information available? (Reagents and Tools Table, Materials and Methods, Figures, Data Availability Section)
**Short novel DNA or RNA including primers, probes**: provide the sequences.	Yes	Table EV13

Cell materials	Information included in the manuscript?	In which section is the information available? (Reagents and Tools Table, Materials and Methods, Figures, Data Availability Section)
**Cell lines:** Provide species information, strain. Provide accession number in repository **OR** supplier name, catalog number, clone number, and/**OR** RRID.	Yes	Reagents Tools Table and Methods
**Primary cultures:** Provide species, strain, sex of origin, genetic modification status.	Not Applicable	
Report if the cell lines were recently **authenticated** (e.g., by STR profiling) and tested for mycoplasma contamination.	Yes	Cells were not authenticated but they were tested for mycoplasma contamination

Experimental animals	Information included in the manuscript?	In which section is the information available? (Reagents and Tools Table, Materials and Methods, Figures, Data Availability Section)
**Laboratory animals or Model organisms:** Provide species, strain, sex, age, genetic modification status. Provide accession number in repository **OR** supplier name, catalog number, clone number, **OR** RRID.	Not Applicable	
**Animal observed in or captured from the field:** Provide species, sex, and age where possible.	Not Applicable	
Please detail **housing and husbandry conditions**.	Not Applicable	

Plants and microbes	Information included in the manuscript?	In which section is the information available? (Reagents and Tools Table, Materials and Methods, Figures, Data Availability Section)
**Plants:** provide species and strain, ecotype and cultivar where relevant, unique accession number if available, and source (including location for collected wild specimens).	Not Applicable	
**Microbes:** provide species and strain, unique accession number if available, and source.	Not Applicable	

Human research participants	Information included in the manuscript?	In which section is the information available? (Reagents and Tools Table, Materials and Methods, Figures, Data Availability Section)
If collected and within the bounds of privacy constraints report on age, sex and gender or ethnicity for all study participants.	Not Applicable	

Core facilities	Information included in the manuscript?	In which section is the information available? (Reagents and Tools Table, Materials and Methods, Figures, Data Availability Section)
If your work benefited from core facilities, was their service mentioned in the acknowledgments section?	Yes	Acknowledgments

Design

Study protocol	Information included in the manuscript?	In which section is the information available? (Reagents and Tools Table, Materials and Methods, Figures, Data Availability Section)
If study protocol has been **pre-registered, provide DOI in the manuscript**. For clinical trials, provide the trial registration number **OR** cite DOI.	Not Applicable	
Report the **clinical trial registration number** (at ClinicalTrials.gov or equivalent), where applicable.	Not Applicable	

Laboratory protocol	Information included in the manuscript?	In which section is the information available? (Reagents and Tools Table, Materials and Methods, Figures, Data Availability Section)
Provide DOI OR other citation details if **external detailed step-by-step protocols** are available.	Not Applicable	

Experimental study design and statistics	Information included in the manuscript?	In which section is the information available? (Reagents and Tools Table, Materials and Methods, Figures, Data Availability Section)
Include a statement about **sample size** estimate even if no statistical methods were used.	Yes	Statistical Analysis
Were any steps taken to minimize the effects of subjective bias when allocating animals/samples to treatment (e.g. **randomization procedure**)? If yes, have they been described?	Not Applicable	
Include a statement about **blinding** even if no blinding was done.	Yes	Statistical Analysis
Describe **inclusion/exclusion criteria** if samples or animals were excluded from the analysis. Were the criteria pre-established?<br><br>If sample or data points were omitted from analysis, report if this was due to attrition or intentional exclusion and provide justification.	Not Applicable	Statistical Analysis (No data were excluded from the analyses)
For every figure, are **statistical tests** justified as appropriate? Do the data meet the assumptions of the tests (e.g., normal distribution)? Describe any methods used to assess it. Is there an estimate of variation within each group of data? **I**s the variance similar between the groups that are being statistically compared?	Yes	Statistical Analysis

Sample definition and in-laboratory replication	Information included in the manuscript?	In which section is the information available? (Reagents and Tools Table, Materials and Methods, Figures, Data Availability Section)
In the figure legends: state number of times the experiment was **replicated** in laboratory.	Yes	Figure Legends
In the figure legends: define whether data describe **technical or biological replicates**.	Yes	Figure Legends

Ethics

Ethics	Information included in the manuscript?	In which section is the information available? (Reagents and Tools Table, Materials and Methods, Figures, Data Availability Section)
Studies involving **human participants**: State details of **authority granting ethics approval** (IRB or equivalent committee(s), provide reference number for approval.	Not Applicable	
Studies involving **human participants**: Include a statement confirming that **informed consent** was obtained from all subjects and that the experiments conformed to the principles set out in the WMA Declaration of Helsinki and the Department of Health and Human Services Belmont Report.	Not Applicable	
Studies involving **human participants:** For publication of **patient photos**, include a statement confirming that consent to publish was obtained.	Not Applicable	
Studies involving experimental **animals**: State details of **authority granting ethics approval** (IRB or equivalent committee(s), provide reference number for approval. Include a statement of compliance with ethical regulations.	Not Applicable	
Studies involving **specimen and field samples:** State if relevant **permits** obtained, provide details of authority approving study; if none were required, explain why.	Not Applicable	

Dual Use Research of Concern (DURC)	Information included in the manuscript?	In which section is the information available? (Reagents and Tools Table, Materials and Methods, Figures, Data Availability Section)
Could your study fall under dual use research restrictions? Please check biosecurity documents and list of **select agents and toxins** (CDC): https://www.selectagents.gov/sat/list.htm	Not Applicable	
If you used a select agent, is the security level of the lab appropriate and reported in the manuscript?	Not Applicable	
If a study is subject to dual use research of concern regulations, is the name of the **authority granting approval and reference number** for the regulatory approval provided in the manuscript?	Not Applicable	

Reporting

The MDAR framework recommends adoption of discipline-specific guidelines, established and endorsed through community initiatives. Journals have their own policy about requiring specific guidelines and recommendations to complement MDAR.

Adherence to community standards	Information included in the manuscript?	In which section is the information available? (Reagents and Tools Table, Materials and Methods, Figures, Data Availability Section)
State if relevant guidelines or checklists (e.g., **ICMJE, MIBBI, ARRIVE, PRISMA**) have been followed or provided.	Not Applicable	
For **tumor marker prognostic studies,** we recommend that you follow the **REMARK** reporting guidelines (see link list at top right). See author guidelines, under 'Reporting Guidelines'. Please confirm you have followed these guidelines.	Not Applicable	
For **phase II and III randomized controlled trials**, please refer to the **CONSORT** flow diagram (see link list at top right) and submit the CONSORT checklist (see link list at top right) with your submission. See author guidelines, under 'Reporting Guidelines'. Please confirm you have submitted this list.	Not Applicable	

Data Availability

Data availability	Information included in the manuscript?	In which section is the information available? (Reagents and Tools Table, Materials and Methods, Figures, Data Availability Section)
Have **primary datasets** been deposited according to the journal's guidelines (see 'Data Deposition' section) and the respective accession numbers provided in the Data Availability Section?	Yes	Data Availability Section
Were **human clinical and genomic datasets** deposited in a public access-controlled repository in accordance to ethical obligations to the patients and to the applicable consent agreement?	Not Applicable	
Are **computational models** that are central and integral to a study available without restrictions in a machine-readable form? Were the relevant accession numbers or links provided?	Not Applicable	
If publicly available data were reused, provide the respective **data citations in the reference list**.	Yes	References
