# Supplementary material for: Alterations in chromatin organization promote totipotent-like features in a DPPA2/DUX-dependent manner
Source: EMBO J. 2026 Jul 9;45(16):5657–88. doi: 10.1038/s44318-026-00853-6 (PMC13484482; doi:10.1038/s44318-026-00853-6)
Supplement: Supplementary file 4 [file 44318_2026_853_MOESM4_ESM.docx]

**Table EV3: List of primers.**

| **Primers (qPCR)** |  |  |
| --- | --- | --- |
|  | forward | reverse |
| DUX | GGAGTGAGAGGCAGATCAGG | CTGCTGACCGAAGTCCAACT |
| ZSCAN4 | TCTTTCTGGTTGGCAGCTTT | GCCAGGCTTCTGTCAAGAAC |
| ZFP352 | AAGGTCCCACATCTGAAGAA | GGGTATGAGGATTCACCCA |
| TCSTV3 | ACCAGCTGAAACATCCATCC | CCATGGATCCCTGAAGGTAA |
| SP110 | CACCTGCAAACAAGAAAGCA | AACTCCATGTCCAGGTGAGG |
| TDPOZ1 | GCCCTTGATTTCATTGCCTA | CACTTTCGGCTCCAAGAAAG |
| DUB1 | GCCTTCAAGTGCACAGACAA | GATGTCCAGGAAGCGATCAT |
| EIF1ad8 | AACAGGCGCAGAGGTAAAAA | GCACAGCCTCCTTACACCAT |
| MERVL | ATCTCCTGGCACCTGGTATG | AGAAGAAGGCATTTGCCAGA |
| NANOG | CAGGTGTTTGAGGGTAGCTC | CGGTTCATCATGGTACAGTC |
| VIMENTIN | TGAAGGAAGAGATGGCTCGT | TCCAAGCAGCTTCCTGTAGGT |
| NESTIN | CTCGAGCAGGAAGTGGTAGG | CTCGAGCAGGAAGTGGTAGG |
| PRDM14 | CACTCCCGAAGTACCACTGATT | CCCACCTCTGACCACTGATT |
| GAPDH | TTCACCACCATGGAGAAGGC | CCCTTTTGGCTCCACCCT |
|  |  |  |
| **Primers (Cloning)** |  |  |
| **pVector-hCTCF_FKBP_2A_eGFP Construct** | | |
| FKBP-2A-eGFP-F | TGGACCGGGGGGGAGCAGGAGGCGTCGACGGTATC | |
| FKBP-2A-eGFP-R | ACGCACAAGGCTCCGCCATTACTTGTACAGCTCGTCCATGCCG | |
| VECTOR-F1 | TAATGGCGGAGCCTTGTGCGTCGCCAGGACTTCTCTGGGCTG | |
| VECTOR-R1 | TCCTGCTCCCCCCCGGTCCATCATGCTGAGGATCATCT | |
|  |  |  |
| **pVector-mWAPL_FKBP_2A_eGFP Construct** | | |
| mWAPL-5’-F | TTGGAGCTGTCTTCTAATTTACCTTTGTAGCATGG | |
| mWAPL-5’-R | TGCTCCCCCGCAATGTTCCAAATATTCAATCACTCTAG | |
| FKBP-2A-eGFP-F | GGAACATTGCGGGGGAGCAGGAGGCGTCGACGGTAT | |
| FKBP-2A-eGFP-R | GGTAAAGCAGTTACTTGTACAGCTCGTCCATGCCGAGAG | |
| mWAPL-3’-F | GTACAAGTAACTGCTTTACCTTTGCTTCAGGTGCTTGG | |
| mWAPL-3’-R | CAGTTTTACAAATCCAATGAAGAAACATGATGTAAC | |
| VECTOR-F | CATTGGATTTGTAAAACTGTTTTTTGCTTTTGCTTTTCCCTGACTCCC | |
| VECTOR-R | ATTAGAAGACAGCTCCAATTCGCCCTATAGTGAGTCG | |
|  |  | |
| **pVector-hWAPL_FKBP_2A_eGFP Construct** | | |
| hWAPL-5’-F | TTGGAGCTTGCTAATAATGAAGGGTTTTCTCTCC | |
| hWAPL-5’-R | GCTCCCCCGCAATGTTCCAAATATTCAATCACTC | |
| FKBP-2A-eGFP-F | TGGAACATTGCGGGGGAGCAGGAGGCGTCGACGGTAT | |
| FKBP-2A-eGFP-R | GGTAAAGCAGTTACTTGTACAGCTCGTCCATGCCG | |
| hWAPL-3’-F | GTACAAGTAACTGCTTTACCTTTGCTTCAGGTGCTCGG | |
| hWAPL-3’-R | CAGTTTGGGTCTTCTCTAAACCAGCAATTTAAG | |
| VECTOR-F | GTTTAGAGAAGACCCAAACTGTTTTTTGCTTTTGCTTTTCCCTGAC | |
| VECTOR-R | CATTATTAGCAAGCTCCAATTCGCCCTATAGTGAGTC | |
|  |  | |
| **Primers (CRISPR targeting)** | | |
| pLentiCRISPRV2_hWAPL | CACCGTTACCGAGCACCTGAAGCAA | AAACTTGCTTCAGGTGCTCGGTAAC |
| pLentiCRISPRV2_mWAPL | CACCGTTACCAAGCACCTGAAGCAA | AAACTTGCTTCAGGTGCTTGGTAAC |
| pLentiCRISPRV2_hCTCF | CACCGAGTGTCCAACAGGTTGAGCC | AAACCAAGATCTATCTGTCTACATC |
| pLentiCRISPRV2_mCTCF-1 | CACCGATCACCGGTCCATCATGCTG | AAACCAGCATGATGGACCGGTGATC |
| pLentiCRISPRV2_mCTCF-2 | CACCGCTGGGGCCTTGCTCGGCAC | AAACGGTGCCGAGCAAGGCCCCAGC |
| pLentiCRISPRV2_ARID3A-1 | CACCGCAGAGGGAGAAACTGATGTG | AAACCACATCAGTTTCTCCCTCTGC |
| pLentiCRISPRV2_ARID3A-2 | CACCGCCTCATGTGCAAAGATCCTG | AAACCAGGATCTTTGCACATGAGGC |
| pLentiCRISPRV2_ARID3A-3 | CACCGGGTGATCAAAGGCCCCCAA | AAACTTGGGGGCCTTTGATCACCC |
| pX458_DPPA2-1 | CACCGAGAAAGTGTGATTTTAACGC | AAACGCGTTAAAATCACACTTTCTC |
| pX458_DPPA2-2 | CACCGACCTTAGACCACACACCACC | AAACGGTGGTGTGTGGTCTAAGGTC |
| pX458_DPPA2-3 | CACCGTAACTTGAGTACGGATGGCA | AAACTGCCATCCGTACTCAAGTTAC |
|  | | |
| **Primers (Genotyping)** |  | AAACTAGCAGTAGGTTTAAGCCTC |
| ARID3A-KO | GAAAGCCAGTGAGGGTCACTGAGG | CGCACAAACTGGTTCTCTCCCGG |
|  | | |
| **Probes FISH** | | |
| DUX_FISH_1 | GGAATCCCGGCGGCGCAGGAAGACGGTTTGATACTCAGTGGCGGCCAATGTCAACTCTGCATCTT | |
| DUX_FISH_2 | GGTCTCAGATTGCCGCATCCGCGTGTGGTTATACTCAGTGGCGGCCAATGTCAACTCTGCATCTT | |
| DUX_FISH_3 | GGGGCATGCCTCAAAGAGGTCCATCAGAGGATACTCAGTGGCGGCCAATGTCAACTCTGCATCTT | |
| DUX_FISH_4 | CGCTCATCTGGCAGAAGGCCTCGCACTCGAATACTCAGTGGCGGCCAATGTCAACTCTGCATCTT | |
| DUX_FISH_5 | CAAGCCTTTGAGAGGAACCCACGACCAGGCATACTCAGTGGCGGCCAATGTCAACTCTGCATCTT | |
| DUX_FISH_6 | ACAGGGTTGCCCGAGGACACGATCCACATAATACTCAGTGGCGGCCAATGTCAACTCTGCATCTT | |
| DUX_FISH_7 | CAAGACTTGCTGGCGTCACAAGGGTCGGATATACTCAGTGGCGGCCAATGTCAACTCTGCATCTT | |
| DUX_FISH_8 | GGTGCCCAGGAGAACTTGTTGCCACAGGAAATACTCAGTGGCGGCCAATGTCAACTCTGCATCTT | |
| DUX_FISH_9 | GCCCTAGCGACTTGCCCTCCTTCTGCGGAGATACTCAGTGGCGGCCAATGTCAACTCTGCATCTT | |
| DUX_FISH_10 | CCCCACTCAAGCAGGCAACGCAGGCTCTCTATACTCAGTGGCGGCCAATGTCAACTCTGCATCTT | |
| DUX_FISH_11 | GTGCATGAAGGTTCCCAGGAGAGCTTTTGGCATACTCAGTGGCGGCCAATGTCAACTCTGCATCTT | |
| DUX_FISH_12 | TCTAGCCCCAGCGACTCAAACTCCTTCTGCATACTCAGTGGCGGCCAATGTCAACTCTGCATCTT | |
| DUX_FISH_13 | GAGCGGGCCAAGAAGATGCCCGCACTCAAGATACTCAGTGGCGGCCAATGTCAACTCTGCATCTT | |
| DUX_FISH_14 | TCCTTGATCAACTGCTGGACGAAGTCCAAAAGGAATACTCAGTGGCGGCCAATGTCAACTCTGCATCTT | |
| DUX_FISH_15 | TCCCAGGACAGCTTACTGCCCCTGGAGGAAATACTCAGTGGCGGCCAATGTCAACTCTGCATCTT | |
| DUX_FISH_16 | AGCATCTGGCCAACCTTCTGCAGAGAATCCATACTCAGTGGCGGCCAATGTCAACTCTGCATCTT | |
| DUX_FISH_17 | CCCCTGGAGCTCTTCCTCTATCAACTGTTGGAATACTCAGTGGCGGCCAATGTCAACTCTGCATCTT | |
| DUX_FISH_18 | CTCTGAATTGGGATGTAGATCCTGGTGGCAGGGATACTCAGTGGCGGCCAATGTCAACTCTGCATCTT | |
| DUX_FISH_19 | TACTTCAAGCCCCAGCGACTCAAACTCCTTCTATACTCAGTGGCGGCCAATGTCAACTCTGCATCTT | |
| DUX_FISH_20 | GAGCATGTGCCAGCCCCACTGGATTGGGGTAATACTCAGTGGCGGCCAATGTCAACTCTGCATCTT | |
| DUX_FISH_21 | CAGGACAGCTTACTGCCCCTGGAGGAAGCAATACTCAGTGGCGGCCAATGTCAACTCTGCATCTT | |
| DUX_FISH_22 | AGCATCTGGCCAGCCTTCTGCAGAAAATCCATACTCAGTGGCGGCCAATGTCAACTCTGCATCTT | |
| DUX_FISH_23 | CCCCTGGAGCTCTTCCTTGATCAACTGCTGATACTCAGTGGCGGCCAATGTCAACTCTGCATCTT | |
| DUX_FISH_24 | GCCCCTGTGAATGTGGAGGAAACATGGGAGATACTCAGTGGCGGCCAATGTCAACTCTGCATCTT | |
| DUX_FISH_25 | ATCAGACTCTTCTAGATATGCTCTGACTCCCCGACAGATACTCAGTGGCGGCCAATGTCAACTCTGCATCTT | |
| DUX_FISH_readout | TAAAGATGCAGAGTTGACATTGGCCGCCACTGAG | |
|  |  | |
