# Supplementary material for: Alterations in chromatin organization promote totipotent-like features in a DPPA2/DUX-dependent manner
Source: EMBO J. 2026 Jul 9;45(16):5657–88. doi: 10.1038/s44318-026-00853-6 (PMC13484482; doi:10.1038/s44318-026-00853-6)
Supplement: Supplementary file 5 [file 44318_2026_853_MOESM16_ESM.zip › EMBOJ-2025-121165R-Q_EV_Movie_1/EMBOJ-2025-121165R-Q_Legend_Movie EV1.docx]

**Movie EV1:** 3D reconstruction of untreated mESC^CTCF-AID^ obtained from a FIB-SEM derived image stack. Scale bar: 5μm.
