# Supplementary material for: Alterations in chromatin organization promote totipotent-like features in a DPPA2/DUX-dependent manner
Source: EMBO J. 2026 Jul 9;45(16):5657–88. doi: 10.1038/s44318-026-00853-6 (PMC13484482; doi:10.1038/s44318-026-00853-6)
Supplement: Supplementary file 6 [file 44318_2026_853_MOESM17_ESM.zip › EMBOJ-2025-121165R-Q_EV_Movie_2/EMBOJ-2025-121165R-Q_Legend_Movie EV2.docx]

**Movie EV2:** 3D reconstruction of IAA-treated mESC^CTCF-AID^ for 48 hours obtained from a FIB-SEM derived image stack. Scale bar: 5μm.
